# Catalyst-Switchable Regiocontrol in the Direct Arylation of Remote C–H Groups in Pyrazolo[1,5-*a*]pyrimidines**

**DOI:** 10.1002/anie.201502150

**Published:** 2015-06-10

**Authors:** Robin B Bedford, Steven J Durrant, Michelle Montgomery

**Affiliations:** School of Chemistry, University of BristolCantock's Close, Bristol, BS8 1TS (UK); Vertex Pharmaceuticals Ltd. (Europe)86-88 Jubilee Avenue, Milton Park, Abingdon, Oxfordshire, OX14 4RW (UK)

**Keywords:** arylation, catalysis, C–H activation, palladium, regiocontrol

## Abstract

The regiodivergent palladium-catalyzed C–H arylation of pyrazolo[1,5-*a*]pyrimidine has been achieved, wherein the switch in regioselectivity between positions C3 and C7 is under complete catalyst control. A phosphine-containing palladium catalyst promotes the direct arylation at the most acidic position (C7), whereas a phosphine-free catalyst targets the most electron-rich position (C3).

The direct C–H arylation of heterocyclic substrates (Scheme 1) is a powerful synthetic tool for the construction of functionalized heterocycles. It maintains the expediency associated with simple cross-coupling reactions, but with greater step economy and lower waste production.[1] In heterocycles with multiple C–H groups, it would be highly advantageous to be able to choose which C–H group is functionalized, ideally with complete selectivity and with the ability to “switch” regioselectivity at will.[2] Seminal examples of this approach include the vinylation or arylation of pyrroles and indoles at either C2 or C3, where the outcome is driven by a change in substrate derivatization,[3, 4] solvent,[5] or oxidant.[6]

**Scheme 1:**
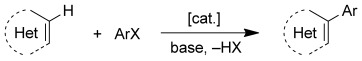
The direct C–H arylation of heterocycles.

In these cases, the site selectivity is typically controlled by prohibiting or encouraging migration between positions C3 and C2. By contrast, we were interested to find out whether site selectivity could be engendered in the arylation of remote C–H groups, and whether catalyst “tuning” could be employed to drive this selectivity.[7]

We report herein the catalyst-controlled switching in regioselectivity between the remote positions C3 and C7 of pyrazolo[1,5-*a*]pyrimidine (**1**; Figure 1). We chose this motif[8] because it forms the core of a range of biologically active compounds. Specifically, aryl-substituted pyrazolo[1,5-*a*]pyrimidines show antitumor[9] and anti-inflammatory properties,[10] have been examined as hepatitis C virus inhibitors and estrogen receptor ligands,[11, 12] and are found in the approved sedative agents zaleplon and indiplon as well as in the anxiolytic agent ocinaplon.

**Figure 1:**
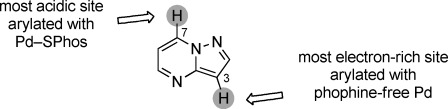
Catalyst-driven site selectivity in the arylation of pyrazolo[1,5-*a*]pyrimidine (1).


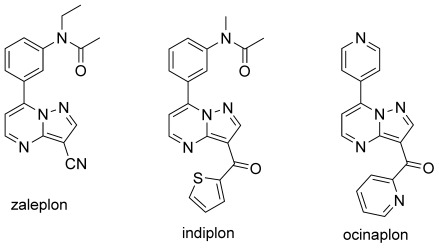


We began our study with the optimization of the coupling of compound **1** with bromobenzene in the presence of a variety of palladium sources, with and without phosphines, changing solvents, bases, additives, and conditions. The results from this survey are summarized in the Supporting Information, and Scheme 2 highlights the optimized conditions.

**Scheme 2:**
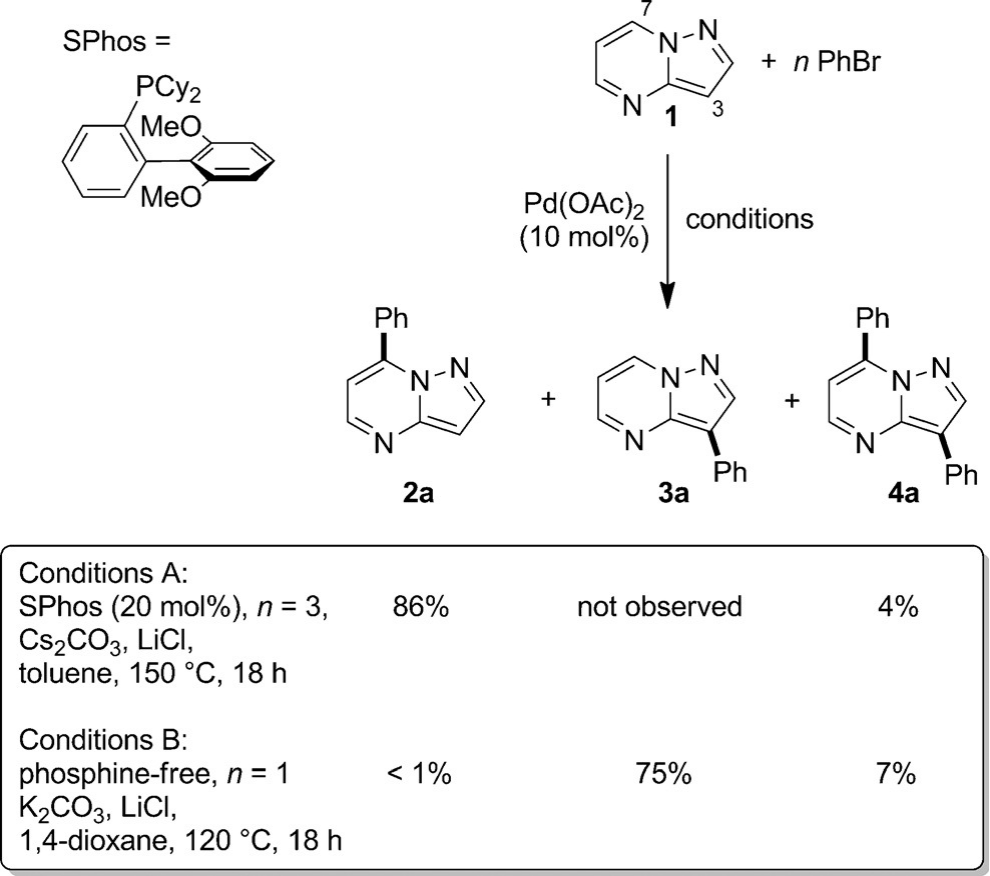
Results of optimization of reaction conditions. Yields determined by ^1^H NMR spectroscopy (1,3,5-(MeO)_3_C_6_H_3_ used as internal standard).

The use of the monodentate phosphine ligand SPhos proved crucial to achieving site selectivity at C7, giving the desired product **2 a** in good yield.[13] NMR and UHPLC/ES-MS analyses of the crude reaction mixture showed a trace amount (4 %) of the 3,7-diphenylated product (**4 a**) but none of the 3-arylated species **3 a**, thus indicating that arylation at C3 can only occur (to a very limited extent) after arylation at C7.[14] Conveniently, the reaction works just as well under air as under an inert atmosphere. The relatively high air stability of SPhos is obviously a pivotal factor here.[15]

By contrast, the use of palladium acetate alone, in the absence of a phosphine co-ligand, led to selective arylation at C3, giving **3 a** in good yield. The arylation at C3 is marginally less selective, giving small amounts of **2 a** and **4 a**.[14]

The addition of a salt such as lithium chloride proved essential for maintaining a good yield in the SPhos-containing reaction, with only 40 % of **2 a** obtained in its absence, while in the phosphine-free case omitting the salt led to a pronounced decrease in selectivity with significant amounts of **2 a** (10 %) and the diarylated product **4 a** (12 %) obtained in addition to the desired product **3 a** (50 %).[16]

With the optimized conditions to hand, we next examined the range of aryl bromides that could be exploited in the C7-selective reaction (Figure 2).[17] Electron-donating, electron-withdrawing, and sterically demanding substrates were all tolerated under the reaction conditions and the products (**2 a**–**l**) were isolated in moderate to excellent yields. Gratifyingly, ester and nitro functionalities were tolerated under the reaction conditions (**2 f** and **i**). Aryl chlorides do not participate in the reaction, thus allowing them to be incorporated into the product (**2 g**) and therefore offering an opportunity for subsequent functionalization. In addition, heteroaryl bromides are compatible with the reaction conditions, enabling the preparation of compounds **2 m**–**p** in one step, in moderate to good yields, from commercial starting materials.

**Figure 2:**
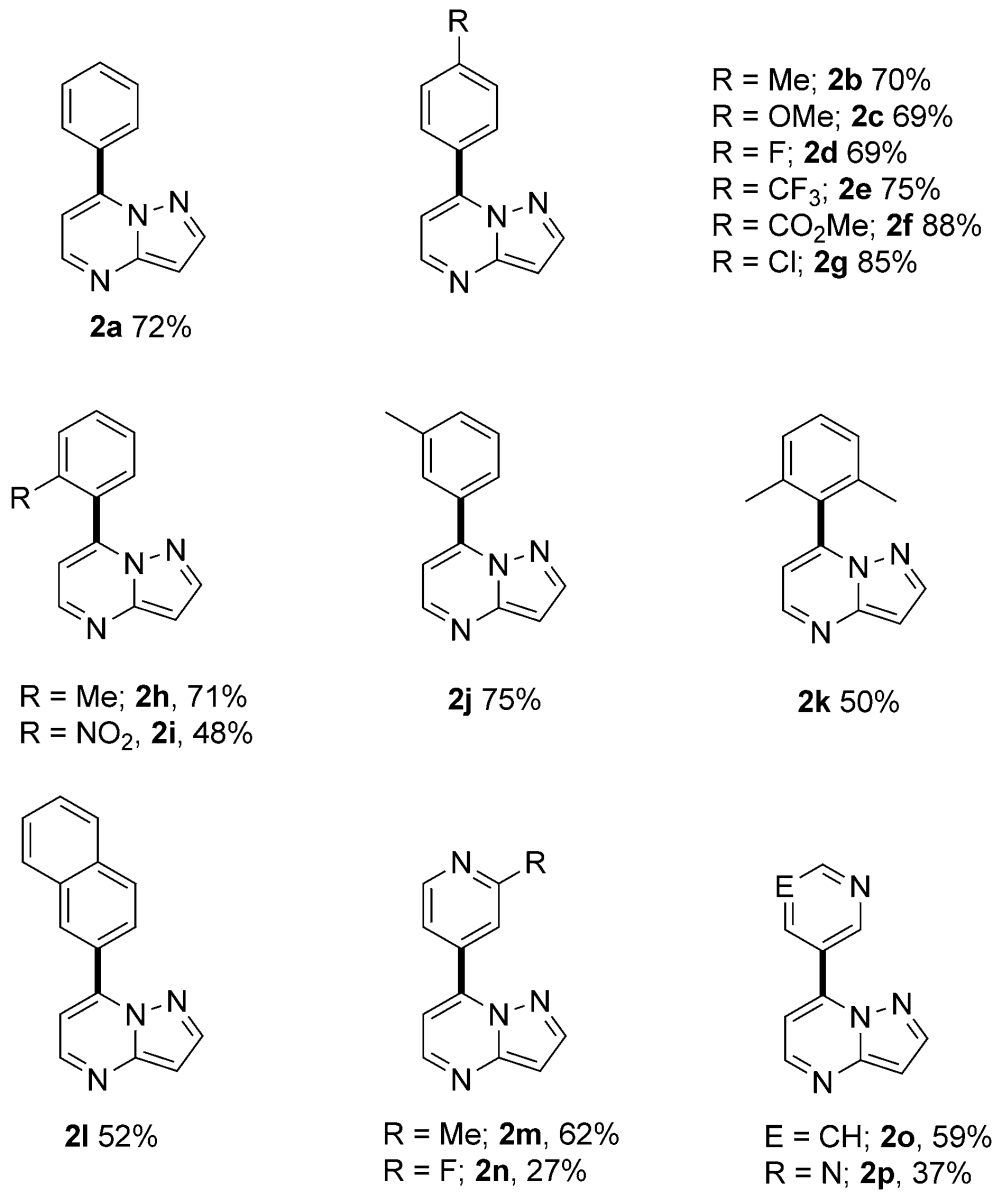
Products of the C7 arylation of compound 1 (0.25 mmol) with aryl bromides under reaction conditions A (Scheme 2).

Figure 3 summarizes the C3-selective arylation reactions with a range of aryl bromides under phosphine-free conditions.[17] Again, electron-donating, electron-withdrawing, sterically demanding, and heteroaryl bromides were all tolerated under the reaction conditions, giving isolated 3-arylpyrazolo[1,5-a]pyrimidines (**3 a**–**q**) in moderate to excellent yields. In addition, 3-bromobenzonitrile, which did not react in the C7 arylation, was tolerated under the reaction conditions to afford **3 k** in good yield.

**Figure 3:**
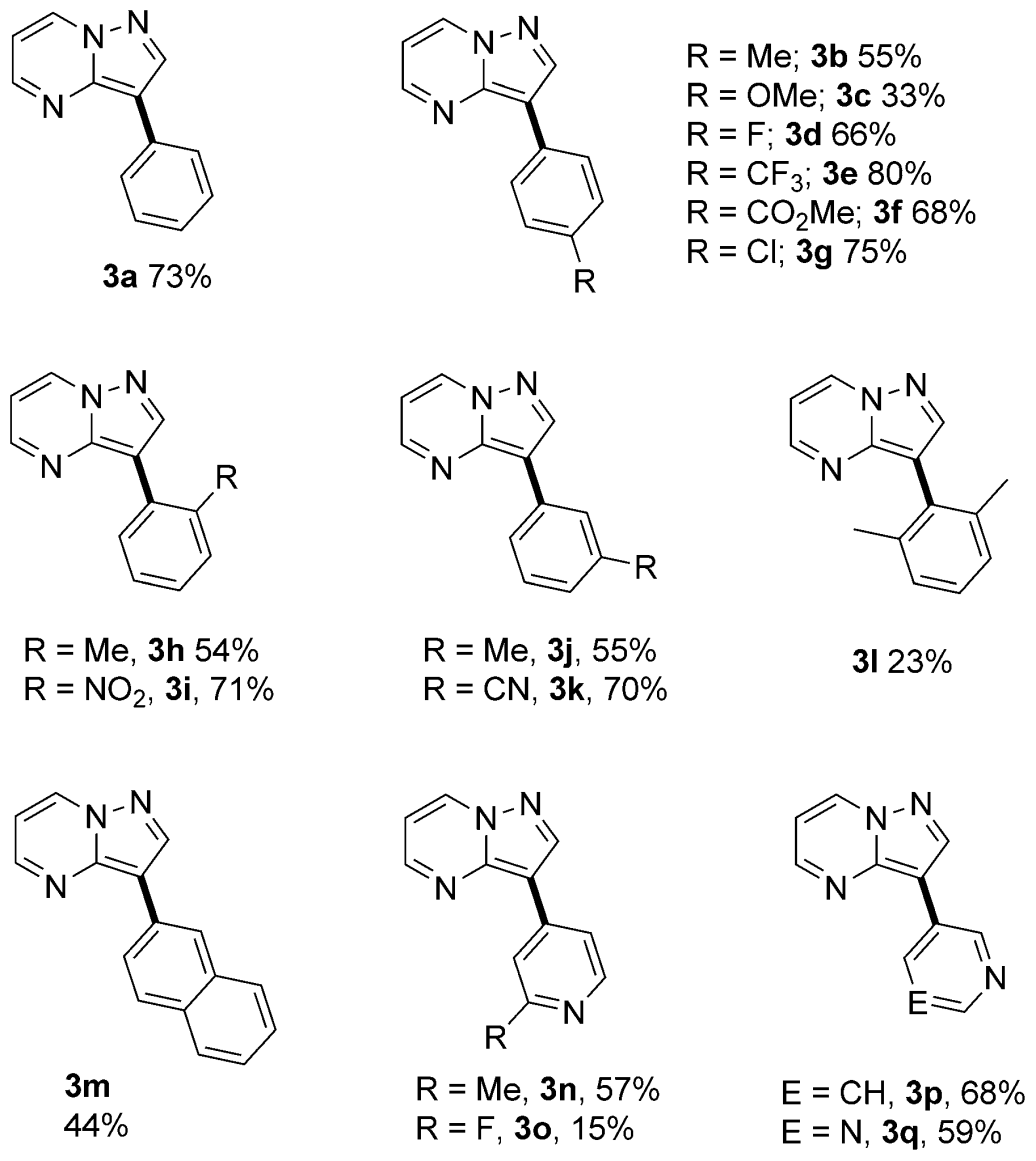
Products of the C3 arylation of compound 1 (0.25 mmol) with aryl bromides under reaction conditions B (Scheme 2).

Selected products of the C3- or C7-arylation reactions can subsequently be arylated in the alternative position in reasonable yields (Scheme 3).

**Scheme 3:**
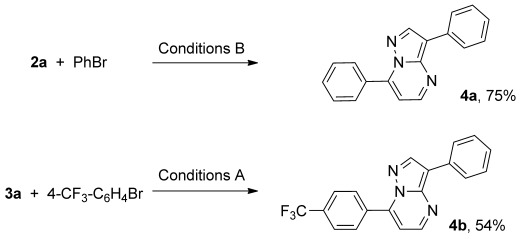
Sequential arylation reactions, for conditions see Scheme 2.

Finally we turned our attention to gleaning preliminary mechanistic insights. It is clear from previous reports that the natural site of electrophilic substitution is the C3 position,[18] while a simple deuteration experiment (Scheme 4) confirmed that the most acidic site is the C7 position. These empirical observations are supported by density functional theory (DFT) calculations.[19] On the one hand, these showed that the HOMO orbital has significant electron density at the C3 position, and that this position has by far the highest “electrophile affinity” (Eα) value (Figure 4 a),[20] on the other hand, they highlight the significantly greater acidity of the C7–H group as determined by comparing the calculated relative energies for the removal of each of the protons by acetate (Figure 4 b).

**Figure 4:**
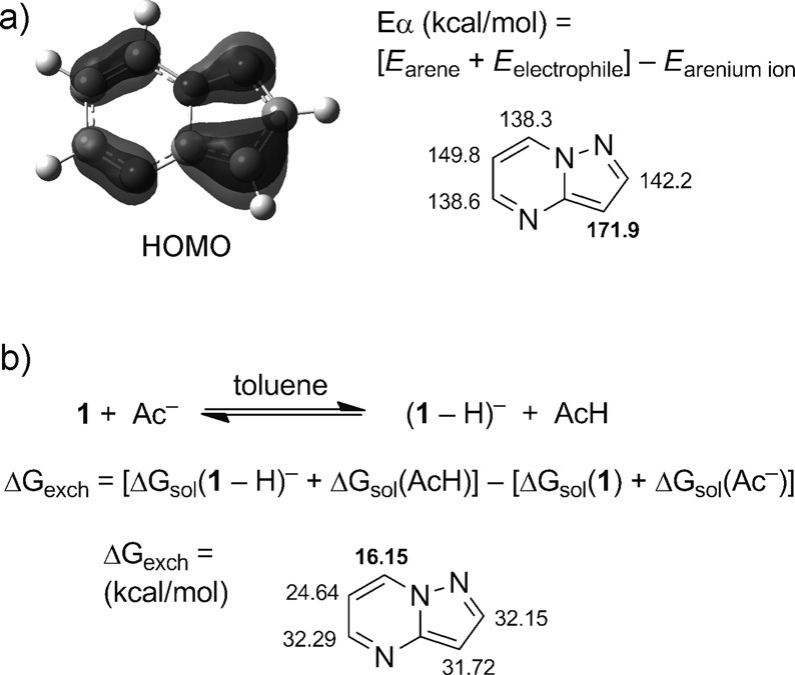
Summary of DFT computational results. a) Left: calculated (B3LYP-d2/6-311++G(d)) HOMO for 1 (isovalue ±0.05 (electron/bohr^3^)^1/2^), right: gas-phase electrophile affinity (Eα) values, determined using Br^+^ as test electrophile (B3LYP/6-311+G(2d,2p)). b) Calculated (B3LYP/6-311++G(2df,2p)//B3LYP/6-31G(d)) free energies for the deprotonation of each of the CH groups of 1 by acetate.

**Scheme 4:**
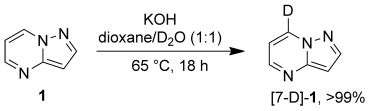
Deuteration of 1 to [7-D]-1, conversion determined by ^1^H NMR spectroscopy.

These observations suggest that the change in observed regioselectivity is the direct result of a switch in the C–H activation mechanism from electrophilic palladation in the case of C3 arylation under phosphine-free conditions, to a base-assisted deprotonation mechanism for C7 arylation on addition of SPhos.[21] In base-assisted deprotonation, the C–H bond cleavage is slow with respect to the coordination of the aryl function to the metal center, however, the rate-determining step in the catalytic manifold may not be the C–H bond cleavage. Indeed, a competition experiment between **1** and [7-D]-**1** (Scheme 5) shows only a modest kinetic isotope effect (KIE), inconsistent with C–H bond cleavage being the rate-determining step in the cycle.[22]

**Scheme 5:**
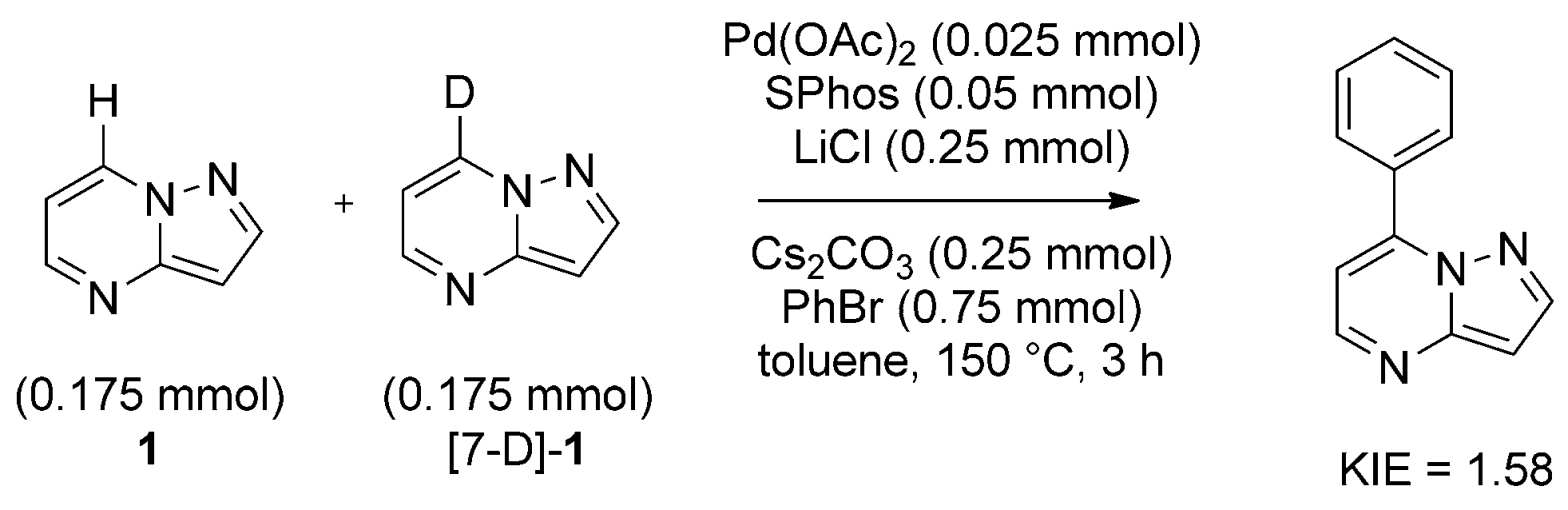
Competitive arylation of deuterated and nondeuterated substrates. KIE determined by ^1^H NMR spectroscopy (1,3,5-trimethoxybenzene used as internal standard).

With regard to the type of palladium species responsible for the C7 arylation, the requirement for SPhos makes it likely that the active component is a homogeneous complex. Indeed, a ^31^P{^1^H} NMR spectrum of a catalytic reaction mixture of **1** reacting with PhBr (conditions A, Scheme 2) recorded after 1.5 h (corresponding to ≈30 % conversion to **2 a**) showed the presence of two main species (in addition to free SPhos),[23] neither of which require the presence of either **1** or PhBr to form.[24] The structures and possible involvement or otherwise of these species in the catalytic cycle is the subject of ongoing investigations.

Turning our attention to the active species in C3 arylation, it seems likely that the reactions proceed through the formation of heterogeneous palladium. In contrast with the C7-arylation reaction, which showed a fairly short induction period of around 20 min,[25] the C3-arylation reaction displayed a protracted induction phase of several hours, a time that varied significantly between runs (Figure 5).[25, 26] In these cases, the timing of the onset of catalysis coincided with a change in appearance from a yellow, homogeneous solution to a black suspension.

**Figure 5:**
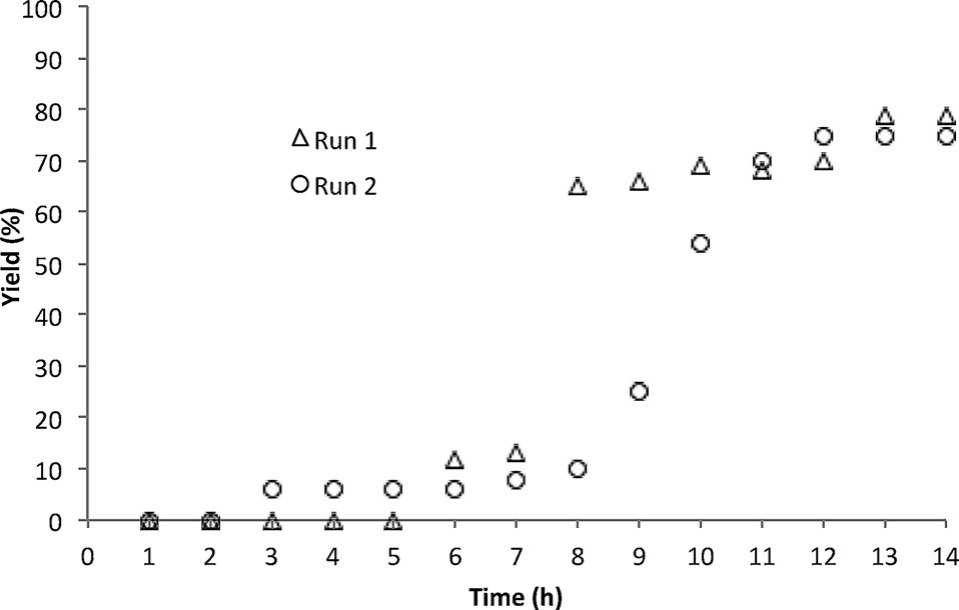
C3-Arylation of 1 with PhBr (see the Supporting Information for conditions) followed over time. Conversion to 3 a determined by ^1^H NMR spectroscopy (1,3,5-trimethoxybenzene used as internal standard).

The induction phase was followed by a period of activity before the onset of catalyst decomposition. Interestingly, there appear to be two distinct periods of catalysis (Figure 5), suggesting that there are at least two discrete catalytically active species, one formed during the induction phase before rapidly undergoing deactivation, and a second species that is formed later accounting for the major proportion of coupled product.[27] The extended induction period, the observation of more than one active species, and the fact that catalysis terminates before completion of the reaction all imply that the formation of heterogeneous palladium is not by itself sufficient for activity,[28] instead it is likely that catalysis is restricted to smaller palladium clusters or nanoparticles that change morphology over the course of the reaction before undergoing decomposition by over-aggregation, which leads to loss of activity.

In conclusion, we have developed a switchable site-selective direct arylation, wherein the switching is controlled by a change in mechanism, facilitated by tuning the composition of the catalyst. Thus the site of the reaction of pyrazolo[1,5-*a*]pyrimidine with aryl bromides can be switched between the remote positions C3 and C7. Studies are ongoing to both develop new reactions that proceed through switchable remote site-selective C–H functionalization and to fully elucidate the mechanisms that facilitate the switching.

## Supporting Information

As a service to our authors and readers, this journal provides supporting information supplied by the authors. Such materials are peer reviewed and may be re-organized for online delivery, but are not copy-edited or typeset. Technical support issues arising from supporting information (other than missing files) should be addressed to the authors.

miscellaneous_information
