# Supplementary material for: Catalyst-Switchable Regiocontrol in the Direct Arylation of Remote C–H Groups in Pyrazolo[1,5-a]pyrimidines
Source: Angew Chem Int Ed Engl. 2015 Jun 10;54(30):8787–90. doi: 10.1002/anie.201502150 (PMC4531820; doi:10.1002/anie.201502150)
Supplement: Supplementary file 1 — miscellaneous_information [file anie0054-8787-sd1.pdf]

Angewandte Chemie
Eine Zeitschrift der Gesellschaft Deutscher Chemiker

Supporting Information

Catalyst-Switchable Regiocontrol in the Direct Arylation of Remote C–H Groups in Pyrazolo[1,5-*a*]pyrimidines**

Robin B. Bedford, Steven J. Durrant, and Michelle Montgomery*

anie_201502150_sm_miscellaneous_information.pdf

Contents

1. General Conditions S2

2. Reaction Optimisation S2

3. General Procedures for Synthetic Arylation Reactions (Figures 2 and 3). S5

4. Reaction Selectivity S5

5. Product Characterisation S7

6. Reaction Profiles S19

7. Computation details S21

8. ^{1}H and ^{13}C NMR Spectra of Isolated Products S34

9. References S69

1. General Conditions

All reagents were purchased from commercial suppliers and used as received, without further purification. Pyrazolo[1,5-*a*]pyrimidine was purchased from Combi-Block Chemicals. Reactions were performed using anhydrous solvents under an atmosphere of air. NMR spectra were acquired at the indicated field strengths on either a Varian 500 MHz, 400 MHz, an ECP 400 MHZ or a Jeol ECP 300 MHz spectrometer. ^{1}H and ^{13}C NMR spectra were referenced to the residual *protio* solvent. ESI-MS were performed on a Daltonics Apex IV spectrometer. IR spectra were recorded on a Perkin Elmer Spectrum 100 FT-IR spectrometer with an ATR diamond cell.

2. Reaction Optimisation

Reaction optimisation was carried out in a glass-tube fitted with a Young's valve (nominal volume ~ 20 mL) under air. Pyrazolo[1,5-*a*]pyrimidine (0.25 mmol) and bromobenzene were combined with catalyst, ligand, base, additive, solvent and heated to the desired temperature for the required duration (see Tables S1-S3). The reaction mixture was filtered through Celite washing exhaustively with ethyl acetate and 1,3,5-trimethoxybenzene (internal standard, 0.25 mmol) was added to the washings. The solution was concentrated *in vacuo,* redissolved in $CDCl_3$ and analysed by ^{1}H NMR.

Table S1. Catalyst, Ligand, Base and Solvent Screen.[a]

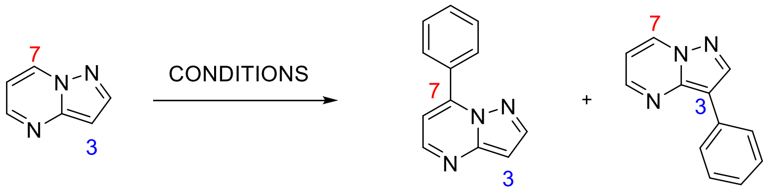

1 **2a** **3a**

Entry	Catalyst (10 mol%)	Ligand (20 mol%)	Base (Equiv)	Solvent/ Temp (°C)	Product, yield (%)[b]
1	$Pd(OAc)_2$	dppf	Cs_2CO_3 (1)	NMP/ 150	9% **2a**; 6% **3a**
2	$Pd(OAc)_2$	dppf	K_2CO_3 (1)	NMP/ 150	7% **2a**; 6% **3a**
3	$Pd(OAc)_2$	dppf	NaOAc (1)	NMP/ 150	No Rx
4	$Pd(OAc)_2$	dppf	CsOPiv (1)	NMP/ 150	6% **2a**; 0% **3a**
5	$Pd(OAc)_2$	dppf	LiO^{t}Bu (1)	NMP/ 150	5% **2a**; 0% **3a**
6	$Pd(OAc)_2$	dppf	Cs_2CO_3 (2)	NMP/ 150	10% **2a**; 6% **3a**
7	$Pd(OAc)_2$	dppf	Cs_2CO_3 (3)	NMP/ 150	9% **2a**; 6% **3a**
8	$Pd(OAc)_2$	P(*o*-tolyl)$_3$	Cs_2CO_3 (1)	NMP/ 150	4% **2a**; 4% **3a**
9	$Pd(OAc)_2$	PCy_3	Cs_2CO_3 (1)	NMP/ 150	7% **2a**; 4% **3a**
10	$Pd(OAc)_2$	PPh_3	Cs_2CO_3 (1)	NMP/ 150	0% **2a**; 7% **3a**
11	$Pd(OAc)_2$	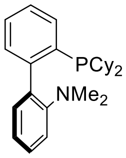	Cs_2CO_3 (1)	NMP/ 150	15% **2a**; 10% **3a**

12	$Pd(OAc)_2$	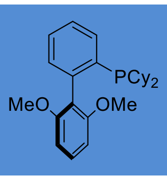	Cs_2CO_3 (1)	NMP/ 150	28% **2a**; 0% **3a**
13	$Pd(OAc)_2$	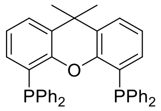	Cs_2CO_3 (1)	NMP/ 150	23% **2a**; 7% **3a**
14	$Pd(OAc)_2$	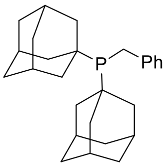	Cs_2CO_3 (1)	NMP/ 150	6% **2a**; 0% **3a**
15	$Pd(OAc)_2$	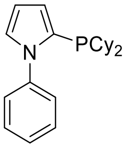	Cs_2CO_3 (1)	NMP/ 150	6% **2a**; 0% **3a**
16	$Pd(OAc)_2$	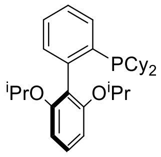	Cs_2CO_3 (1)	NMP/ 150	10% **2a**; 0% **3a**
17	$Pd(OAc)_2$	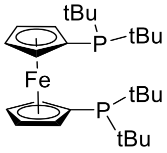	Cs_2CO_3 (1)	NMP/ 150	8% **2a**; 0% **3a**
18	$Pd(TFA)_2$	SPhos	Cs_2CO_3 (1)	NMP/ 150	16% **2a**; 0% **3a**
19	$Pd(dba)_2$	SPhos	Cs_2CO_3 (1)	NMP/ 150	10% **2a**; 0% **3a**
20	$PdCl_2$(dtbpf)	SPhos	Cs_2CO_3 (1)	NMP/ 150	11% **2a**; 0% **3a**
21	$Pd(PPh_3)_4$	SPhos	Cs_2CO_3 (1)	NMP/ 150	5% **2a**; 0% **3a**
22	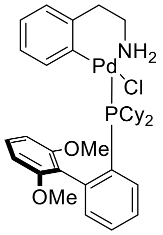	SPhos	Cs_2CO_3 (1)	NMP/ 150	6% **2a**; 0% **3a**
23	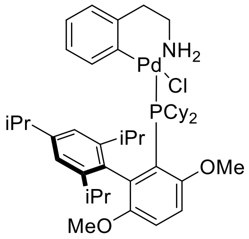	SPhos	Cs_2CO_3 (1)	NMP/ 150	8% **2a**; 0% **3a**

24	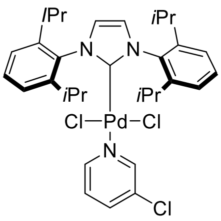	SPhos	Cs_2CO_3 (1)	NMP/ 150	No Reaction
25	$Pd(OAc)_2$	SPhos	Cs_2CO_3 (1)	NMP/ 100	No Reaction
26	$Pd(OAc)_2$	SPhos	Cs_2CO_3 (1)	Dioxane/ 100	12% **2a**; 0% **3a**
27	$Pd(OAc)_2$	SPhos	Cs_2CO_3 (1)	DMA/ 100	No Reaction
28	$Pd(OAc)_2$	SPhos	Cs_2CO_3 (1)	DCE/ 100	No Reaction
29	$Pd(OAc)_2$	SPhos	Cs_2CO_3 (1)	MeCN/ 100	10% **2a**; 0% **3a**
30	$Pd(OAc)_2$	SPhos	Cs_2CO_3 (1)	PhCl/ 100	15% **2a**; 0% **3a**

[a] Conditions: pyrazolo[1,5-*a*]pyrimidine (0.25 mmol), bromobenzene (0.25 mmol), catalyst (10 mol%), ligand (20 mol%), base (n equiv), sealed tube under air, 18h. [b] Spectroscopic yields determined by ^{1}H NMR using 1,3,5-trimethoxybenzene as an internal standard.

Table S2. Additives tested.[a]

Entry	Additive	Product, yield (%)[b]
1	AcOH	24% **2a**; 0% **3a**
2	TFA	21% **2a**; 0% **3a**
3	*p*TSA	12% **2a**; 0% **3a**
4	PivOH	20% **2a**; 0% **3a**
5	TFAA	13% **2a**; 0% **3a**
6	PEG	9% **2a**; 9% **3a**
7	TBAC	8% **2a**; 0% **3a**
8	LiCl	35% **2a**; 0% **3a**

[a] Conditions: pyrazolo[1,5-*a*]pyrimidine (0.25 mmol), bromobenzene (0.25 mmol), $Pd(OAc)_2$ (10 mol%), SPhos (20 mol%), Cs_2CO_3 (0.25 mmol), additive (0.25 mmol), NMP (0.5 ml), sealed tube under air, 150 °C, 18h. [b] Spectroscopic yields determined by ^{1}H NMR using 1,3,5-trimethoxybenzene as an internal standard.

Table S3. PhBr equivalent screen.[a]

Entry	Equivalents PhBr used	Product, yield (%)[b]
1	1	70% **2a**
2	2	76% **2a**
3	2.5	80% **2a**
4	3	84% **2a**
5	3.5	75% **2a**

[a] Conditions: pyrazolo[1,5-*a*]pyrimidine (0.25 mmol), bromobenzene, $Pd(OAc)_2$ (10 mol%), SPhos (20 mol%), Cs_2CO_3 (1 equiv), LiCl (1 equiv), toluene (0.5 ml), sealed tube under air, 150 °C, 18 h. [b] Spectroscopic yields determined by ^{1}H NMR using 1,3,5-trimethoxybenzene as an internal standard.

3. General Procedures for Synthetic Arylation Reactions (Figures 2 and 3).

General procedure for C7-arylation of 1 (conditions A). To a glass-tube fitted with a Young's valve (nominal volume ~ 20 mL), was added **1** (29.8 mg, 0.25 mmol), Cs_2CO_3 (81.5 mg, 0.25 mmol,), SPhos (20.5 mg, 0.05 mmol), $Pd(OAc)_2$ (5.6 mg, 0.025 mmol) LiCl (10.6 mg, 0.25 mmol), the appropriate aryl bromide (0.75 mmol) and anhydrous toluene (0.5 mL). The tube was sealed and the resultant mixture was stirred at 150 °C for 18 h. The mixture was cooled to room temperature, diluted with ethyl acetate (~ 10 mL), filtered through Celite and concentrated *in vacuo*. The crude material was purified by flash chromatography eluting with 0-70% ethyl acetate in petroleum ether. Isolated yields are given in Figure 2, selectivities are given in Table S4.

General procedure for the C3-arylation of 1 (conditions B). As above with **1** (29.8 mg, 0.25 mmol,), K_2CO_3 (81.5 mg, 0.25 mmol), $Pd(OAc)_2$ (5.6 mg, 0.025 mmol), LiCl (10.6 mg, 0.25 mmol), the appropriate aryl bromide (0.25 mmol) and anhydrous 1,4-dioxane (0.5 mL) and a reaction temperature of 120 °C. Isolated yields are given in Figure 3, selectivities are given in Table S4.

Larger Scale Examples. The procedures above were repeated on a larger scale (1.0 mmol of **1**) using bromobenzene. **Conditions A** gave **2a** in 79% isolated yield, while **conditions b** afforded **2b** in 70% spectroscopic yield.

4. Reaction Selectivity

Table S4 shows the selectivities for the reactions summarised in Figures 2 and 3, as determined by ^{1}H NMR spectroscopy. The diarylated products **4** were not isolated, but there presence or otherwise was confirmed by UHPLC-MS analysis.

Table S4. C3:C7 selectivity obtained under conditions A and conditions B with each ArBr[a]

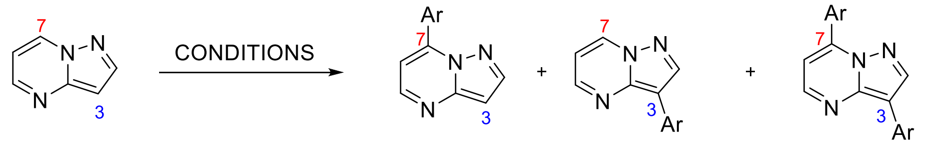

1 **2** **3** **4**

ArBr	Conditions A[a]	Conditions B[a]
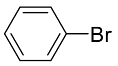	**2a**, 86%; **3a**, 0%;<br>**4a**, 4% [[M+H]$^+$ = 272.1][c]	**2a**, 1%; **3a**, 75%;<br>**4a**, 7% [[M+H]$^+$ = 272.1][c]
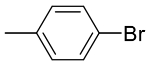	**2b**, 84%; **3b**, 0%;<br>**4c**, 0%	**2b**, 6%; **3b**, 57%;<br>**4c**, 1% [[M+H]$^+$ = 300.1][c]

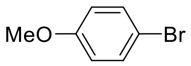	**2c**, 82%; **3c**, 0%<br>**4d**, 4% [[M+H]$^+$ = 332.1][c]	**2c**, 0%; **3c**, 33%;<br>**4d**, 7% [[M+H]$^+$ = 332.1][c]
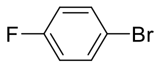	**2d**, 86%; **3d**, 0%;<br>**4e**, 4% [[M+H]$^+$ = 308.1][c]	**2d**, 0%; **3d**, 66%;<br>**4e**, 5% [[M+H]$^+$ = 308.1][c]
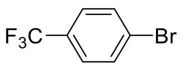	**2e**, 86%; **3e**, 0%;<br>**4f**, 5% [[M+H]$^+$ = 408.1][c]	**2e**, 0%; **3e**, 86%;<br>**4f**, 0%
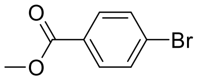	**2f**, 90%; **3f**, 0%;<br>**4g**, 0%	**2f**, 1%; **3f**, 74%;<br>**4g**, 4%; [[M+H]$^+$ = 388.1][c]
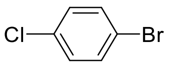	**2g**, 92%; **3g**, 0%;<br>**4h**, 4% [[M+H]$^+$ = 340.1][c]	**2g**, 3%; **3g**, 78%;<br>**4h**, 5% [[M+H]$^+$ = 340.1][c]
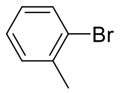	**2h**, 82%; **3h**, 0%;<br>**4i**, 1% [[M+H]$^+$ = 300.1][c]	**2h**, 0%; **3h**, 60%;<br>**4i**, 5% [[M+H]$^+$ = 300.1][c]
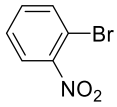	**2i**, 55%; **3i**, 0%;<br>**4j,** 0%	**2i**, 0%; **3i**, 75%<br>**4j**, 0%
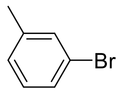	**2j**, 85%; **3i**, 0%;<br>**4k**, 7% [[M+H]$^+$ = 300.1][c]	**2j**, 1%; **3i**, 69%;<br>**4k**, 6% [[M+H]$^+$ = 300.1][c]
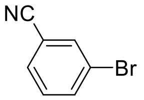	No Reaction	**3k**, 71%;<br>**4l**, 6% [[M+H]$^+$ = 322.1][c]
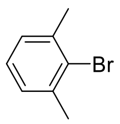	**2k**, 50%; **3l**, 0%;<br>**4m**, 5% [[M+H]$^+$ = 328.2][c]	**2k**, 0%; **3l**, 27%;<br>**4m**, 3% [[M+H]$^+$ = 328.2][c]
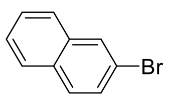	**2l**, 52%; **3m**, 0%;<br>**4n**, 5% [[M+H]$^+$ = 372.2][c]	**2l**, 0%; **3m**, 53%;<br>**4n**, 7% [[M+H]$^+$ = 372.1][c]
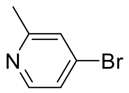	**2m**, 77%; **3n**, 0%;<br>**4o**, 7% [[M+H]$^+$ = 302.1][c]	**2m**, 1%; **3n**, 58%;<br>**4o**, 6% [[M+H]$^+$ = 302.1][c]

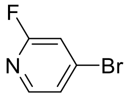	**2n**, 38%; **3o**, 0%; **4p**, 5% [[M+H]$^+$ = 310.1][c]	**2n**, 0%; **3o**, 45%; **4p**, 4% [[M+H]$^+$ = 310.1][c]
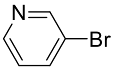	**2o**, 71%; **3p**, 0%; **4q**, 6% [[M+H]$^+$ = 274.1][c]	**2o**, 0%; **3p**, 72%; **4q**, 7% [[M+H]$^+$ = 274.1][c]
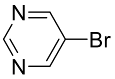	**2p**, 55%; **3q**, 0% **4r**, 4% [[M+H]$^+$ = 276.1][c]	**2p**, 1%; **3q**, 64%; **4r**, 7% [[M+H]$^+$ = 276.1][c]

[a] As reported in Scheme 2. [b] Spectroscopic yields determined by ^{1}H NMR (1,3,5-trimethoxybenzene as internal standard); isolated yields of **2** and **3** are given in Figures 2 and 3. [c] As determined by UHPLC-MS (ES).

5. Product Characterisation

The pyrazolo[1,5-*a*]pyrimidine core is numbered as follows:

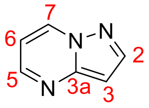

7-phenylpyrazolo[1,5-a]pyrimidine, 2a (Figure 2)

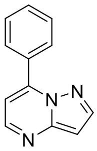

2a

Prepared according to conditions A (Scheme 2) to afford the product as a yellow solid, 35 mg (72%); ^{1}H NMR (500 MHz, $CDCl_3$) δ 8.52 (d, *J* = 4.3 Hz, 1H, C5*H*), 8.17 (d, *J* = 2.3 Hz, 1H, C2*H*), 8.05 - 8.00 (m, 2H), 7.58 - 7.54 (m, 3H), 6.89 (d, *J* = 4.3 Hz, 1H, C6*H*), 6.79 (d, *J* = 2.3 Hz, 1H, C3*H*); ^{13}C NMR (126 MHz, $CDCl_3$) δ 149.90 (*C3a*), 148.97 (*C5*H), 146.81 (*C7*), 144.69 (*C2*H), 131.10 (*C*), 131.30 (*C*H), 129.20 (*C*H), 128.69 (*C*H), 107.27 (*C6*H), 97.08 (*C3*H); IR (neat), ν (cm^{-1}) 3103, 3070, 3042, 2920, 1538, 764; m.p. 71.6 – 72.4. °C; HRMS (ESI) calcd for $C_{12}H_{10}N_3$ $(M+H)^+$ 196.0869, found 196.0866.

7-(p-tolyl)pyrazolo[1,5-a]pyrimidine, 2b (Figure 2)

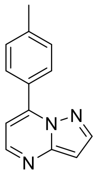

2b

Prepared according to conditions A (Scheme 2) to afford a yellow solid, 37 mg (70%); ^{1}H NMR (500 MHz, $CDCl_3$) δ 8.51 (d, *J* = 4.3 Hz, 1H, C5*H*), 8.16 (d, *J* = 2.3 Hz, 1H, C2*H*), 7.94 (d, *J* = 8.1 Hz, 2H), 7.37 (d, *J* = 8.1 Hz, 2H), 6.88 (d, *J* = 4.3 Hz, 1H, C6*H*), 6.77 (d, *J* = 2.3 Hz, 1H, C3*H*), 2.45 (s, 3H); ^{13}C NMR (126 MHz, $CDCl_3$) δ 149.92 (*C3a*), 148.94 (*C5*H), 146.94 (*C7*), 144.60 (*C2*H), 141.51 (*C*), 129.38 (*C*H), 129.14 (*C*H), 128.19 (*C*), 106.92 (*C6*H), 96.93 (*C3*H), 21.56 (*C*H_3). IR (neat), ν (cm^{-1}) 3103, 3051, 3032, 2920, 1541, 808. m.p. 92.4 – 93.7 °C; HRMS (ESI) calcd for $C_{13}H_{11}N_3$ $(M+H)^+$ 210.1026, found, 210.1028.

7-(4-methoxyphenyl)pyrazolo[1,5-a]pyrimidine, 2c (Figure 2)

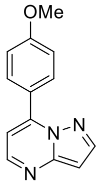

2c

Prepared according to conditions A (Scheme 2) to afford an orange gum, 39 mg (69%); ^{1}H NMR (500 MHz, $CDCl_3$) δ 8.49 (d, *J* = 4.4 Hz, 1H, C5*H*), 8.16 (d, *J* = 2.3 Hz, 1H, C2*H*), 8.07 (d, *J* = 8.4 Hz, 2H), 7.07 (d, *J* = 8.4 Hz, 2H), 6.87 (d, *J* = 4.4 Hz, 1H, C6*H*), 6.76 (d, *J* = 2.3 Hz, 1H, C3*H*), 3.89 (s, 3H); ^{13}C NMR (126 MHz, $CDCl_3$) δ 160.79 (*C*OCH_3), 148.91 (*C3a*), 147.82 (*C5*H), 145.62 (*C7*), 143.55 (*C2*H), 129.94 (*C*H), 122.19 (*C*), 113.11 (*C*H), 105.44 (*C6*H), 95.81 (*C3*H), 54.46 (O*C*H_3); IR (DCM), ν (cm^{-1}) 3055, 2986, 2898, 1543, 1251; HRMS (ESI) calcd for $C_{13}H_{11}N_3O$ $(M+H)^+$ 226.0975, found 226.0972.

7-(4-fluorophenyl)pyrazolo[1,5-a]pyrimidine, 2d (Figure 2)

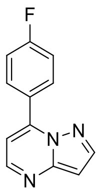

2d

Prepared according to conditions A (Scheme 2) to afford a yellow solid, 37 mg (69%); ^{1}H NMR (500 MHz, $CDCl_3$) δ 8.53 (d, *J* = 4.3 Hz, 1H, C5*H*), 8.17 (d, *J* = 2.4 Hz, 1H, C2*H*), 8.11 – 8.06 (m, 2H), 7.29 – 7.23 (m, 2H), 6.88 (d, *J* = 4.3 Hz, 1H, C6*H*), 6.79 (d, *J* = 2.4 Hz, 1H, C3*H*); ^{13}C NMR (126 MHz, $CDCl_3$) δ 164.22 (d, *J* = 250.8 Hz, *C*F), 149.84 (*C3a*), 148.88 (*C5*H), 145.78 (*C7*), 144.74 (*C2*H), 131.50 (d, *J* = 8.6 Hz, (*C*H), 127.10 (d, *J* = 3.3, *C*) 115.91 (d, *J* = 22.6, *C*H), 107.05 (*C6*H), 97.21 (*C3*H); ^{19}F NMR (471 MHz, $CDCl_3$) δ -108.15; IR (neat), ν (cm^{-1}) 3065, 3044, 3012, 2964, 1505, 764; m.p. 130.1 – 131.7 °C; HRMS (ESI) calcd for $C_{12}H_8FN_3$ $(M+H)^+$ 214.0775, found 214.0771.

7-(4-(trifluoromethyl)phenyl)pyrazolo[1,5-a]pyrimidine, 2e (Figure 2)

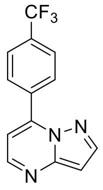

2e

Prepared according to conditions A (Scheme 2) to afford a yellow gum, 49 mg (75%); ^{1}H NMR (500 MHz, $CDCl_3$) δ 8.56 (d, *J* = 4.3 Hz, 1H, C5*H*), 8.19 – 8.14 (m, 3H), 7.83 (d, *J* = 8.2 Hz, 2H), 6.92 (d, *J* = 4.3 Hz, 1H, C6*H*), 6.82 (d, *J* = 2.4 Hz, 1H, C3*H*); ^{13}C NMR (126 MHz, $CDCl_3$) δ 149.82 (*C3a*), 148.91 (*C5*H), 145.16 (*C7*), 144.88 (*C2*H), 134.49 (*C*), 132.76 (q, *J* = 33.1, *C*CF_3), 129.68 (*C*H), 125.69 (q, *J* = 3.6 Hz, *C*H), 123.66 (q, *J* = 268.2 Hz, *C*F_3), 107.56 (*C6*H), 97.54 (*C3*H); ^{19}F NMR (471 MHz, $CDCl_3$) δ -63.09; IR (neat), ν (cm^{-1}) 3067, 3045, 2934, 2854, 1319, 817; HRMS (ESI) calcd for $C_{13}H_9F_3N_3$ $(M+H)^+$ 264.0743, found 264.0742.

Methyl 4-(pyrazolo[1,5-a]pyrimidin-7-yl)benzoate, 2f (Figure 2)

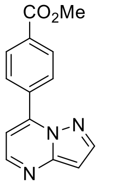

2f

Prepared according to conditions A (Scheme 2) to afford a yellow solid, 55 mg (88%); ^{1}H NMR (500 MHz, $CDCl_3$) δ 8.55 (d, *J* = 4.2 Hz, 1H, C5*H*), 8.22 (d, *J* = 8.3 Hz, 2H), 8.17 (d, *J* = 2.4 Hz, 1H, C2*H*), 8.14 - 8.10 (m, 2H), 6.93 (d, *J* = 4.2 Hz, 1H, C6*H*), 6.81 (d, *J* = 2.4 Hz, 1H, C3*H*), 3.96 (s, 3H); ^{13}C NMR (126 MHz, $CDCl_3$) δ 166.22 (C*C*(O)OMe), 149.85 (*C3a*), 148.87 (*C5*H), 145.59 (*C7*), 144.82 (*C2*H), 135.19 (*C*), 132.24 (*C*), 129.82 (*C*H), 129.27 (*C*H), 107.57 (*C6*H), 97.40 (*C3*H), 52.43 (C(O)O*C*H_3); IR (neat), ν (cm^{-1}) 3052, 3012, 2956, 1719, 1255, 761; m.p. 163.4 – 164.6 °C; HRMS (ESI) calcd for $C_{14}H_{12}N_3O_2$ $(M+H)^+$ 254.0924, found 254.0922.

7-(4-chlorophenyl)pyrazolo[1,5-a]pyrimidine, 2g (Figure 2)

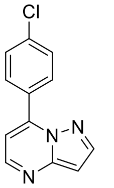

2g

Prepared according to conditions A (Scheme 2) to afford a yellow solid, 49 mg (85%); ^{1}H NMR (500 MHz, $CDCl_3$) δ 8.52 (d, *J* = 4.3 Hz, 1H, C5*H*), 8.16 (d, *J* = 2.4 Hz, 1H, C2*H*), 8.01 (d, *J* = 8.5 Hz, 2H), 7.54 (d, *J* = 8.5 Hz, 2H), 6.88 (d, *J* = 4.3 Hz, 1H, C6*H*), 6.79 (d, *J* = 2.4 Hz, 1H, C3*H*); ^{13}C NMR (126 MHz, $CDCl_3$) δ 149.82 (*C3a*), 148.85 (*C5*H), 145.61 (*C*), 144.76 (*C2*H), 137.27 (*C*), 130.58 (*C*H), 129.39 (*C*), 129.01 (*C*H), 107.09 (*C6*H), 97.28 (*C3*H); IR (neat), ν (cm^{-1}) 3045, 3028, 2965, 1606, 722; m.p. 143.5 – 144.3 °C; HRMS (ESI) calcd for $C_{12}H_8ClN_3$ $(M+H)^+$ 230.0480, found 230.0477.

7-(o-tolyl)pyrazolo[1,5-a]pyrimidine, 2h (Figure 2)

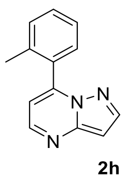

2h

Prepared according to conditions A (Scheme 2) to afford an orange gum, 37 mg (71%); ^{1}H NMR (500 MHz, $CDCl_3$) δ 8.54 (d, *J* = 4.1 Hz, 1H, C5*H*), 8.13 (d, *J* = 2.3 Hz, 1H, C2*H*), 7.47 (td, *J* = 7.4, 1.7 Hz, 1H), 7.41 - 7.33 (m, 3H), 6.78 (d, *J* = 2.3 Hz, 1H, C3*H*), 6.76 (d, *J* = 4.1 Hz, 1H, C6*H*), 2.15 (s, 3H, *CH₃*); ^{13}C NMR (126 MHz, $CDCl_3$) δ 149.26 (*C3a*), 148.73 (*C5*H), 147.75 (*C7*), 145.02 (*C2*H), 137.14 (*C*), 131.31 (*C*), 130.58 (*C*H), 130.47 (*C*H), 129.25 (*C*H), 126.11 (*C*H), 108.52 (*C6*H), 97.01 (*C3*H), 19.60 (CH_3); IR (neat), ν (cm^{-1}) 3141, 3070, 3019, 2924, 2869, 1538, 780; HRMS (ESI) calcd for $C_{13}H_{11}N_3$ $(M+H)^+$ 210.1026, found 210.1030.

7-(2-nitrophenyl)pyrazolo[1,5-a]pyrimidine, 2i (Figure 2)

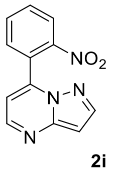

2i

Prepared according to conditions A (Scheme 2) to afford a yellow solid, 29 mg (48%); ^{1}H NMR (500 MHz, $CDCl_3$) δ 8.59 (d, *J* = 4.2 Hz, 1H, C5*H*), 8.30 (dd, *J* = 8.1, 1.3 Hz, 1H), 8.03 (d, *J* = 2.3 Hz, 1H, C2*H*), 7.84 (td, *J* = 7.5, 1.3 Hz, 1H), 7.77 (dd, *J* = 8.1, 1.5 Hz, 1H), 7.63 (dd, *J* = 7.5, 1.5 Hz, 1H), 6.89 (d, *J* = 4.2 Hz, 1H, C6*H*), 6.77 (d, *J* = 2.3 Hz, 1H, C3*H*); ^{13}C NMR (126 MHz, $CDCl_3$) δ 148.90 (*C5*H), 148.83 (*C3a*), 144.91 (*C2*H), 144.71 (*C7*), 134.08 (*C*H), 131.93 (*C*H), 131.68 (*C*H), 126.43 (*C*), 125.09 (*C*H), 107.18 (*C6*H), 97.51 (*C3*H); IR (neat), ν (cm^{-1}) 3114, 3096, 2924, 1599, 1350, 784; m.p. 180.7 – 181.4 °C; HRMS (ESI) calcd for $C_{12}H_8N_4O_2(M+H)^+$ 241.0720, found 241.0723.

7-(m-tolyl)pyrazolo[1,5-a]pyrimidine, 2j (Figure 2)

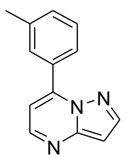

2j

Prepared according to conditions A (Scheme 2) to afford a yellow gum, 39 mg (75%); ^{1}H NMR (500 MHz, $CDCl_3$) δ 8.51 (d, *J* = 4.3 Hz, 1H, C5*H*), 8.16 (d, *J* = 2.4 Hz, 1H, C2*H*), 7.82 – 7.78 (m, 2H), 7.45 (t, *J* = 7.4 Hz, 1H), 7.37 (d, *J* = 6.2 Hz, 1H), 6.86 (d, *J* = 4.3 Hz, 1H, C6*H*), 6.78 (d, *J* = 2.4 Hz, 1H, C3*H*), 2.46 (s, 3H, C*H*₃); ^{13}C NMR (126 MHz, $CDCl_3$) δ 149.89 (*C3a*), 148.95 (*C5*H), 147.06 (*C7*), 144.66 (*C2*H), 138.46 (*C*), 131.82 (*C*), 131.05 (*C*H), 129.67 (*C*H), 128.60 (*C*H), 126.33 (*C*H), 107.29 (*C6*H), 96.99 (*C3*H), 21.54 (CH_3); IR (neat), ν (cm^{-1}) 3147, 3063, 2920, 2848, 1580, 773; HRMS (ESI) cacld for $C_{13}H_{11}N_3$ $(M+H)^+$ 210.1026, found 210.1028.

7-(2,6-dimethylphenyl)pyrazolo[1,5-a]pyrimidine, 2k (Figure 2)

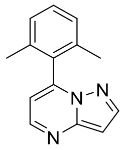

2k

Prepared according to conditions A (Scheme 2) to afford a orange solid, 28mg (50%); ^{1}H NMR (500 MHz, $CDCl_3$) δ 8.56 (d, *J* = 4.1 Hz, 1H, C5*H*), 8.11 (d, *J* = 2.4 Hz, 1H, C2*H*), 7.35 (t, *J* = 7.7 Hz, 1H), 7.20 (d, *J* = 7.7 Hz, 2H), 6.78 (d, *J* = 2.4 Hz, 1H, C3*H*), 6.73 (d, *J* = 4.1 Hz, 1H, C6*H*), 2.04 (s, 6H); ^{13}C NMR

(126 MHz, $CDCl_3$) δ 148.28 (*C3a*), 147.72 (*C5*H), 145.78 (*C7*), 144.16 (*C2*H), 135.40 (*C*), 129.93 (*C*H), 129.02 (*C*), 126.79 (*C*H), 107.73 (*C6*H), 95.90 (*C3*H), 18.53 (*C*H_3); IR (neat), ν $(cm)^{-1}$ 3115, 3103, 2964, 1538, 781; m.p. 112.5 – 114.3 °C; HRMS (ESI) calcd for $C_{14}H_{13}N_3$ $(M+H)^+$ 224.1182, found 224.1185.

7-(naphthalen-2-yl)pyrazolo[1,5-a]pyrimidine, 2l (Figure 2)

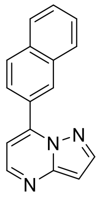

2l

Prepared according to conditions A (Scheme 2) to afford a yellow solid, 32 mg (52%); ^{1}H NMR (500 MHz, $CDCl_3$) δ 8.61 (d, *J* = 4.1 Hz, 1H, C5*H*), 8.09 (d, *J* = 2.3 Hz, 1H, C2*H*), 8.07 (d, *J* = 8.3 Hz, 1H), 7.96 (d, *J* = 8.4 Hz, 1H), 7.71 (d, *J* = 7.1 Hz, 1H), 7.63 (d, *J* = 8.4 Hz, 1H), 7.54 (ddd, *J* = 8.2, 6.7, 1.3 Hz, 1H), 7.43 (ddd, *J* = 8.1, 6.7, 1.3 Hz, 1H), 7.37 (d, *J* = 8.5 Hz, 1H), 6.93 (d, *J* = 4.1 Hz, 1H, C6*H*), 6.84 (d, *J* = 2.3 Hz, 1H, C3*H*); ^{13}C NMR (126 MHz, $CDCl_3$) δ 149.54 (*C3a*), 148.77 (*C5*H), 146.66 (*C7*), 145.00 (*C2*H), 133.59 (*C*), 131.12 (*C*H), 130.53 (*C*), 129.12 (*C*), 128.75 (*C*H), 127.83 (*C*H), 127.08 (*C*H), 126.58 (*C*H), 125.23 (*C*H), 124.93 (*C*H), 109.58 (*C6*H), 97.15 (*C3*H); IR (neat), ν (cm^{-1}) 3127, 3056, 2987, 2923, 2851, 1606, 1538, 736; m.p. 130.3 – 131.5 °C; HRMS (ESI) calcd for $C_{16}H_{11}N_3$ $(M+H)^+$ 246.1026, found 246.1033.

7-(2-methylpyridin-4-yl)pyrazolo[1,5-a]pyrimidine, 2m (Figure 2)

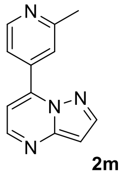

2m

Prepared according to conditions A (Scheme 2) to afford a yellow solid, 32 mg (62%); ^{1}H NMR (500 MHz, $CCDl_3$) δ 8.73 (dd, *J* = 5.3, 0.9 Hz, 1H), 8.56 (d, *J* = 4.2 Hz, 1H, C5*H*), 8.19 (d, *J* = 2.4 Hz, 1H, C2*H*), 7.83 (s, 1H), 7.74 (d, *J* = 5.3 Hz, 1H), 6.94 (d, *J* = 4.2 Hz, 1H, C6*H*), 6.83 (d, *J* = 2.4 Hz, 1H, C3*H*), 2.70 (s, 3H, CH_3); ^{13}C NMR (126 MHz, $CDCl_3$) δ 159.29 (*C*H), 149.79 (*C3a*), 149.54 (*C*H), 148.82 (*C5*H), 144.94 (*C3*H), 144.14 (*C7*), 139.08 (*C*H), 122.65 (*C*), 120.21 (*C*), 107.56 (*C6*H), 97.66 (*C3*H), 24.58 (*C*H_3); IR (neat), ν (cm^{-1}) 3049, 3001, 2984, 2918, 1596, 767; m.p. 126.1 – 126.8 °C; HRMS (ESI) calcd for $C_{12}H_{11}N_4$ $(M+H)^+$ 211.0978, found 211.0985.

7-(2-fluoropyridin-4-yl)pyrazolo[1,5-a]pyrimidine, 2n (Figure 2)

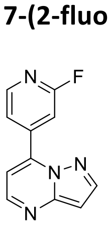

2n

Prepared according to conditions A (Scheme 2) to afford a white solid, 15mg (27%); ^{1}H NMR (500 MHz, $CDCl_3$) δ 8.60 (d, *J* = 4.2 Hz, 1H, C5*H*), 8.46 (dd, *J* = 5.1, 0.8 Hz, 1H), 8.21 (d, *J* = 2.4 Hz, 1H, C2*H*),

7.86 (dt, *J* = 5.2, 1.5 Hz, 1H), 7.72 (t, *J* = 1.5 Hz, 1H), 7.00 (d, *J* = 4.2 Hz, 1H, C6*H*), 6.87 (d, *J* = 2.4 Hz, 1H, C3*H*); ^{13}C NMR (126 MHz, $CDCl_3$) δ 163.47 (d, *J* = 237.7 Hz, *C*F), 149.76 (*C3a*), 148.68 (*C5*H), 148.49 (d, *J* = 15.9 Hz, *C*H), 145.10 (*C3*H), 143.53 (d, *J* = 10.6 Hz, *C*H), 120.83 (d, *J* = 4.43 Hz, *C*H), 109.85 (d, *J*= 42.73 Hz, *C*) 107.64 (*C6*H), 97.99 (*C3*H). ^{19}F NMR (471 MHz, $CDCl_3$) δ -65.65. IR (neat), ν (cm^{-1}) 3127, 3096, 3057, 2975, 2956, 1542, 802, 785; m.p. 174.1 – 175.2 °C; HRMS (ESI) calcd for $C_{11}H_7FN_4$ $(M+H)^+$ 215.0728, found 215.0720.

7-(pyridin-3-yl)pyrazolo[1,5-a]pyrimidine, 2o (Figure 2)

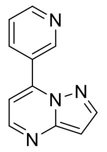

2o

Prepared according to conditions A (Scheme 2) to afford a yellow gum, 29 mg (59%); ^{1}H NMR (500 MHz, $CDCl_3$) δ 9.18 (s, 1H), 8.82 – 8.77 (m, 1H), 8.59 – 8.54 (m, 2H), 8.17 (d, *J* = 2.4 Hz, 1H, C2*H*), 7.53 (ddd, *J* = 8.2, 4.9, 0.9 Hz, 1H), 6.95 (d, *J* = 4.2 Hz, 1H, C6*H*), 6.82 (d, *J* = 2.4 Hz, 1H, C3*H*); ^{13}C NMR (126 MHz, $CDCl_3$) δ 150.62 (*C*H), 148.76 (*C3a*), 148.48 (*C5*H), 147.87 (*C3*H), 143.83 (*C*H), 142.59 (*C7*), 135.92 (*C*H), 126.39 (*C*), 122.23 (*C*H), 106.26 (*C6*H), 96.59 (*C3*H); IR (neat), ν (cm^{-1}) 3096, 3043, 2962, 2920, 1609, 765; m.p. 143.0 – 143.8 °C; HRMS (ESI) calcd for $C_{11}H_9N_4$ $(M+H)^+$ 197.0822, found 197.0823.

7-(pyrimidin-5-yl)pyrazolo[1,5-a]pyrimidine, 2p (Figure 2)

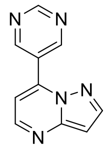

2p

Prepared according to conditions A (Scheme 2) to afford a red solid (18 mg, 37%). ^{1}H NMR (500 MHz, $CDCl_3$) δ 9.49 (s, 2H), 9.39 (s, 1H), 8.60 (d, *J* = 4.2 Hz, 1H), 8.20 (d, *J* = 2.4 Hz, 1H), 6.99 (d, *J* = 4.2 Hz, 1H), 6.86 (d, *J* = 2.3 Hz, 1H). ^{13}C NMR (126 MHz, $CDCl_3$) δ 160.00, 156.85, 149.59, 148.71, 145.10, 140.75, 125.80, 107.16, 98.04. IR (neat), ν (cm^{-1}) 3109, 3097, 3081, 3057, 2965, 2874, 785, 714; m.p. decom 228 °C; HRMS (ESI) calcd for $C_{10}H_7N_5$ $(M+H)^+$ 198.0774, found 198.0767.

3-phenylpyrazolo[1,5-a]pyrimidine, 3a (Figure 3)

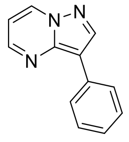

3a

Prepared according to conditions B (Scheme 2) to afford a yellow solid, 36 mg (73%); ^{1}H NMR (500 MHz, $CDCl_3$) δ 8.69 (d, *J* = 7.0 Hz, 1H, C7*H*), 8.56 (dd, *J* = 4.0, 1.8 Hz, 1H, C5*H*), 8.45 (s, 1H, C2*H*), 8.04 (d, *J* = 8.4 Hz, 2H), 7.46 (t, *J* = 8.4 Hz, 2H), 7.28 (t, *J* = 8.4 Hz, 1H), 6.84 (dd, *J* = 7.0, 4.0 Hz, 1H, C6*H*); ^{13}C NMR (126 MHz, $CDCl_3$) δ 149.17 (*C5*H), 144.87 (*C3a*), 142.86 (*C2*H), 135.32 (*C7*H), 131.81 (*C*), 128.81 (*C*H), 126.45 (*C*H), 126.41 (*C*H), 110.95 (*C3*), 108.03 (*C6*H); IR (neat), ν (cm^{-1}) 3052, 3028,

2872, 2901, 1615, 763; m.p. 72.3 – 72.8 °C; HRMS (ESI) calcd for $C_{12}H_9N_3$ $(M+H)^+$ 196.0869, found 196.0866.

3-(p-tolyl)pyrazolo[1,5-a]pyrimidine, 3b (Figure 3)

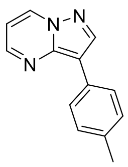

3b

Prepared according to conditions B (Scheme 2) to afford a yellow solid, 28 mg (55%); ^{1}H NMR (500 MHz, $CDCl_3$) δ 8.59 (dd, *J* = 7.0, 1.8 Hz, 1H, C7*H*), 8.46 (dd, *J* = 4.0, 1.8 Hz, 1H, C5*H*), 8.34 (s, 1H, C2*H*), 7.84 (d, *J* = 8.3 Hz, 2H), 7.19 (t, *J* = 7.7 Hz, 2H), 6.74 (dd, *J* = 7.0, 4.0 Hz, 1H, C6*H*), 2.31 (s, 3H); ^{13}C NMR (126 MHz, $CDCl_3$) δ 148.94 (*C5*H), 144.72 (*C3a*), 142.68 (*C2*H), 136.13 (*C*), 135.24 (*C7*H), 129.49 (*C*H), 128.81 (*C*), 126.33 (*C*H), 111.01 (*C3*), 107.90 (*C6*H), 21.23 (*C*H_3); IR (neat), ν (cm^{-1}) 3061, 3004, 2972, 2917, 1616, 1404, 784; m.p. 121.7 – 122.5 °C; HRMS (ESI) calcd for $C_{13}H_{11}N_3$ $(M+H)^+$ 210.1026, found 210.1024.

3-(4-methoxyphenyl)pyrazolo[1,5-a]pyrimidine, 3c (Figure 3)

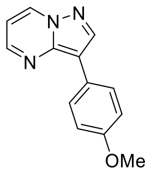

3c

Prepared according to conditions B (Scheme 2) to afford a yellow solid, 19 mg (33%); ^{1}H NMR (500 MHz, $CDCl_3$) δ 8.67 (dd, *J* = 7.1, 1.8 Hz, 1H, C7*H*), 8.53 (dd, *J* = 4.0, 1.8 Hz, 1H, C5*H*), 8.38 (s, 1H, C2*H*), 7.95 (d, *J* = 8.3 Hz, 2H), 7.01 (d, *J* = 8.3 Hz, 2H), 6.82 (dd, *J* = 7.1, 4.0 Hz, 1H, C6*H*), 3.85 (s, 3H, OCH_3); ^{13}C NMR (126 MHz, $CDCl_3$) δ 157.35 (*C*OMe), 147.78 (*C5*H), 143.50 (*C3a*), 141.42 (*C2*H), 134.20 (*C7*H), 126.63 (*C*H), 123.41 (*C*), 113.31 (*C*H), 109.81 (*C6*H), 106.82 (*C3*), 54.33 (O*C*H_3); IR (neat), ν (cm^{-1}) 3048, 3008, 2898, 1609, 1251. m.p. 114.4 – 115.3 °C; HRMS (ESI) calcd for $C_{13}H_{11}N_3O$ $(M+H)^+$ 226.0975, found 226.0976.

3-(4-fluorophenyl)pyrazolo[1,5-a]pyrimidine, 3d (Figure 3)

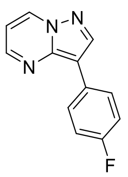

3d

Prepared according to conditions B (Scheme 2) to afford a yellow solid, 35 mg (66%); ^{1}H NMR (500 MHz, $CDCl_3$) δ 8.69 (dd, *J* = 7.0, 1.8 Hz, 1H, C7*H*), 8.56 (dd, *J* = 4.0, 1.8 Hz, 1H, C5*H*), 8.39 (s, 1H, C2*H*), 8.05 – 7.97 (m, 2H), 7.18 – 7.11 (m, 2H), 6.85 (dd, *J* = 7.0, 4.0 Hz, 1H, C5*H*); ^{13}C NMR (126 MHz, $CDCl_3$) δ 161.66 (d, *J* = 256.1 Hz, *C*F), 149.21 (*C5*H), 144.70 (*C3a*), 142.60 (*C2*H), 135.33 (*C7*H), 127.95 (d, *J* = 8.1 Hz, *C*H), 115.70 (d, *J* = 22.05 Hz, *C*H), 110.09 (*C6*H), 108.09 (*C3*), 99.98 (*C*); ^{19}F NMR (471 MHz,

CDCl$_3$) δ -115.95; IR (neat), ν (cm^{-1}) 3063, 3001, 2985, 1618, 775; m.p. 142.6 – 143.3 °C; HRMS (ESI) calcd for $C_{12}H_9FN_3$ (M+H) 214.0775, found 214.0770.

3-(4-(trifluoromethyl)phenyl)pyrazolo[1,5-a]pyrimidine, 3e (Figure 3)

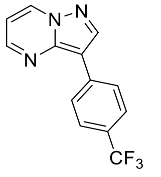

3e

Prepared according to conditions B (Scheme 2) to afford a yellow solid, 53 mg (80%); ^{1}H NMR (500 MHz, CDCl$_3$) δ 8.73 (dd, *J* = 7.1, 1.8 Hz, 1H, C7*H*), 8.61 (dd, *J* = 4.0, 1.8 Hz, 1H, C5*H*), 8.49 (s, 1H, C2*H*), 8.18 (d, *J* = 8.5 Hz, 2H), 7.70 (d, *J* = 8.5 Hz, 2H), 6.91 (dd, *J* = 7.1, 4.0 Hz, 1H, C6*H*); ^{13}C NMR (126 MHz, CDCl$_3$) δ 149.78, 145.22 (*C5*H), 143.03 (*C3a*), 135.53 (*C2*H), 128.53 (*C7*H), 128.0 (q, *J* = 231.7 Hz, *C*F$_3$), 126.42 (*C*H), 126.21 (*C*), 125.68 (q, *J* = 3.76 Hz, *C*H), 123.29 (*C*), 109.47 (*C*3), 108.47 (*C6*H); ^{19}F NMR (471 MHz, CDCl$_3$) δ -62.40; IR (neat), ν (cm^{-1}) 3076, 3057, 2992, 2903, 1615, 1065, 780; m.p. 150.2 – 151.7 °C. HRMS (ESI) calcd for $C_{13}H_9F_3N_3$ 264.0743, found 264.0742.

Methyl 4-(pyrazolo[1,5-a]pyrimidin-3-yl)benzoate, 3f (Figure 3)

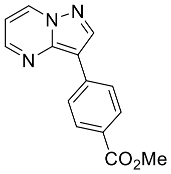

3f

Prepared according to conditions B (Scheme 2) to afford an orange solid, 43 mg (68%); ^{1}H NMR (500 MHz, CDCl$_3$) δ 8.70 (dd, *J* = 7.0, 1.8 Hz, 1H, C7*H*), 8.60 (dd, *J* = 4.0, 1.8 Hz, 1H, C5*H*), 8.50 (s, 1H, C2*H*), 8.14 (d, *J* = 9.1 Hz, 2H), 8.10 (d, *J* = 9.5 Hz, 1H), 6.88 (dd, *J* = 7.0, 4.0 Hz, 1H, C6*H*), 3.93 (s, 3H, C*H*$_3$); ^{13}C NMR (126 MHz, CDCl$_3$) δ 167.06 (C*C*(O)OMe), 149.76 (*C5*H), 145.21 (*C3a*), 143.18 (*C2*H), 136.58 (*C*), 135.52 (*C7*H), 130.13 (*C*H), 127.64 (*C*), 125.75 (*C*H), 109.75 (*C3*), 108.44 (*C6*H), 52.02 (C(O)O*C*H$_3$); IR (neat), ν (cm^{-1}) 3107, 3003, 2957, 2887, 1701, 1280, 771; m.p. 180.7 – 181.6 °C; HRMS (ESI) calcd for $C_{14}H_{12}N_3O_2$ $(M+H)^+$ 254.0924, found 254.0921.

3-(4-chlorophenyl)pyrazolo[1,5-a]pyrimidine, 3g (Table 3, entry 10)

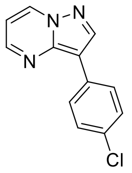

3g

Prepared according to conditions B (Scheme 2) to afford a yellow solid, 45 mg (78%);^{1}H NMR (500 MHz, CDCl$_3$) δ 8.70 (d, *J* = 7.0 Hz, 1H, C7*H*), 8.57 (dd, *J* = 3.9, 1.8 Hz, 1H, C5*H*), 8.42 (s, 1H, C2*H*), 7.99 (d, *J* = 9.1 Hz, 2H), 7.42 (d, *J* = 8.6 Hz, 2H), 6.87 (dd, *J* = 7.0, 3.9 Hz, 1H, C6*H*); ^{13}C NMR (126 MHz, CDCl$_3$) δ 149.34 (*C5*H), 144.83 (*C3a*), 142.69 (*C2*H), 135.39 (*C7*H), 132.01 (*C*), 130.35 (*C*), 128.91 (*C*H),

127.49 (*C*H), 109.76 (*C3*), 108.19 (*C6*H); IR (neat), ν (cm^{-1}) 3065, 1611, 1398, 773; m.p. decomp. 175.0 °C; HRMS (ESI) calcd for $C_{12}H_8ClN_3$ $(M+H)^+$ 230.0480, found 230.0478.

3-(o-tolyl)pyrazolo[1,5-a]pyrimidine, 3h (Figure 2)

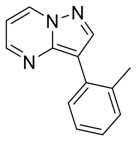

3h

Prepared according to conditions B (Scheme 2) to afford a yellow solid, 28 mg (54%); ^{1}H NMR (400 MHz, $CDCl_3$) δ 8.70 (dd, *J* = 7.0, 1.5 Hz, 1H, C7*H*), 8.50 (dd, *J* = 4.0, 1.5 Hz, 1H, C5*H*), 8.21 (s, 1H, C3), 7.52 (d, *J* = 8.7 Hz, 1H), 7.35 – 7.21 (m, 3H), 6.84 (dd, *J* = 7.0, 4.0 Hz, 1H, C6*H*), 2.37 (s, 3H); ^{13}C NMR (101 MHz, $CDCl_3$) δ 149.05 (*C5*H), 145.25 (*C3a*), 144.83 (*C2*H), 136.72 (*C*), 135.18 (*C7*H), 130.67 (*C*), 130.64 (*C5*H), 130.55 (*C*H), 127.42 (*C*H), 125.87 (*C*H), 111.43 (*C3*), 107.90 (*C6*H), 20.82 (*C*H_3); IR (neat), ν (cm^{-1}) 3048, 2958, 2923, 1617, 766; m.p. 103.9 – 104.8 °C; HRMS (ESI) calcd for $C_{13}H_{12}N_3$ $(M+H)^+$ 210.1026, found 210.1030.

3-(2-nitrophenyl)pyrazolo[1,5-a]pyrimidine, 3i (Figure 3)

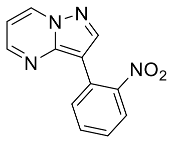

3i

Prepared according to conditions B (Scheme 2) to afford a yellow solid, 43 mg (71%); ^{1}H NMR (500 MHz, $CDCl_3$) δ 8.67 (dd, *J* = 7.0, 1.8 Hz, 1H, C7*H*), 8.55 (dd, *J* = 4.0, 1.8 Hz, 1H, C5*H*), 8.40 (s, 1H, C2*H*), 8.00 – 7.94 (m, 2H), 7.42 – 7.37 (m, 2H), 6.84 (dd, *J* = 7.0, 4.0 Hz, 1H, C6*H*); ^{13}C NMR (126 MHz, $CDCl_3$) δ 149.33 (*C5*H), 144.80 (*C3a*), 142.64 (*C2*H), 135.37 (*C7*H), 131.98 (*C*), 130.35 (*C*), 128.89 (*C*H), 127.47 (*C*H), 109.72 (*C3*), 108.18 (*C6*H); IR (neat), ν (cm^{-1}) 3057, 3049, 2999, 2961, 1519, 1362, 780; m.p. 135.2 – 136.7 °C; HRMS (ESI) calcd for $C_{12}H_9N_4O_2$ $(M+H)^+$ 241.0720, found 241.0716.

3-(m-tolyl)pyrazolo[1,5-a]pyrimidine, 3j (Figure 3)

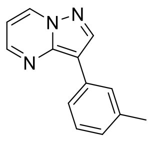

3j

Prepared according to conditions B (Scheme 2) to afford a yellow gum, 29mg (55%); ^{1}H NMR (500 MHz, $CDCl_3$) δ 8.67 (dd, *J* = 7.1, 1.8 Hz, 1H, C7*H*), 8.55 (dd, *J* = 4.0, 1.8 Hz, 1H, C5*H*), 8.43 (s, 1H, C2*H*), 7.86 - 7.82 (m, 2H), 7.35 (d, *J* = 7.3 Hz, 1H), 7.10 (d, *J* = 7.5 Hz, 1H), 6.82 (dd, *J* = 7.1, 4.0 Hz, 1H, C6*H*), 2.44 (s, 3H); ^{13}C NMR (126 MHz, $CDCl_3$) δ 148.03 (*C5*H), 143.80 (*C3a*), 141.92 (*C2*H), 137.30 (*C*), 134.25 (*C7*H), 130.66 (*C*), 127.68 (*C*H), 126.25 (*C*H), 126.06 (*C*H), 122.54 (*C*H), 109.99 (*C3*), 107.64 (*C6*H), 20.60 (*C*H_3); IR (neat), ν (cm^{-1}) 3068, 3029, 2917, 2859, 1609, 776; HRMS (ESI) calcd for $C_{13}H_{12}N_3$ $(M+H)^+$ 210.1025, found 210.1018.

3-(pyrazolo[1,5-a]pyrimidin-3-yl)benzonitrile, 3k (Figure 3)

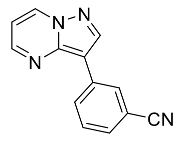

3k

Prepared according to conditions B (Scheme 2) to afford a yellow solid, 39 mg (70%); ^{1}H NMR (500 MHz, $CDCl_3$) δ 8.72 (dd, *J* = 7.0, 1.8 Hz, 1H, C7*H*), 8.61 (dd, *J* = 4.0, 1.8 Hz, 1H, C6*H*), 8.47 – 8.43 (m, 2H), 8.25 (td, *J* = 4.6, 1.8 Hz, 1H), 7.53 (d, *J* = 5.2 Hz, 2H), 6.93 (dd, *J* = 7.0, 4.0 Hz, 1H, C6*H*); ^{13}C NMR (126 MHz, $CDCl_3$) δ 148.88 (*C5*H), 144.08 (*C3a*), 141.65 (*C2*H), 134.53 (*C7*H), 132.26 (*C*), 129.05 (*C*H), 128.61 (*C*H), 128.52 (*C*H), 128.46 (*C*H), 118.06 (*CN*), 111.92 (*C*), 107.60 (*C6*H), 107.52 (*C3*); IR (neat), ν (cm^{-1}) 3115, 3096, 2972, 2226, 1615, 1394, 790; m.p. decomp. 203 °C; HRMS (ESI) calcd for $C_{13}H_8N_4$ $(M+H)^+$ 221.0822, found 221.0817.

3-(2,6-dimethylphenyl)pyrazolo[1,5-a]pyrimidine, 3l (Figure 3)

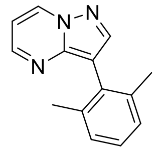

3l

Prepared according to conditions B (Scheme 2) to afford a yellow solid, 12mg (23%); ^{1}H NMR (400 MHz, $CDCl_3$) δ 8.72 (dd, *J* = 7.0, 1.8 Hz, 1H, C7*H*), 8.46 (dd, *J* = 3.9, 1.8 Hz, 1H, C5*H*), 8.07 (s, 1H, C2*H*), 7.22 – 7.12 (m, 3H), 6.83 (dd, *J* = 7.0, 4.0 Hz, 1H, C6*H*), 2.11 (s, 6H, 2 x C*H*$_3$); ^{13}C NMR (101 MHz, CDCl3) δ 148.92 (*C5*H), 145.16 (*C3a*), 145.12 (*C2*H), 138.21 (*C*H), 135.18 (*C7*H), 130.01 (*C*), 127.81 (*C*H), 127.52 (*C*H), 109.88 (*C3*), 107.67 (*C6*H), 20.96 (2 x *C*H$_3$); IR (neat), ν (cm^{-1}) 3154, 2984, 2918, 1615, 1517, 761; m.p. 121.0 – 121.6 °C; HRMS (ESI) calcd for $C_{14}H_{13}N_3$ 224.1182, found 224.1186

3-(naphthalen-2-yl)pyrazolo[1,5-a]pyrimidine, 3m (Figure 3)

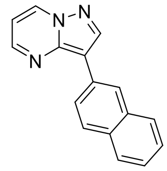

3m

Prepared according to conditions B (Scheme 2) to afford an orange gum, 26 mg (42%); ^{1}H NMR (500 MHz, $CDCl_3$) δ 8.79 (d, *J* = 7.1 Hz, 1H, C7*H*), 8.53 (dd, *J* = 4.0, 1.8 Hz, 1H, C5*H*), 8.40 (s, 1H, C2*H*), 8.02 (d, *J* = 7.6 Hz, 1H), 7.90 (dd, *J* = 17.0, 8.3 Hz, 2H), 7.71 (d, *J* = 7.1 Hz, 1H), 7.58 (d, *J* = 8.2 Hz, 1H), 7.49 (ddt, *J* = 22.6, 8.2, 6.8, 1.4 Hz, 2H), 6.88 (dd, *J* = 7.1, 4.0 Hz, 1H, C6*H*); ^{13}C NMR (126 MHz, $CDCl_3$) δ 149.46 (*C5*H), 145.90 (*C3a*), 145.61 (*C2*H), 135.33 (*C7*H), 134.11 (*C*), 132.09 (*C*), 128.92 (*C*), 128.49 (*C*H), 128.24 (*C*H), 127.91 (*C*H), 126.12 (*C*H), 125.84 (*C*H), 125.68 (*C*H), 125.60 (*C*H), 110.38 (*C3*), 108.19 (*C6*H); IR (neat), ν (cm^{-1}) 3126, 3063, 2851, 1606, 1608, 776; HRMS (ESI) calcd for $C_{16}H_{11}N_3$ 246.1026, found 246.1020.

3-(2-methylpyridin-4-yl)pyrazolo[1,5-a]pyrimidine, 3n (Figure 3)

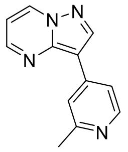

3n

Prepared according to conditions B (Scheme 2) to afford a yellow solid, 30 mg (57%); ^{1}H NMR (500 MHz, CDCl$_3$) δ 8.66 (dd, *J* = 7.0, 1.8 Hz, 1H, C7*H*), 8.57 (dd, *J* = 4.0, 1.8 Hz, 1H, C5*H*), 8.47 - 8.44 (m, 2H), 7.80 (s, 1H, C2*H*), 7.72 (dd, *J* = 5.4, 1.7 Hz, 1H), 6.86 (dd, *J* = 7.0, 4.0 Hz, 1H, C6*H*), 2.56 (s, 3H, C*H*$_3$); ^{13}C NMR (126 MHz, CDCl$_3$) δ 157.60 (*C*), 149.09 (*C*H), 148.33 (*C5*H), 144.63 (*C*3a), 142.27 (*C2*H), 138.85 (*C*), 134.63 (*C7*H), 118.75 (*C*H), 116.58 (*C*H), 107.66 (*C6*H), 107.11 (*C*3), 23.55 (*C*H$_3$); IR (neat), ν (cm^{-1}) 3084, 3061, 3019, 2922, 1604, 1298, 758. m.p. 194.1 – 195.4 °C; HRMS (ESI) calcd for $C_{12}H_{10}N_4$ (M+H)$^+$ 211.0978, found 211.0988.

3-(2-fluoropyridin-4-yl)pyrazolo[1,5-a]pyrimidine, 3o (Figure 3)

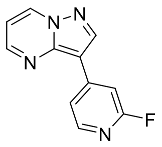

3o

Prepared according to conditions B (Scheme 2) to afford a white solid, 8 mg (15%); ^{1}H NMR (500 MHz, CDCl$_3$) δ 8.68 (dd, *J* = 7.0, 1.8 Hz, 1H, C7*H*), 8.61 (dd, *J* = 4.0, 1.7 Hz, 1H, C5*H*), 8.47 (s, 1H, C2*H*), 8.17 (d, *J* = 5.3 Hz, 1H), 7.80 – 7.77 (m, 1H), 7.66 (s, 1H), 6.92 (dd, *J* = 7.0, 4.1 Hz, 1H, C6*H*); ^{13}C NMR (126 MHz, CDCl$_3$) δ 164.66 (d, *J* = 239.64 Hz, *C*), 150.51 (*C5*H), 147.80 (d, *J* = 17.25 Hz, *C*H), 145.84 (*C3a*), 144.82 (d, *J* = 9.6 Hz, *C*), 143.35 (*C2*H), 135.78 (*C7*H), 117.80 (d, *J* = 4.06 Hz, *C*H), 109.04 (*C6*H), 107.16 (*C3*), 102.49 (d, *J* = 40.3 Hz, *C*H); ^{19}F NMR (471 MHz, DMSO) δ -69.08; IR (neat), ν (cm^{-1}) 3115, 3061, 2903, 1612, 1399, 761; m.p. 230.5 – 231.7 °C; HRMS (ESI) calcd for $C_{11}H_8FN_4$ (M+H)$^+$ 215.0727, found 215.0729.

3-(pyridin-3-yl)pyrazolo[1,5-a]pyrimidine, 3p (Figure 3)

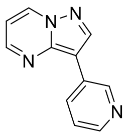

3p

Prepared according to conditions B (Scheme 2) to afford a yellow gum (34 mg, 68%). ^{1}H NMR (500 MHz, CDCl$_3$) δ 9.22 (dd, *J* = 2.4, 0.9 Hz, 1H), 8.71 (dd, *J* = 7.1, 1.8 Hz, 1H), 8.59 (dd, *J* = 4.0, 1.8 Hz, 1H), 8.51 (dd, *J* = 4.8, 1.6 Hz, 1H), 8.48 (s, 1H), 8.41 (dt, *J* = 8.0, 2.0 Hz, 1H), 7.37 (ddd, *J* = 8.0, 4.8, 0.9 Hz, 1H), 6.90 (dd, *J* = 7.0, 4.0 Hz, 1H). ^{13}C NMR (126 MHz, CDCl$_3$) δ 148.70, 146.26, 146.21, 144.24, 141.49, 134.46, 132.46, 127.10, 122.63, 107.45, 106.51; IR (neat), ν (cm^{-1}) 3104, 3057, 2928, 1609, 773; HRMS (ESI) calcd for $C_{11}H_8N_4$ (M+H)$^+$ 197.0822, found 197.0829.

3-(pyrimidin-5-yl)pyrazolo[1,5-a]pyrimidine, 3q (Figure 3)

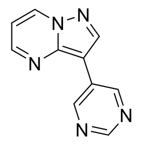

3q

Prepared according to conditions B (Scheme 2) to afford a yellow solid, 29 mg (59%); ^{1}H NMR (500 MHz, $CDCl_3$) δ 9.41 (s, 2H), 9.11 (s, 1H), 8.75 (dd, *J* = 7.1, 1.8 Hz, 1H, C7*H*), 8.64 (dd, *J* = 4.0, 1.8 Hz, 1H C5*H*), 8.51 (s, 1H, C2*H*), 6.96 (dd, *J* = 7.1, 4.0 Hz, 1H, C6*H*); ^{13}C NMR (126 MHz, $CDCl_3$) δ 155.10 (*C*H), 152.71 (*C*H), 149.34 (*C5*H), 144.45 (*C3a*), 141.21 (*C2*H), 134.61 (*C7*H), 125.55 (*C*), 107.91 (*C3*), 103.17 (*C6*H); IR (neat), ν (cm^{-1}) 3061, 2957, 2918, 1612, 1398, 774; m.p. 231.0 – 231.7 °C; HRMS (ESI) calcd for $C_{10}H_7N_5$ $(M+H)^+$ 198.0774, found 198.0776.

pyrazolo[1,5-a]pyrimidine-7-d, D7-1 (Scheme 4)

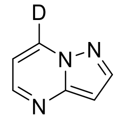

D7-1

A round bottomed flask was charged with pyrazolo[1,5-*a*]pyrimidine (30 mg, 0.25 mmol), potassium hydroxide (90 mg, 1.6 mmol), dioxane (1 ml) and D_2O (1 ml). The resulting solution was stirred at 65 °C for 18 h. The reaction was extracted into ether (~ 10 ml), dried over sodium carbonate and concentrated *in vacuo* to afford **D7-1** in quantitative yield; ^{1}H NMR (400 MHz, $CDCl_3$) δ 8.46 (d, *J* = 4.1 Hz, 1H, C5*H*), 8.12 (d, *J* = 2.4 Hz, 1H, C2*H*), 6.77 (d, *J* = 4.1 Hz, 1H, C6*H*), 6.69 (d, *J* = 2.4 Hz, 1H, C3*H*).

3-(2-methylpyridin-4-yl)-7-phenylpyrazolo[1,5-a]pyrimidine, 4a

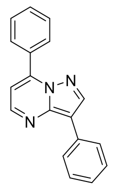

4a

Prepared according to conditions B (Scheme 2) starting from **3a** to afford a yellow solid, 48 mg (75%); ^{1}H NMR (500 MHz, $CDCl_3$) δ 8.62 (d, *J* = 4.2 Hz, 1H, C6*H*), 8.48 (s, 1H, C2*H*), 8.08 (d, *J* = 7.3 Hz, 2H,), 8.06 – 8.02 (m, 2H), 7.61 – 7.57 (m, 3H), 7.48 (d, *J* = 7.3 Hz, 2H), 7.30 (t, *J* = 7.3 Hz, 1H), 6.93 (d, *J* = 4.2 Hz, 1H, C5*H*); ^{13}C NMR (126 MHz, $CDCl_3$) δ 148.05 (*C5*H), 145.99 (*C3a*), 145.07 (*C7*), 141.58 (*C2*H), 131.05 (*C*), 130.07 (*C*H), 130.01 (*C*), 128.20 (*C*H), 127.76 (*C*H), 127.71 (*C*H), 125.52 (*C*H), 125.33 (*C*H), 109.86 (*C*3), 106.65 (*C6*H); IR (neat), ν (cm^{-1}) 3058, 2973, 2927, 1556, 758, 692; m.p. 167.3 – 168.1 °C; HRMS (ESI) calcd for $C_{18}H_{13}N_3$ $(M+H)^+$ 272.1182, found 272.1179.

3-phenyl-7-(4-(trifluoromethyl)phenyl)pyrazolo[1,5-a]pyrimidine, 4b

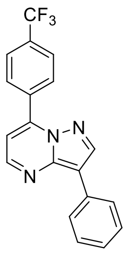

4b

Prepared according to conditions A (Scheme 2) from **2a** to afford a yellow solid, 45 mg (54%); ^{1}H NMR (400 MHz, $CDCl_3$) δ 8.65 (d, J = 4.2 Hz, 1H, C6*H*), 8.47 (s, 1H, C2*H*), 8.17 (d, J = 8.1 Hz, 2H), 8.08 – 8.03 (m, 2H), 7.85 (d, J = 8.1 Hz, 2H), 7.48 (t, J = 7.7 Hz, 2H), 7.30 (t, J = 7.5 Hz, 1H), 6.95 (d, J = 4.2 Hz, 1H, C5*H*); ^{13}C NMR (126 MHz, $CDCl_3$) δ 149.01 (*C*5H), 145.99 (*C*3a), 145.38 (*C*7), 142.77 (*C*2H), 133.55 (q, *J* = 218.29 Hz), 131.75 (C) 129.71 (*C*H), 128.84 (*C*H), 127.64 (q, *J* = 127.6 Hz, CF_3) 126.60 (*C*H), 126.59 (*C*H), 125.76 (q, *J* = 3.6 Hz, *C*H), 111.39 (*C*3*)*, 107.95 (*C*6H); ^{19}F NMR (470 MHz, $CDCl_3$) δ -63.09; IR (neat), ν (cm^{-1}) 3073, 2968, 2927, 1556, 1319, 814, 769, 691; m.p. 184.5 – 185.0 °C; HRMS (ESI) calcd for $C_{19}H_{13}F_3N_3$ $(M+H)^+$ 340.1056, found 340.1045.

6. Reaction Profiles

Procedure for monitoring the C7-arylation of 1: pyrazolo[1,5-a]pyrimidine (119 mg, 1 mmol), cesium carbonate (325 mg, 1 mmol), SPhos (80 mg, 0.20 mmol), palladium acetate (22 mg, 0.1 mmol), lithium chloride (40 mg, 1 mmol), bromobenzene (316 µl, 3 mmol), 1,3,5-trimethoxybenzene (168 mg, 1 mmol, internal standard) and anhydrous toluene (2 mL) were placed in a glass-tube fitted with a Young's valve (nominal volume ~ 20 mL) under air. The tube was sealed and heated to 150 °C. Aliquots of the reaction mixture were taken every 15 minutes for the first hour and then every hour afterwards for 16 hours. (CARE! Pressurised tube, with superheated solvent. Ensure sufficient headspace). Each aliquot was filtered through Celite, washing with ethyl acetate before being concentrated *in vacuo* and analysed by ^{1}H NMR. The results are summarised in Figure S1.

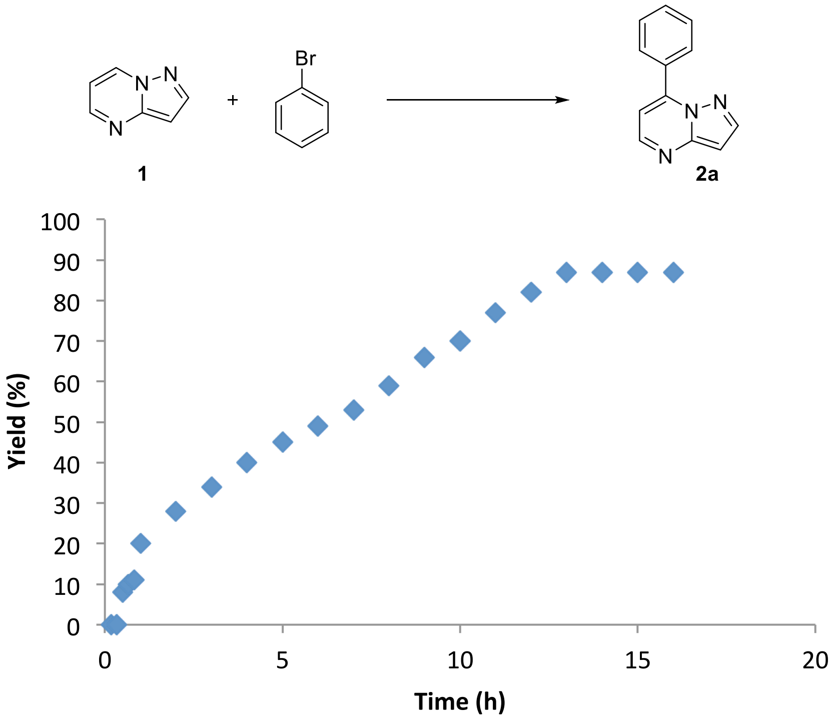

Figure S1. C7-Arylation Time Profile

Procedure for monitoring the C3-arylation of 1: pyrazolo[1,5-a]pyrimidine (29.8 mg, 0.25 mmol), potassium carbonate (34.5 mg, 0.25 mmol), palladium acetate (5.6 mg, 0.025 mmol), lithium chloride (10.6 mg, 0.25 mmol), bromobenzene (79 µl, 0.25 mmol), 1,3,5-trimethoxybenzene (42 mg, 0.25 mmol, internal standard) and anhydrous dioxane (0.5 mL) were added to a glass-tube fitted with a Young's valve (nominal volume ~ 20 mL)under air. 16 identical reactions were set up. The tubes were sealed and heated to 120 °C. One reaction was stopped every hour for 16 hours and filtered through Celite, washing with ethyl acetate, before being concentrated *in vacuo* and analysed by ^{1}H NMR. The results are summarised in Figure S2.

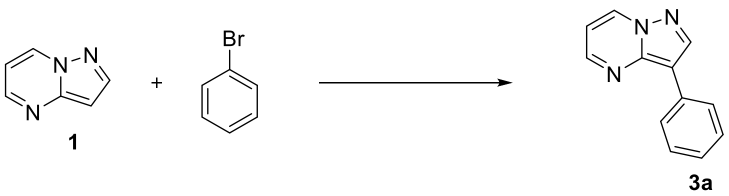

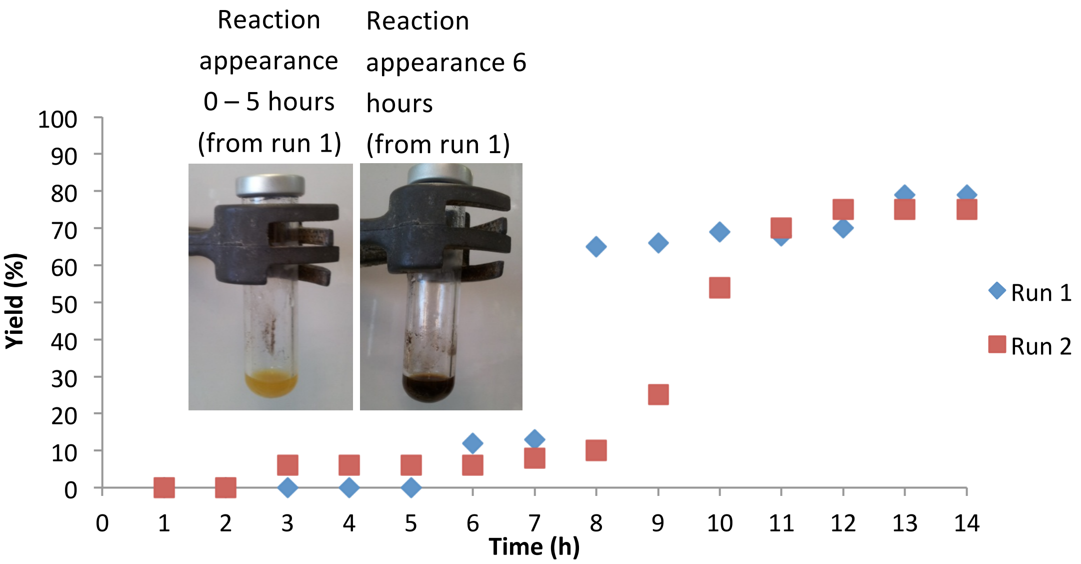

Figure S2. Reaction profile for C3 arylation.

7. Computation details

All DFT[S1] calculations were performed with Guassian 09 (rev. D).[S2] Geometry optimization of **1** was carried at the B3LYP-D2 level of theory,[S3,S4] using the standard 6-311++G(d) basis set.[S5] The HOMO of **1** was plotted (with an isovalue of ± 0.05 (electron/bohr^3)$^{1/2}$) using a single-point calculation at the previously optimised geometry. The methods for determining relative acidities and electrophile affinities are detailed below.

Relative acidities

The relative acidities for each of the C-H bonds of **1** was calculated by considering the deprotonation of **1** by the acetate ion, according to equation S1:

$$\mathbf{1} + Ac^- \xrightleftharpoons{\text{toluene}} (\mathbf{1} - H)^- + AcH \qquad (S1)$$

The relative acidities were expressed as the free energies of the exchange processes, as defined in equation S2:

$$\Delta G_{exch} = [\Delta G_{sol}(\mathbf{1} - H)^- + \Delta G_{sol}(AcH)] - [\Delta G_{sol}(\mathbf{1}) + \Delta G_{sol}(Ac^-)] \qquad (S2)$$

For each species, the structure was optimized and a frequency calculation was performed at the B3LYP level of theory, using the 6-31G(d) basis set and the Cartesian coordinates for these species and electronic energies are given below. All calculated structures showed no imaginary frequencies,

showing that they are intermediates. The Gibbs free energy for each species (ΔG_{sol} in Table S5) was determined by (a) running a single point calculation at the B3LYP level of theory, based on the results of the prior optimizations with the smaller basis set, using the 6-311++G(2df,2p) basis set with solvent (toluene) effects included using the PCM model (E_{sol}, Table S5),[S6] and then (b) adding the thermal correction obtained from the calculation from the smaller basis set.[S7] These values were then substituted into equation S2 to give the values for the exchange energies, summarized in Figure S3.

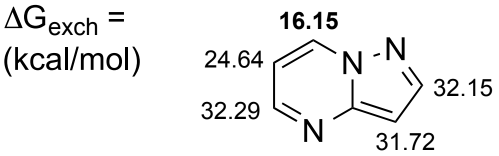

Figure S3. Exchange energies (as defined in Eqs. S1 and S2) for the various C-H residues of **1**.

Table S5. Electronic energies, thermal correction and free energies.

Compound and identifier	Electronic energy in toluene, E_{sol},[a] (a.u.)	Thermal correction to Gibbs free energy, TCG,[b] (a.u.)	ΔG_{sol} = E_{sol} + TCG (a.u.)
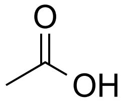 **AcH**	-229.1805617	0.009853	-229.1707087
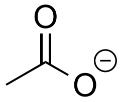 **Ac⁻**	-228.6724763	0.020689	-228.6517873
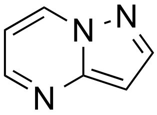 **1**	-396.0018770	0.076348	-395.925529
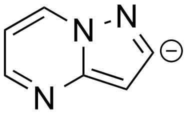 **[1-2an]⁻**	-395.4157591	0.060391	-395.3553681
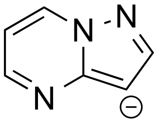 **[1-3an]⁻**	-395.4172374	0.061176	-395.3560614
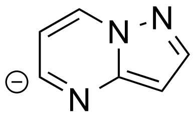 **[1-5an]⁻**	-395.4157848	0.06064	-395.3551448

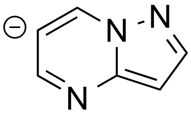 [1-6an]⁻	-395.4289636	0.061628	-395.3673356
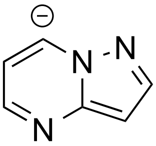 [1-7an]⁻	-395.4427458	0.061883	-395.3808628

[a] PCM/B3LYP/6-311++G(2df,2p)//B3LYP/6-31G(d). [b] B3LYP/6-31G(d)

Optimized Cartesian Coordinates of Computed (B3LYP/6-31G(d)) Structures

Compound: AcH

SCF energy: -229.0812035 a.u.

O	-0.82324	-1.02241	0.00000
C	-0.09007	0.11951	-0.00001
O	-0.59726	1.21868	0.00000
C	1.39442	-0.15753	0.00000
H	1.61245	-1.22639	-0.00000
H	1.84168	0.31200	0.88156
H	1.84169	0.31201	-0.88154
H	-1.75784	-0.73970	0.00001

Compound: Ac^-

SCF energy: -228.49791243 a.u.

O	0.80886	-1.10831	0.00001
C	0.22014	0.00173	-0.00002
O	0.69594	1.16608	0.00001
C	-1.35430	-0.05516	0.00000
H	-1.73303	-1.08537	0.00001
H	-1.75023	0.47189	-0.88060
H	-1.75021	0.47189	0.88062

Compound: 1

SCF energy: -395.87029004 a.u.

C	0.88245	-1.37183	0.00001
C	-0.25566	0.75661	0.00004
C	2.01885	0.74380	-0.00030
H	0.77388	-2.44994	0.00025
H	2.94926	1.30878	-0.00043
C	-1.59675	1.13307	0.00003
H	-1.97969	2.14174	-0.00014
N	-0.27179	-0.65466	0.00018
N	-1.52064	-1.16607	0.00095
C	-2.30644	-0.07559	-0.00045
H	-3.38002	-0.21473	-0.00076
C	2.06350	-0.68021	-0.00022
H	3.00921	-1.20889	-0.00022
N	0.90552	1.44758	-0.00018

Compound: [1-2an]$^{-}$

SCF energy: -395.21175204 a.u.

N	-0.31377	0.66621	0.00000
C	0.83305	1.36370	0.00000
C	2.03691	0.68455	0.00000
C	1.99109	-0.73460	0.00000
N	0.87660	-1.44666	-0.00000
C	-0.30341	-0.76547	0.00000
C	-1.64205	-1.10719	0.00000
C	-2.46840	0.08383	-0.00000
N	-1.60828	1.15455	0.00000

H	2.98100	1.22091	0.00000
H	2.92673	-1.29990	0.00000
H	0.72508	2.44527	0.00000
H	-1.99779	-2.13385	0.00000

Compound: [1-3an]⁻

SCF energy: -395.21499548 a.u.

N	0.30888	0.65459	-0.00000
C	-0.83163	1.37658	0.00000
C	-2.02775	0.69783	0.00000
C	-1.99347	-0.72681	0.00001
N	-0.89050	-1.45059	0.00000
C	0.31846	-0.82872	-0.00000
C	1.63902	-1.28926	-0.00002
C	2.29937	-0.03442	0.00003
N	1.55781	1.12151	-0.00002
H	-2.96959	1.23910	0.00000
H	-2.93823	-1.27748	0.00000
H	3.37999	0.12906	0.00002
H	-0.72956	2.45945	-0.00000

Compound: [1-5an]⁻

SCF energy: -395.21503471 a.u.

N	0.23075	-0.64442	-0.00000
C	-0.94787	-1.33736	0.00000

C	-2.10006	-0.60406	0.00000
C	-2.16542	0.86901	0.00000
N	-0.93459	1.47561	0.00000
C	0.21025	0.75258	0.00000
C	1.57569	1.11393	-0.00000
C	2.27615	-0.09555	0.00000
N	1.48611	-1.18613	-0.00000
H	-0.84436	-2.42241	-0.00000
H	1.96346	2.12268	-0.00000
H	3.35224	-0.24383	0.00000
H	-3.03955	-1.16315	0.00000

Compound: [1-6an]$^{-}$

SCF energy: -395.23263907 a.u.

N	0.23758	0.65115	0.00000
C	-0.94673	1.37706	0.00000
C	-2.19332	0.78625	0.00000
C	-2.06258	-0.64741	-0.00000
N	-0.96478	-1.41519	0.00000
C	0.20492	-0.73882	0.00000
C	1.55468	-1.14638	0.00000
C	2.27846	0.04806	0.00000
N	1.50470	1.15228	-0.00000
H	-2.98039	-1.25294	0.00000
H	-0.75008	2.45347	0.00000
H	1.91830	-2.16418	-0.00000

H	3.35710	0.17349	0.00000

Compound: [1-7an]$^-$

SCF energy: -395.24720100 a.u.

N	0.23773	-0.69105	0.00000
C	-0.86877	-1.53133	0.00000
C	-2.03301	-0.73571	0.00000
C	-2.02803	0.67104	0.00000
N	-0.93213	1.42530	0.00000
C	0.22348	0.71905	0.00000
C	1.56107	1.12932	0.00000
C	2.28870	-0.07036	0.00000
N	1.52668	-1.16865	0.00000
H	-3.00509	-1.23610	0.00000
H	-2.97529	1.22453	0.00000
H	1.92454	2.14831	0.00000
H	3.36924	-0.18800	0.00000

Electrophile Affinities (Eα)

The recently introduced concept of electrophile affinity (Eα) is defined according to equation S3.[S8] Good to excellent correlations are observed between Eα values and relative rates and regioselectivities in chlorination, bromination, nitration and benzylation of simple aromatics and, in principle, this correlation should hold for any S_EAr process which proceeds via the rate-limiting formation of a Wheland-like intermediate.[S8]

$$E\alpha \text{ (kcal/mol)} = [E_{\text{arene}} + E_{\text{electropile}}] - E_{\text{arenium ion}} \qquad \text{(S3)}$$

In this case the Eα were calculated in the gas-phase, with arene = **1**; electrophile = Br^+ ion and arenium = the sigma-complexes [**1**-Br]$^+$ with the site of Br^+ substitution varied between the C2, C3, C5, C6 and C7 positions, using the B3LYP functional,[S3] combined with 6-311+G(2d,2p) basis set.[S9] Cartesian coordinates and energies of all optimized structures are given below. All calculated

structures showed no imaginary frequencies, showing that they are intermediates. Figure S4 shows a representative example of an arenium complex, namely that of the C3-brominated sigma-complex [**1**-3Br]$^+$, and summarizes the values obtained for Eα at all the carbon-positions.

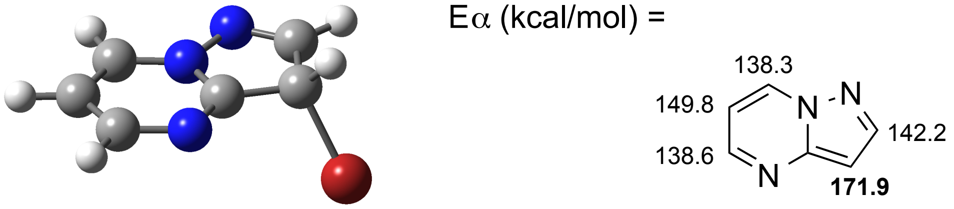

Figure S4. Left: representative example of arenium ion, brominated at the most favoured (C3) position, [**1**-3Br]$^+$. Right: summary of relative Eα values for the gas-phase bromination with Br$^+$ (B3LYP-6-311+G(2d,2p)).

Optimized Cartesian Coordinates of Computed (B3LYP/6-311+G(2d,2p)) Structures

Compound: Br$^+$

SCF energy: -2573.593988 a.u.

Compound: 1

SCF energy: -395.98551480 a.u.

C	0.88103	-1.36797	-0.00003
C	-0.25546	0.75162	-0.00016
C	2.01487	0.74181	-0.00004
H	0.77961	-2.44213	-0.00006
H	2.94079	1.30468	-0.00008
C	-1.59206	1.12964	-0.00053
H	-1.97228	2.13395	-0.00085
N	-0.26979	-0.65441	-0.00011
N	-1.51834	-1.16018	-0.00030
C	-2.30335	-0.07403	0.00040

H	-3.37203	-0.21113	0.00066
C	2.05715	-0.67777	0.00000
H	2.99839	-1.20400	0.00001
N	0.90315	1.43959	-0.00016

Compound: [1-2Br]$^+$

SCF energy: -2969.80614966 a.u.

C	0.91170	-1.41328	-0.02233
C	-0.27920	0.73469	0.03172
C	1.98259	0.77516	-0.00379
H	0.84069	-2.48975	-0.03712
H	2.90594	1.34093	-0.00932
C	-1.58199	1.09879	0.07261
H	-1.97630	2.09974	0.10944
N	-0.28929	-0.71692	0.00897
N	-1.44221	-1.25769	0.03221
C	-2.37107	-0.14688	0.07967
H	-3.11146	-0.25343	-0.71339
C	2.04034	-0.67327	-0.02918
H	3.00034	-1.16808	-0.05248
N	0.87303	1.44276	0.02605
Br	-3.38605	-0.31355	1.77126

Compound: [1-3Br]$^+$

SCF energy: -2969.85349188 a.u.

C	0.85913	-1.37102	0.17026
C	-0.32672	0.64082	-0.20716

C	1.92240	0.72668	-0.23387
H	0.79208	-2.42871	0.37854
H	2.80923	1.33584	-0.34850
C	-1.77219	1.01103	-0.28023
H	-2.05545	1.81985	0.38848
N	-0.29142	-0.69340	0.05661
N	-1.57555	-1.27380	0.20172
C	-2.40458	-0.31200	0.02369
H	-3.46731	-0.49672	0.07929
C	2.02294	-0.64390	0.01578
H	2.98286	-1.13027	0.09409
N	0.74756	1.36655	-0.34592
Br	-2.27237	1.58686	-2.09715

Compound: [1-5Br]$^{+}$

SCF energy: -2969.80031797 a.u.

C	0.87306	-1.51406	-0.07141
C	-0.15339	0.74261	-0.02192
C	2.15547	0.59036	-0.11061
H	0.70920	-2.58047	-0.07347
C	-1.52658	1.17817	0.04766
H	-1.85120	2.20382	0.05883
N	-0.25475	-0.71532	-0.00446
N	-1.46609	-1.10655	0.06302
C	-2.26604	0.04455	0.09794
H	-3.33263	-0.08545	0.15729
C	2.05912	-0.88464	-0.12752

H	2.97474	-1.45393	-0.18181
N	0.95256	1.36047	-0.08713
H	2.80291	0.98423	-0.89368
Br	3.12663	1.12675	1.56269

Compound: [1-6Br]$^+$

SCF energy: -2969.81822849 a.u.

C	0.80014	-1.41903	0.01002
C	-0.23806	0.75091	0.01253
C	2.03430	0.75919	0.05745
H	0.69691	-2.49661	-0.02834
H	2.97513	1.29751	0.06311
C	-1.53375	1.12559	0.00420
H	-1.91781	2.13040	0.01046
N	-0.26394	-0.69258	-0.00415
N	-1.56783	-1.18843	-0.02773
C	-2.29868	-0.10995	-0.01947
H	-3.37401	-0.20287	-0.03327
C	2.10192	-0.73592	0.11497
H	2.85024	-1.16364	-0.54763
N	0.94456	1.43071	0.01683
Br	2.69754	-1.29706	1.94871

Compound: [1-7Br]$^+$

SCF energy: -2969.79991718 a.u.

C	0.79037	-1.54658	-0.05374
C	-0.27354	0.68002	0.10315

C	2.01795	0.60573	0.07964
H	0.72750	-2.37064	-0.75540
H	2.93230	1.17889	0.12083
C	-1.62287	1.13114	0.09423
H	-1.94618	2.15209	0.19930
N	-0.35659	-0.71001	-0.09630
N	-1.58961	-1.11646	-0.22231
C	-2.37579	-0.00496	-0.11021
H	-3.44298	-0.11402	-0.19558
C	2.03624	-0.75595	-0.06653
H	2.96492	-1.29976	-0.15795
N	0.86149	1.31352	0.17957
Br	0.95329	-2.42903	1.76016

8. ^{1}H and ^{13}C NMR Spectra of Isolated Products

7-phenylpyrazolo[1,5-a]pyrimidine, 2a

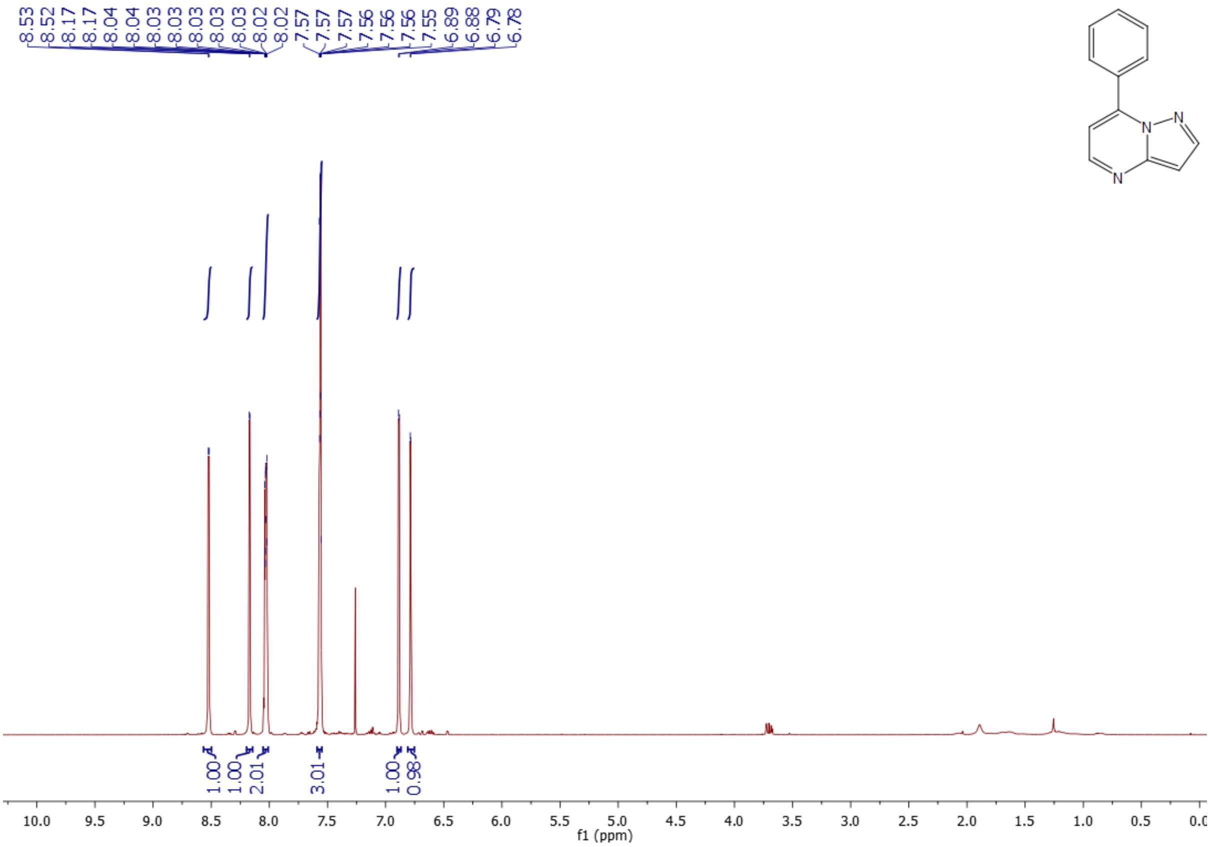

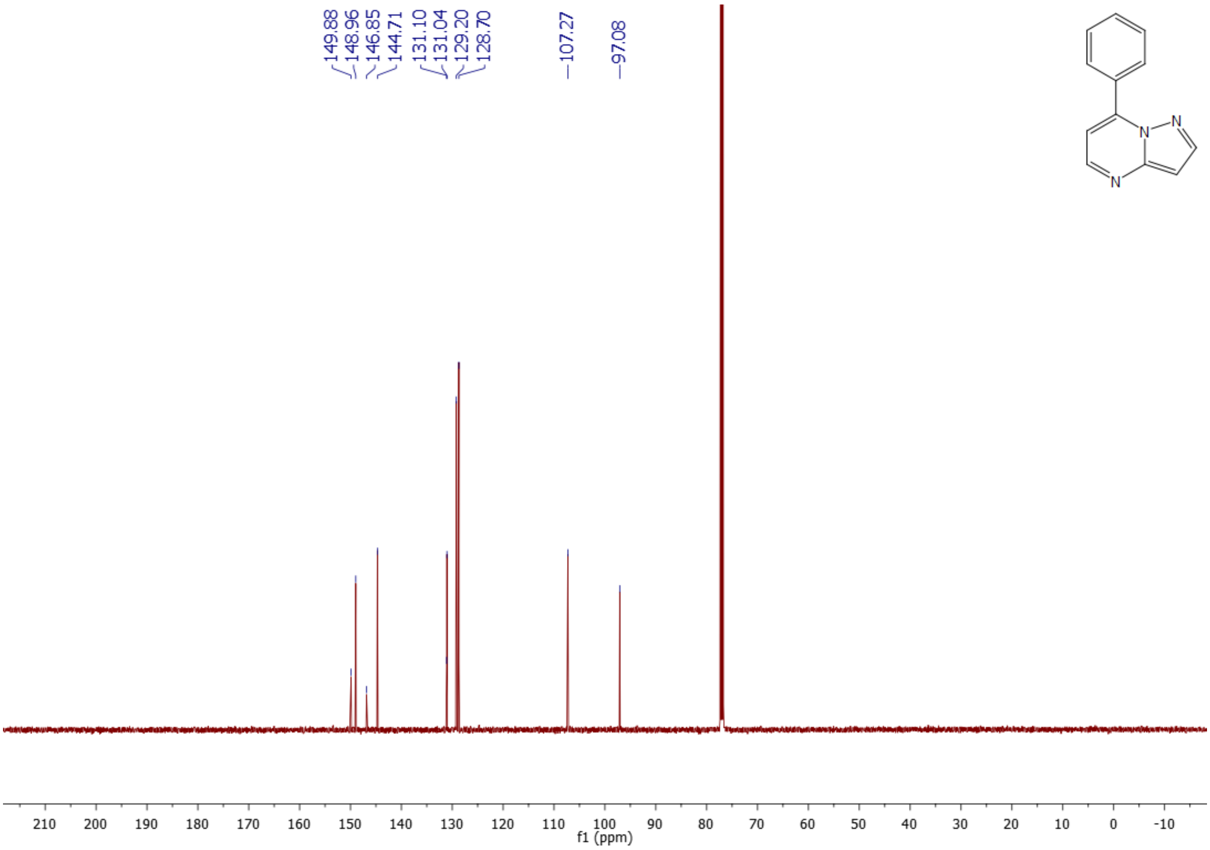

7-(p-tolyl)pyrazolo[1,5-a]pyrimidine, 2b

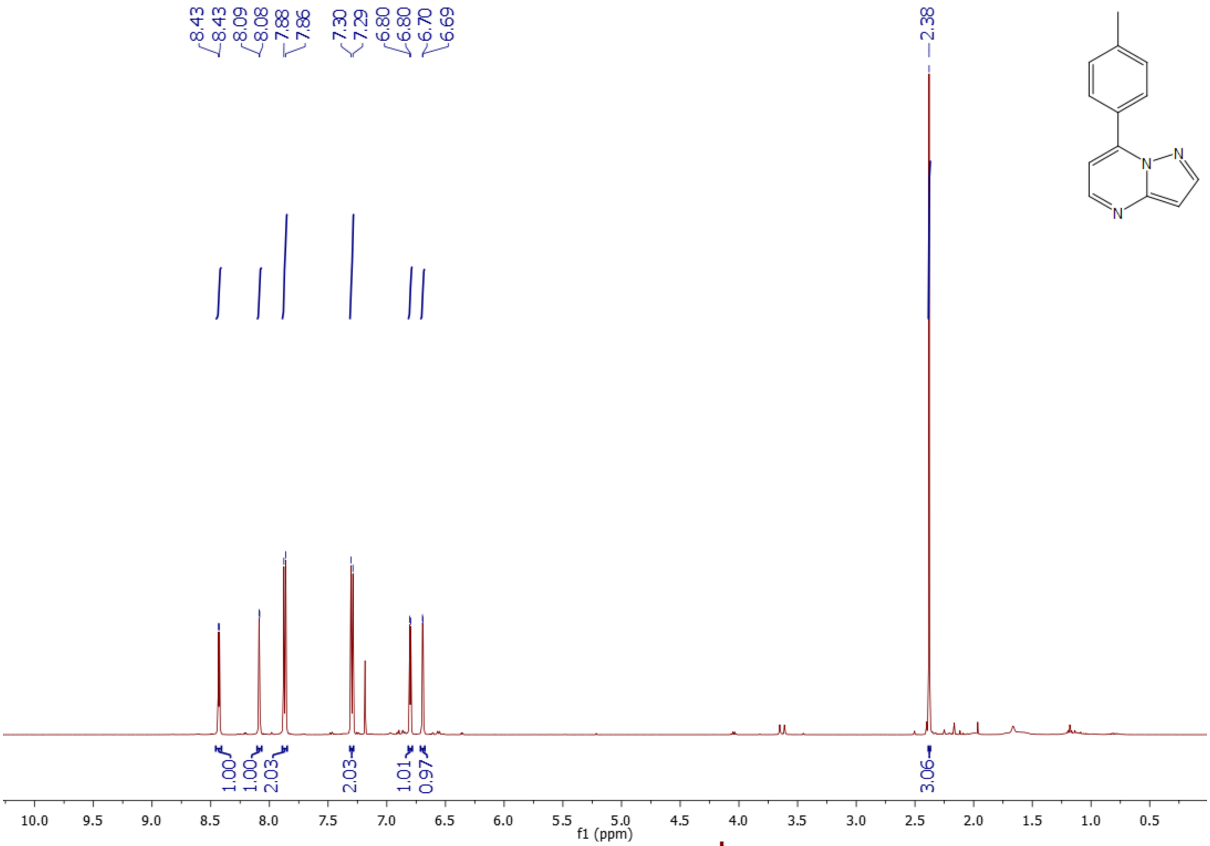

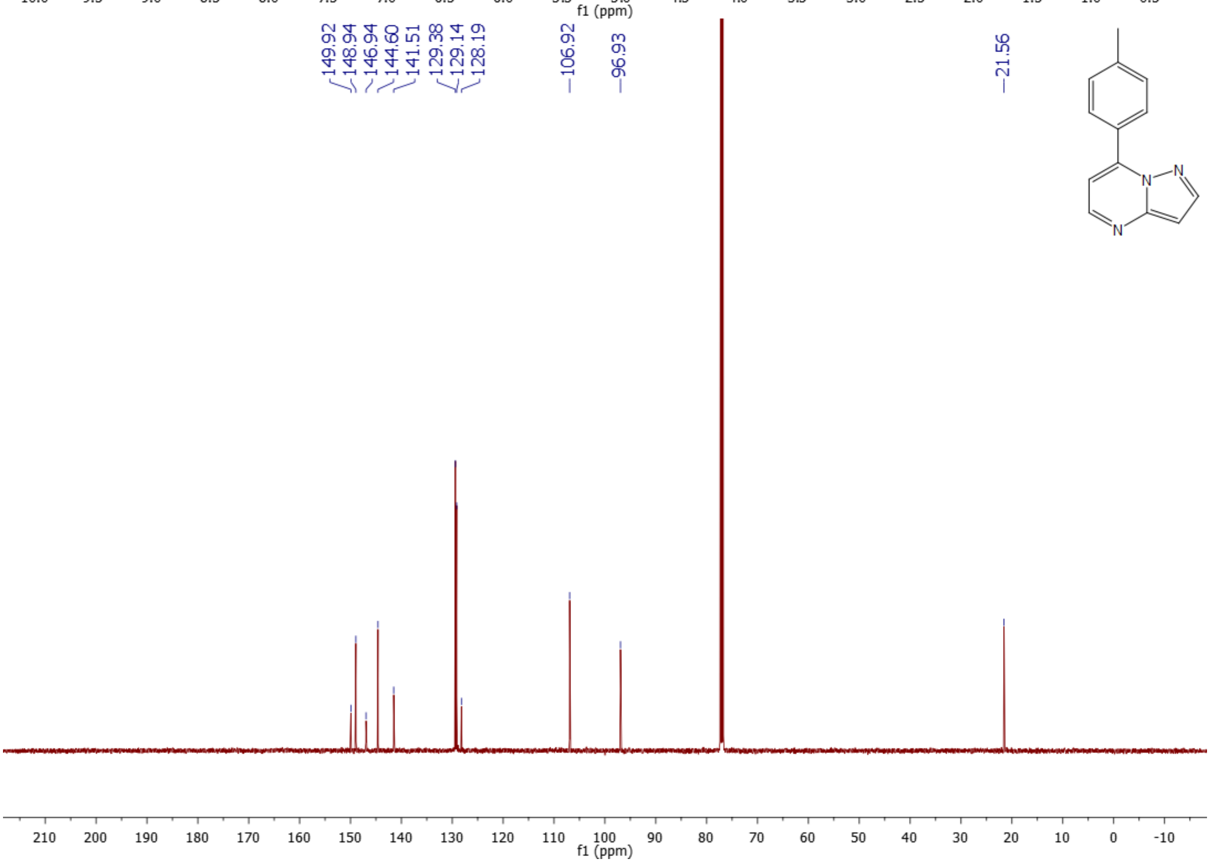

7-(4-methoxyphenyl)pyrazolo[1,5-a]pyrimidine, 2c

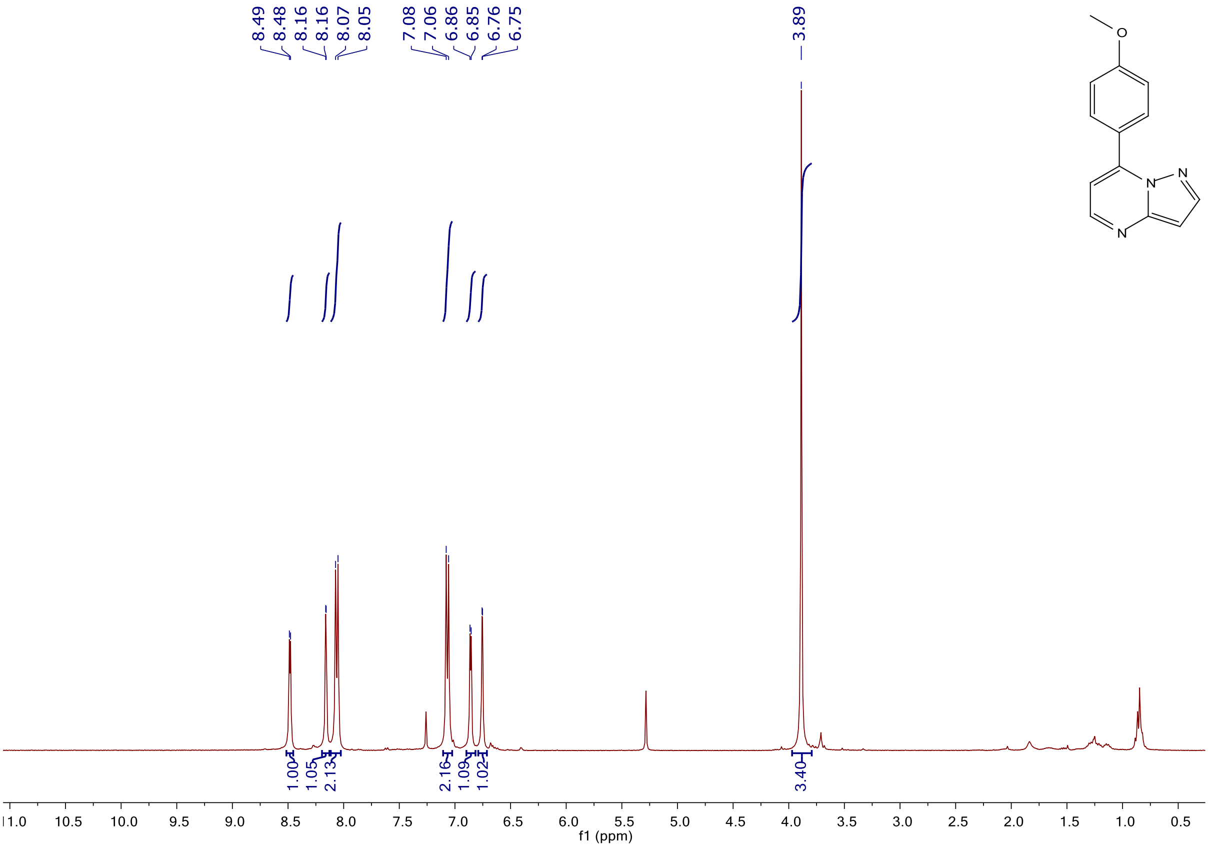

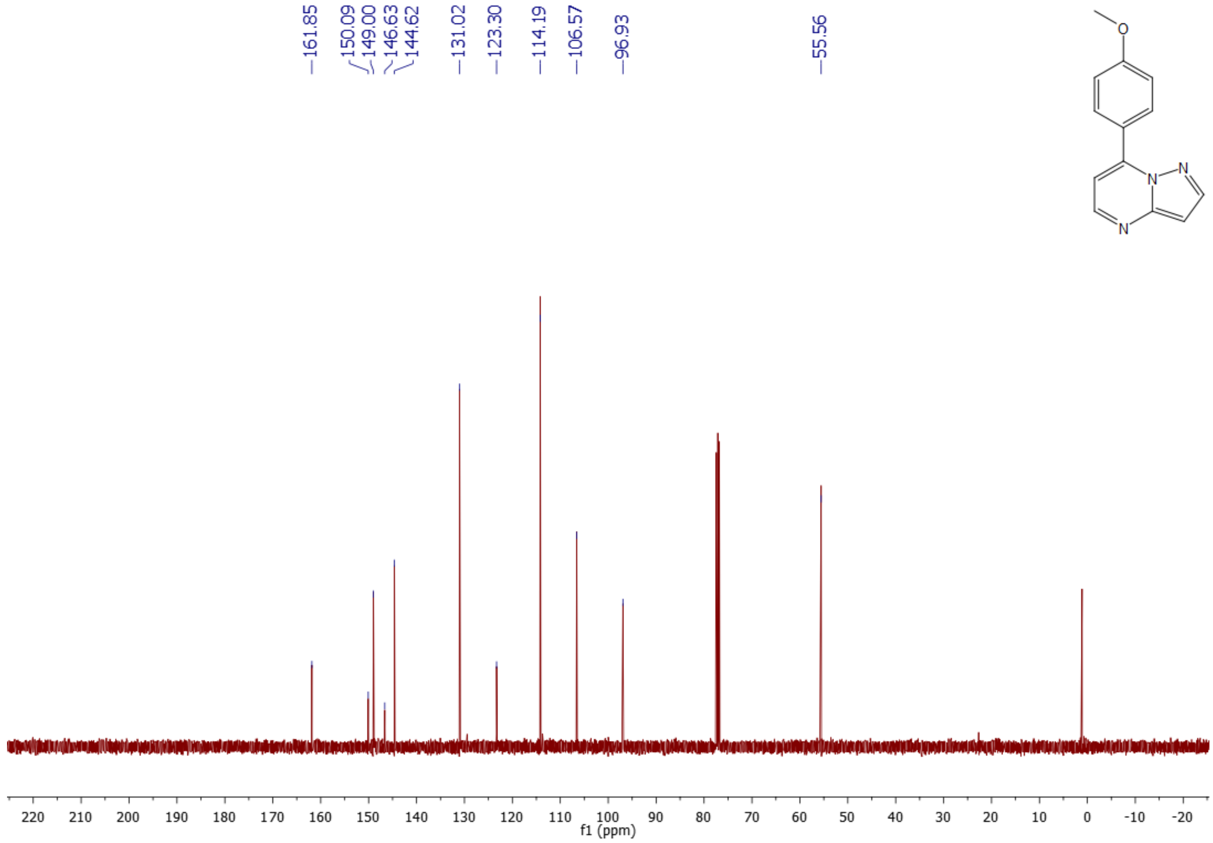

7-(4-fluorophenyl)pyrazolo[1,5-a]pyrimidine, 2d

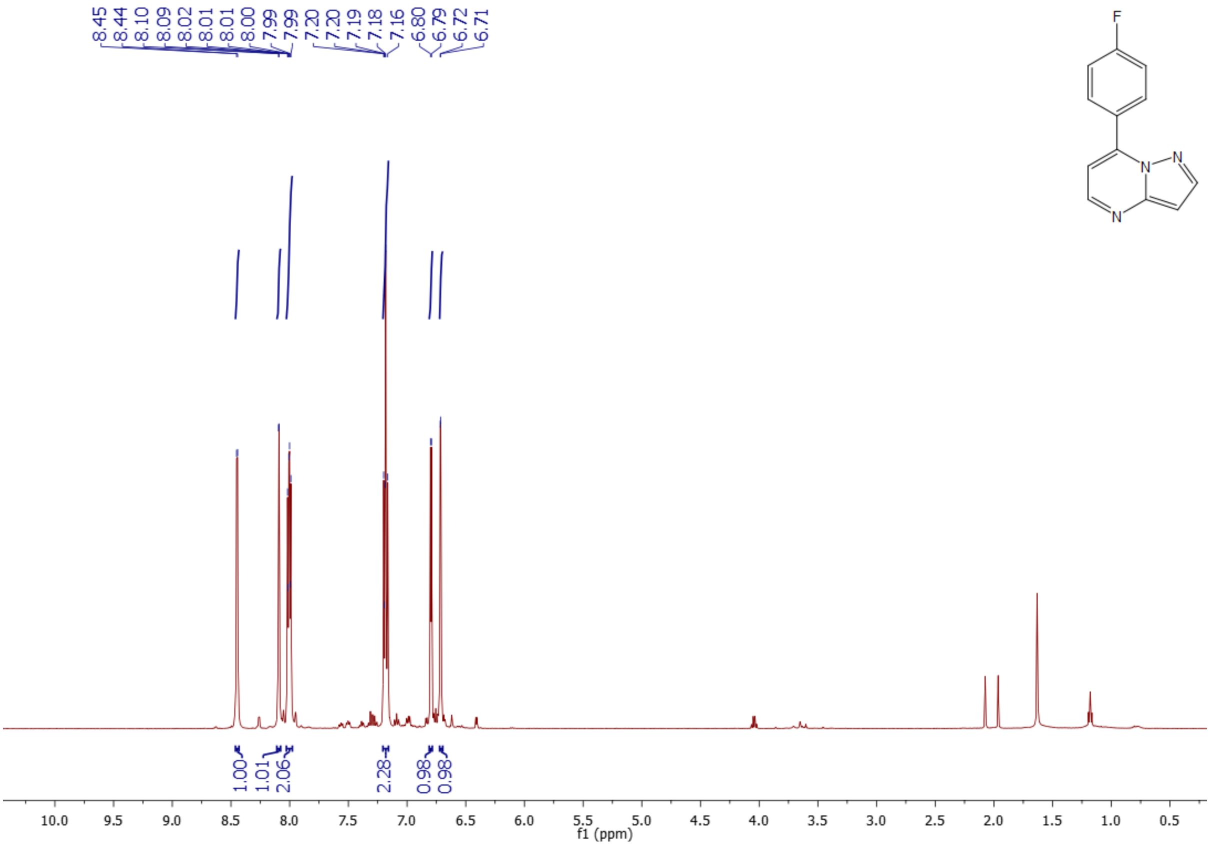

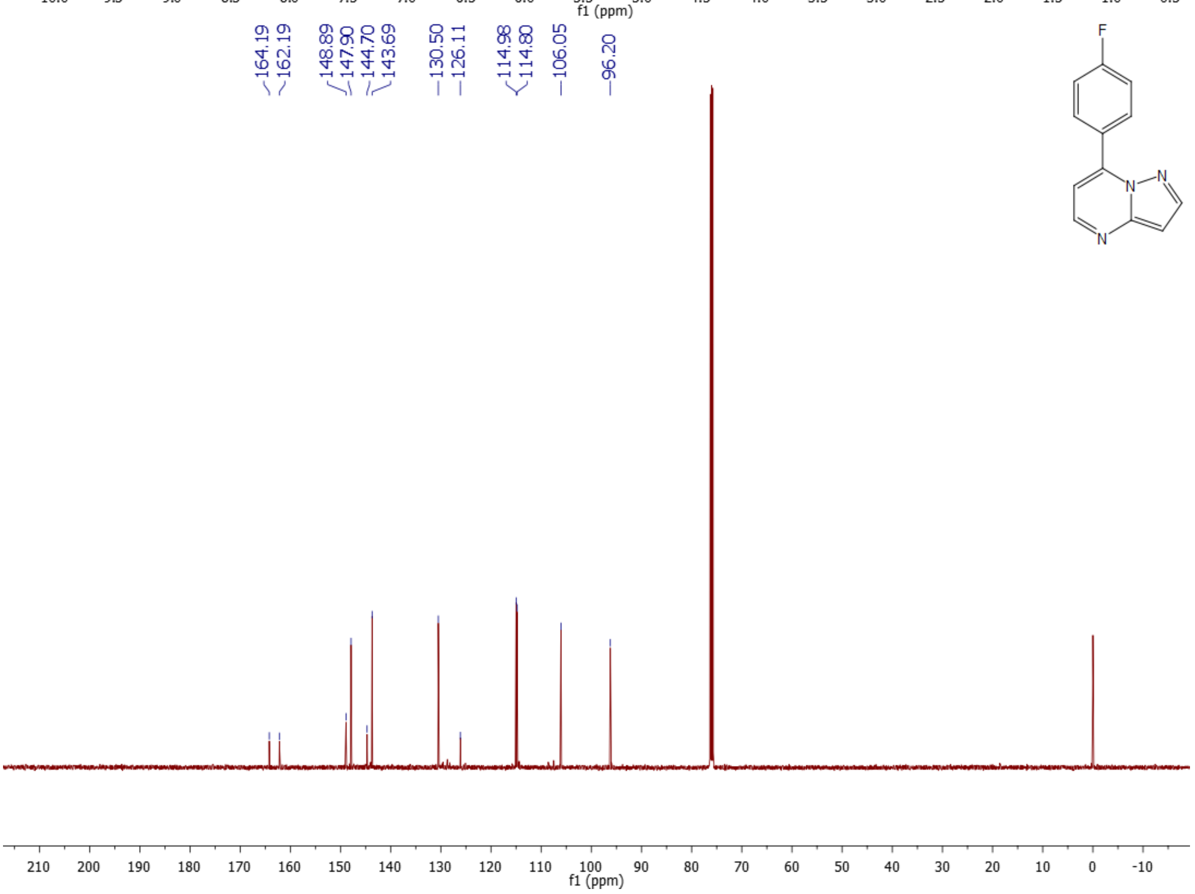

7-(4-(trifluoromethyl)phenyl)pyrazolo[1,5-a]pyrimidine, 2e

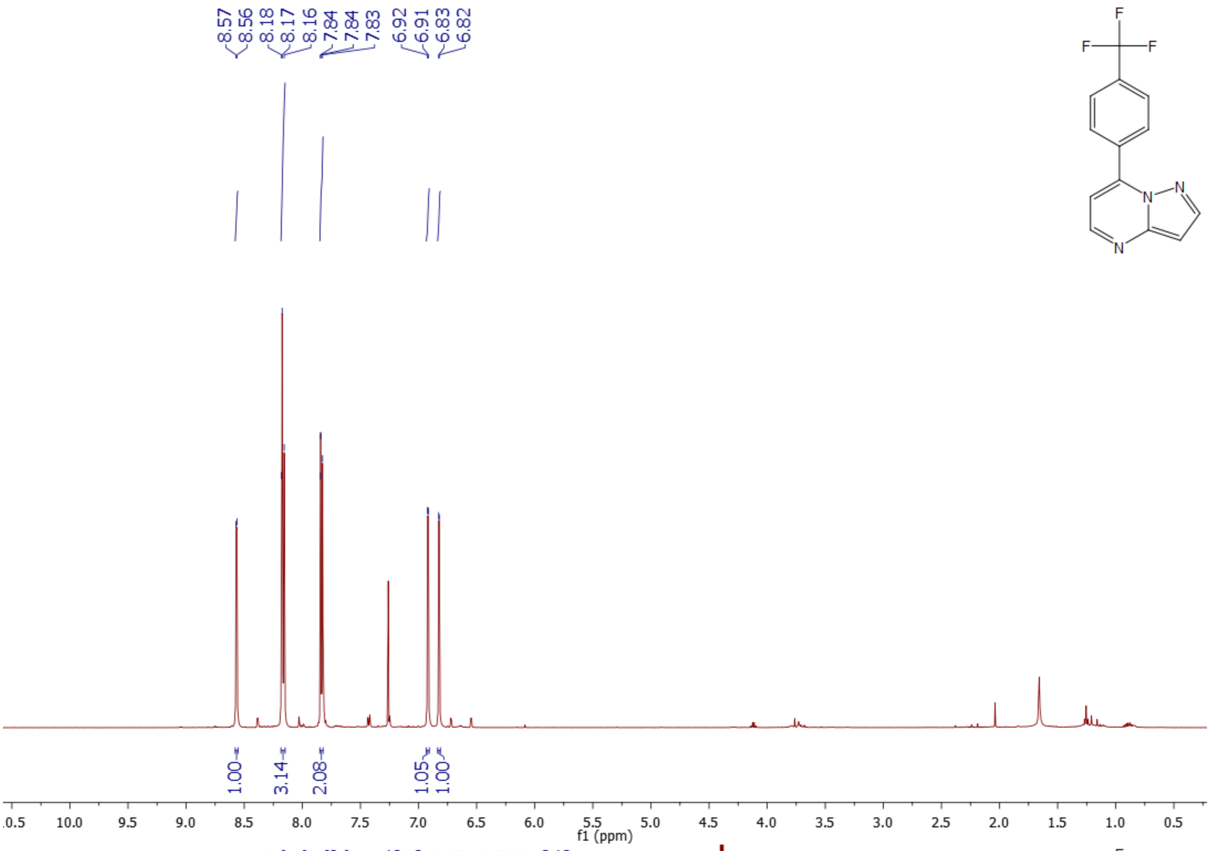

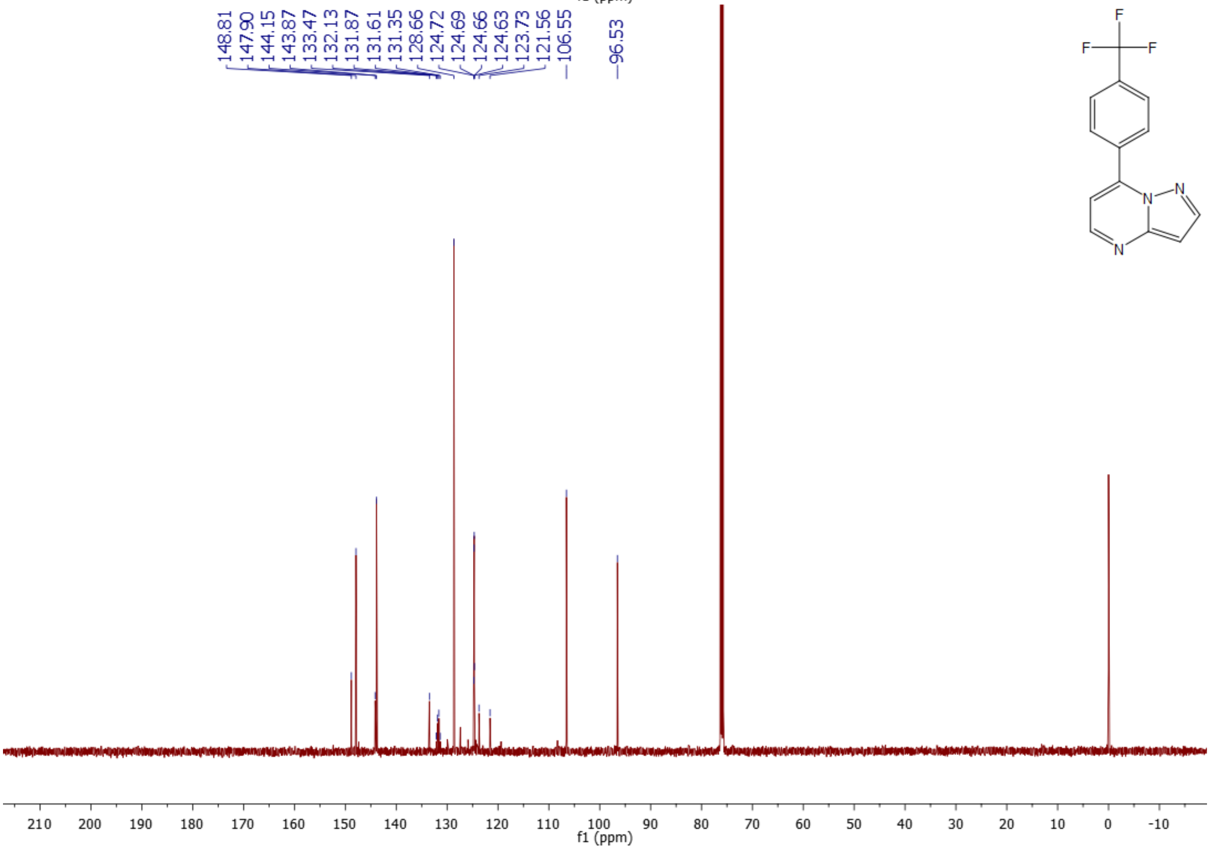

Methyl 4-(pyrazolo[1,5-a]pyrimidin-7-yl)benzoate, 2f

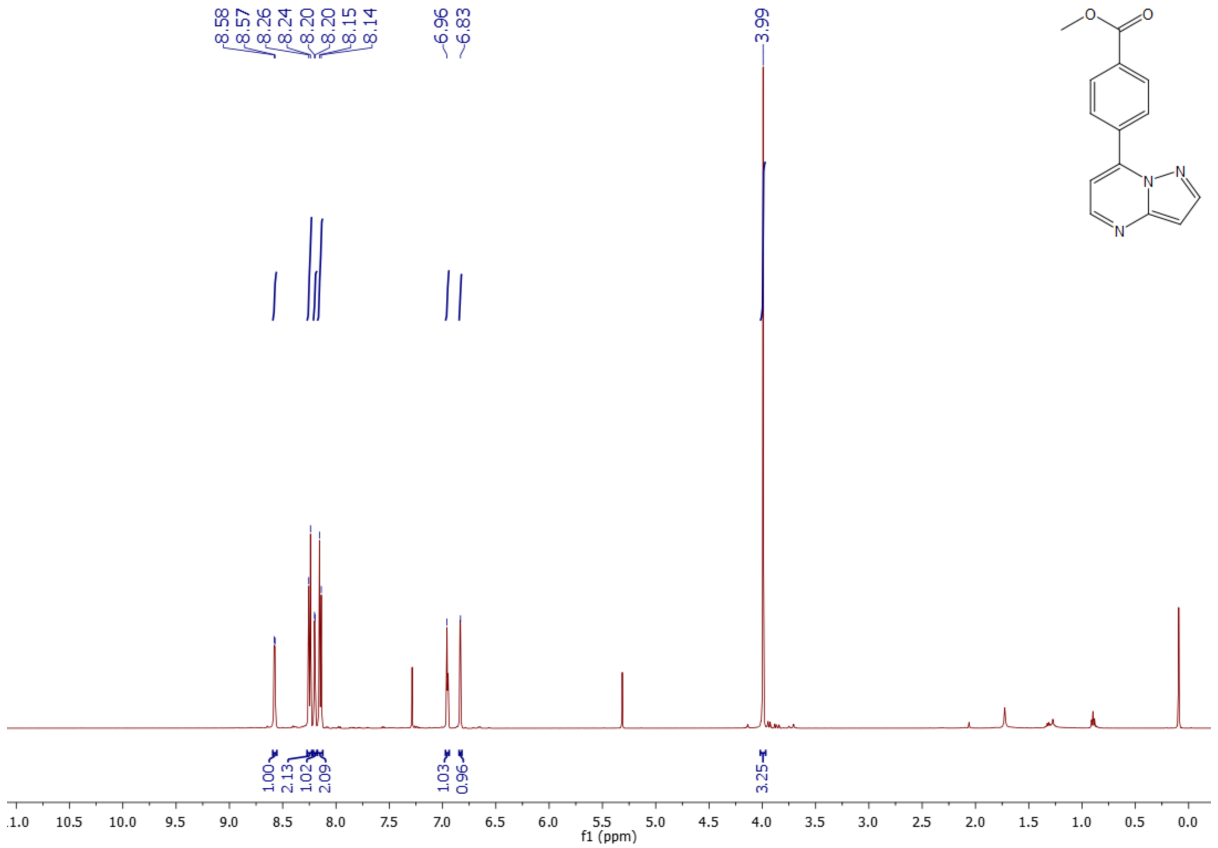

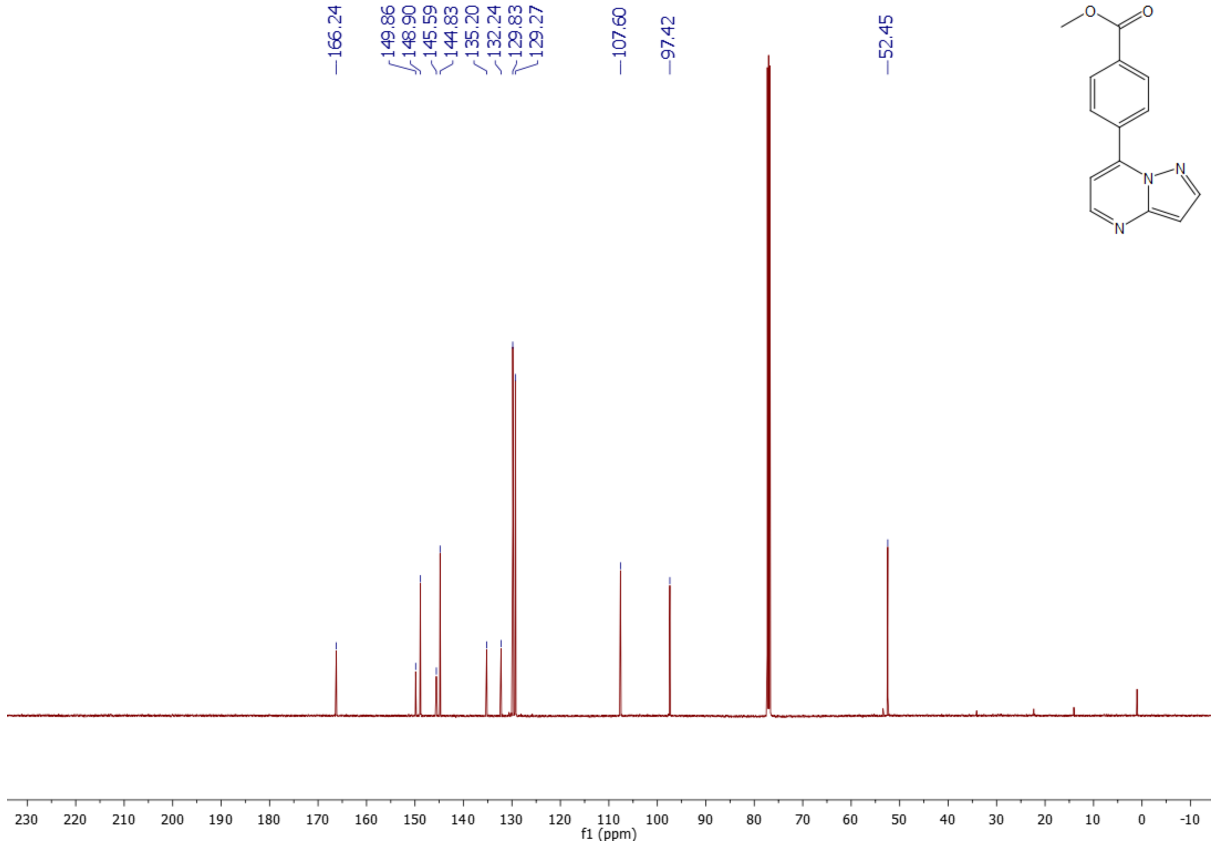

7-(4-chlorophenyl)pyrazolo[1,5-a]pyrimidine, 2g

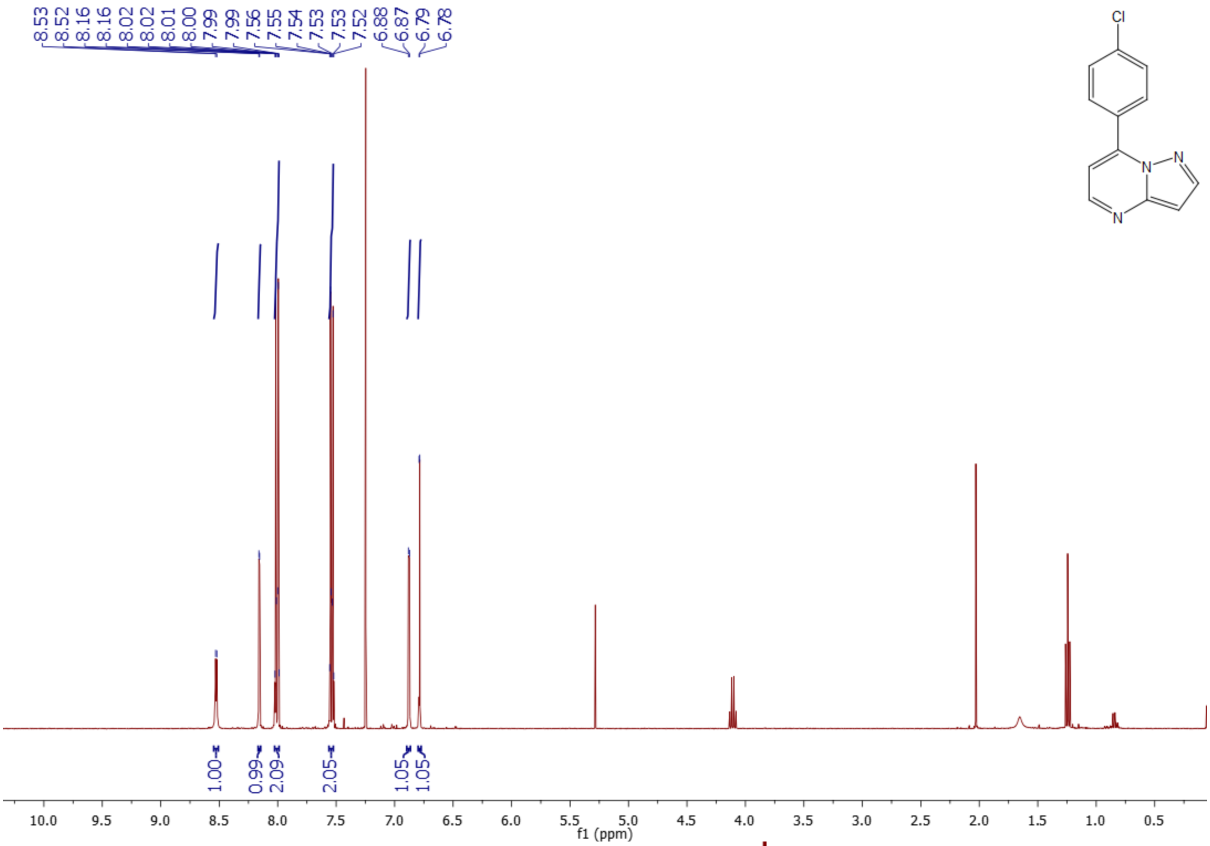

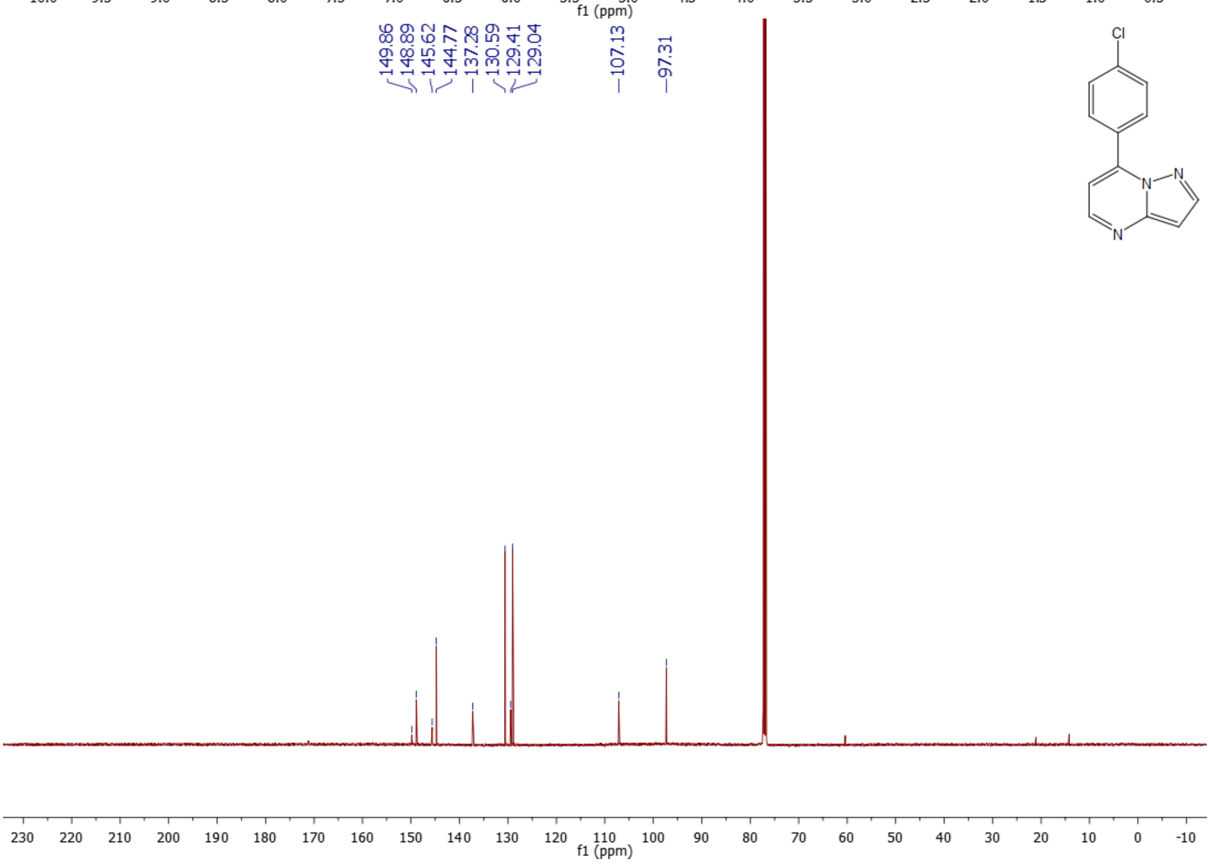

7-(o-tolyl)pyrazolo[1,5-a]pyrimidine, 2h

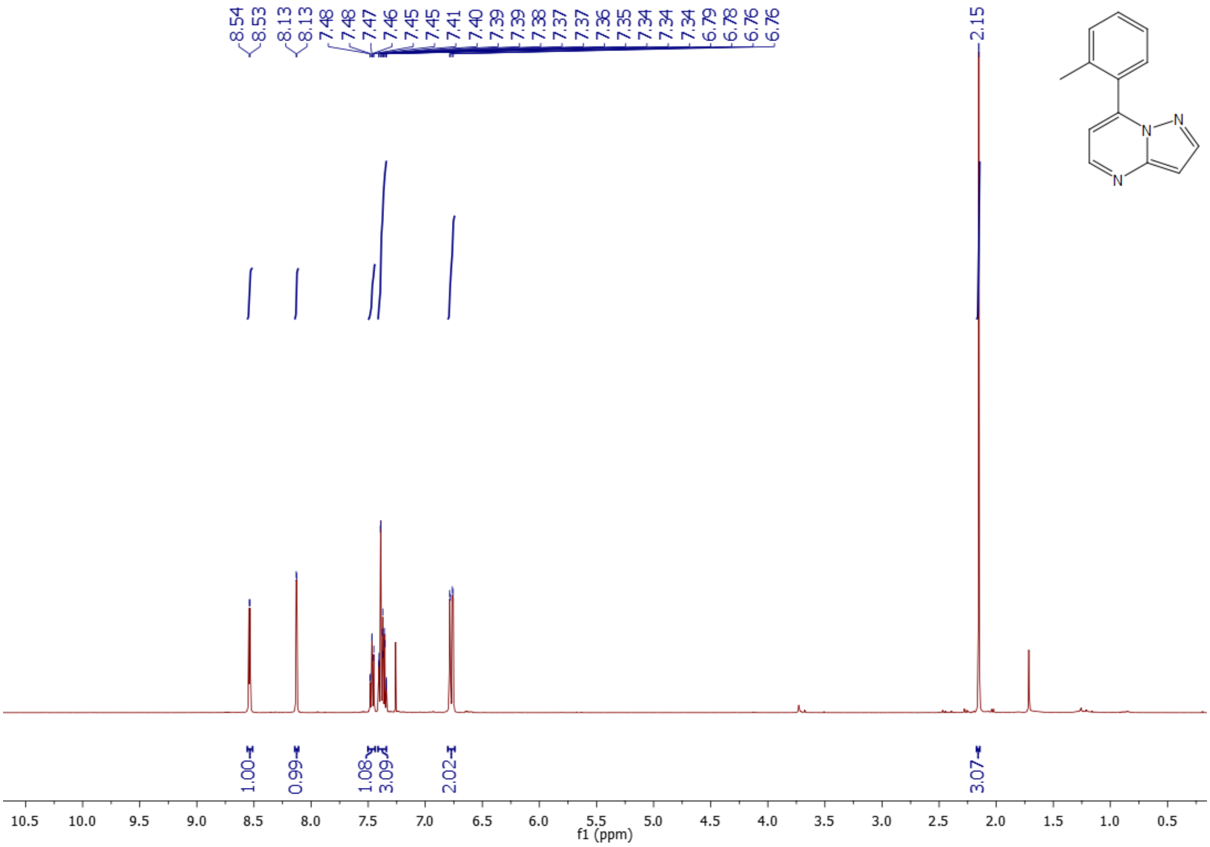

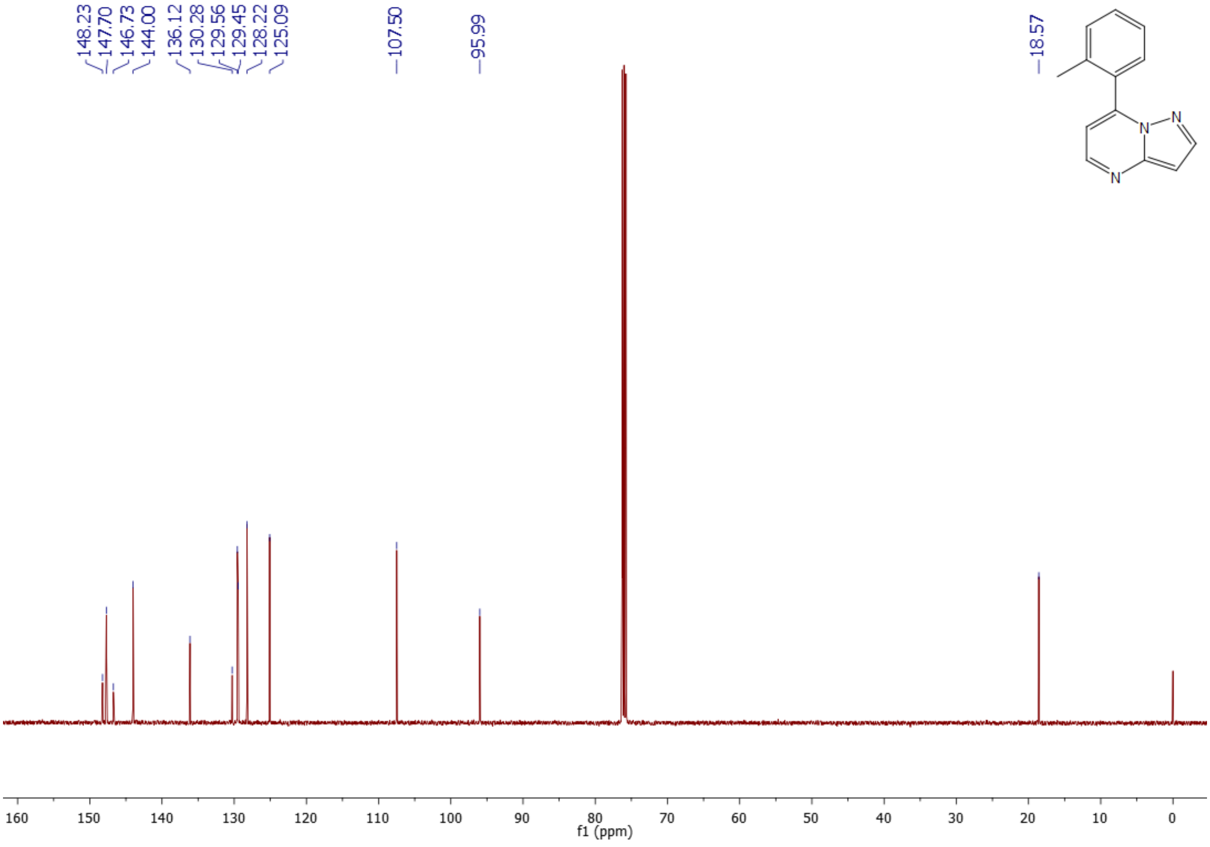

7-(2-nitrophenyl)pyrazolo[1,5-a]pyrimidine, 2i

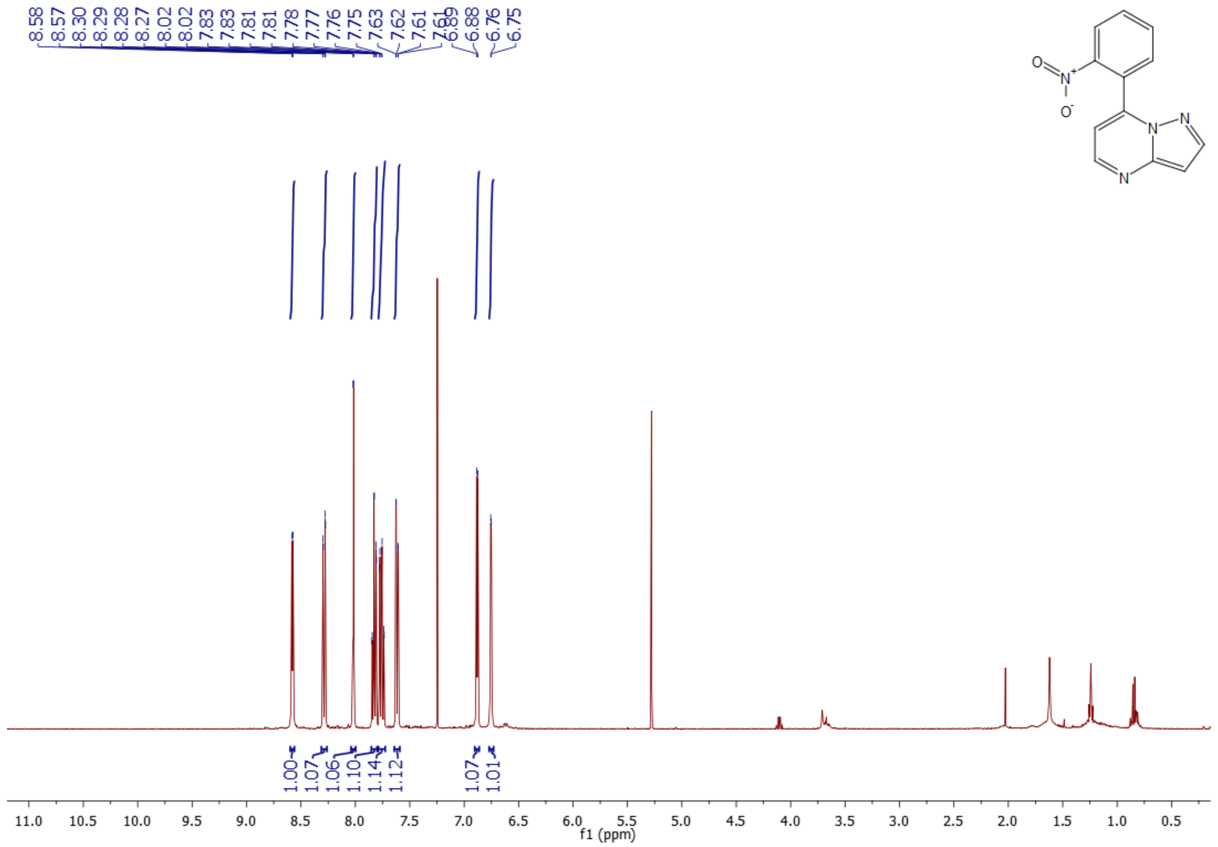

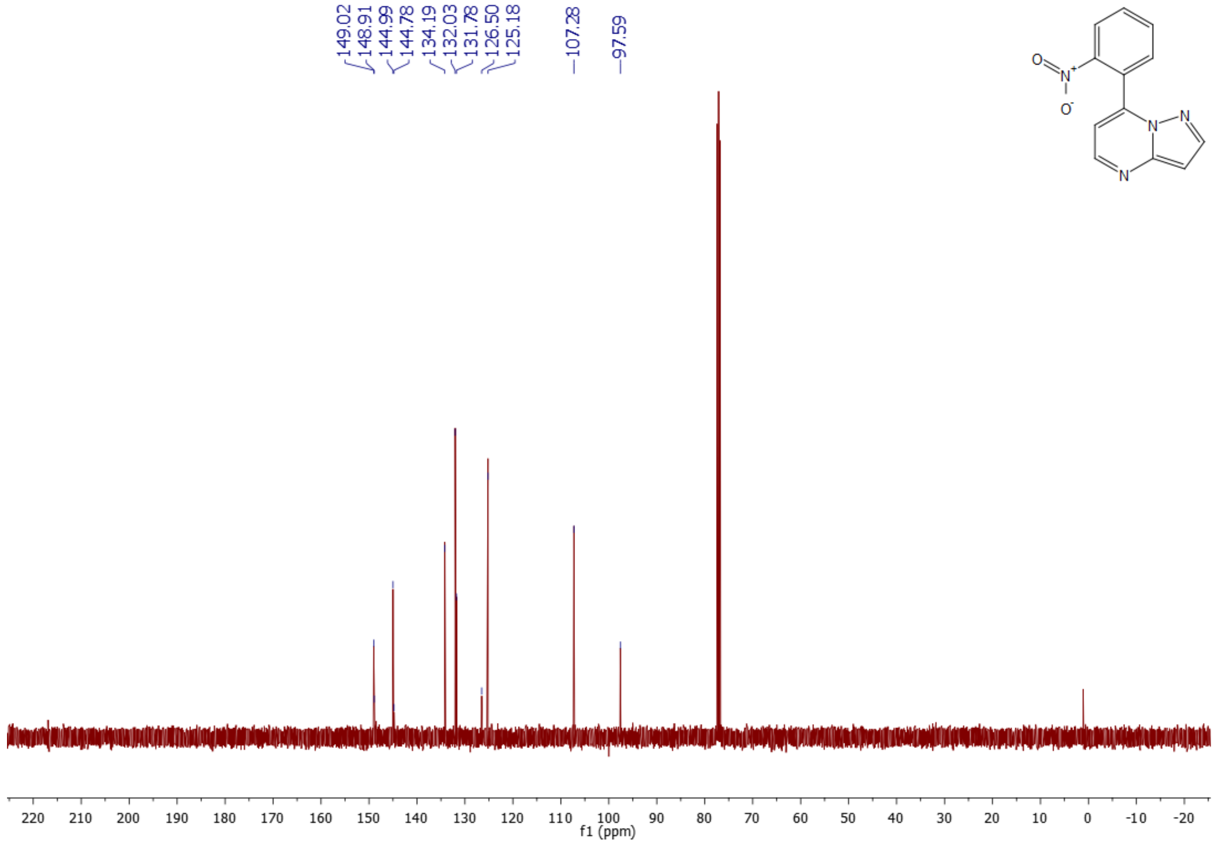

7-(m-tolyl)pyrazolo[1,5-a]pyrimidine, 2j

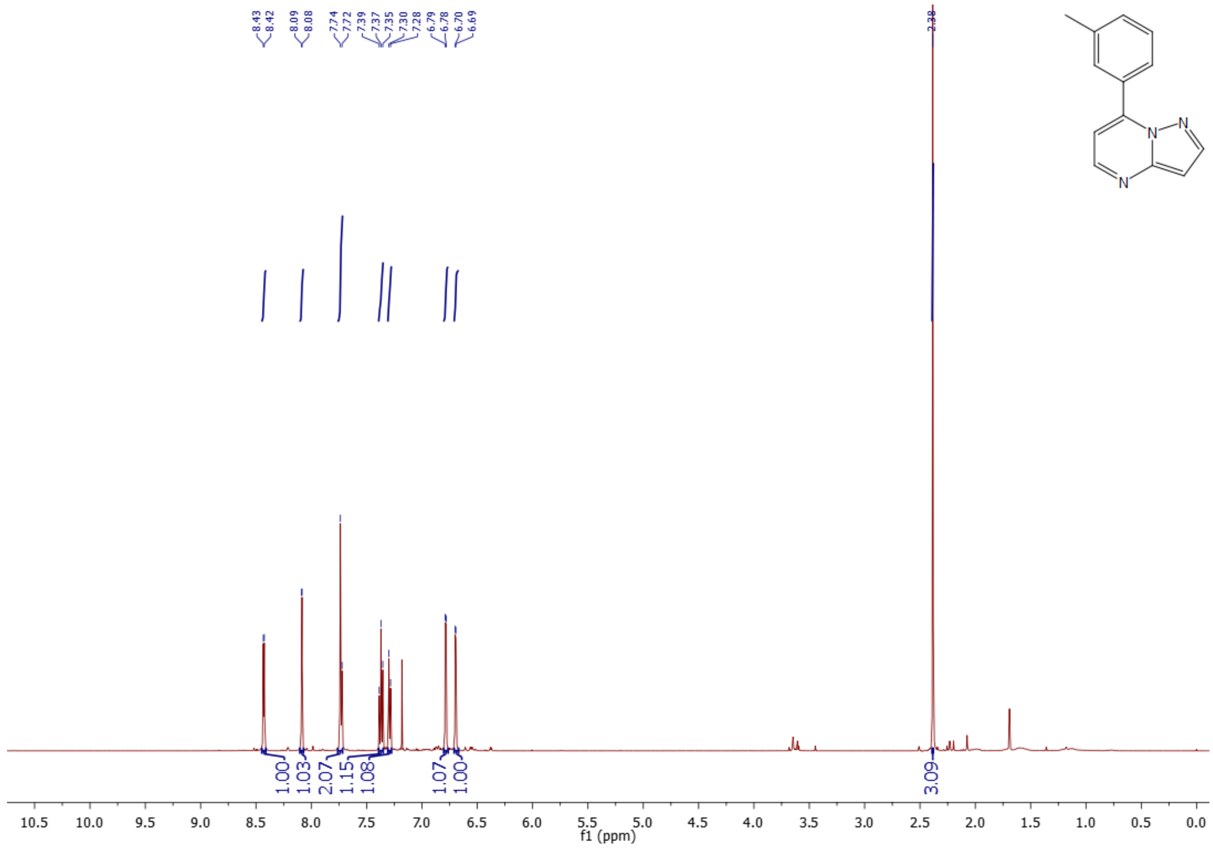

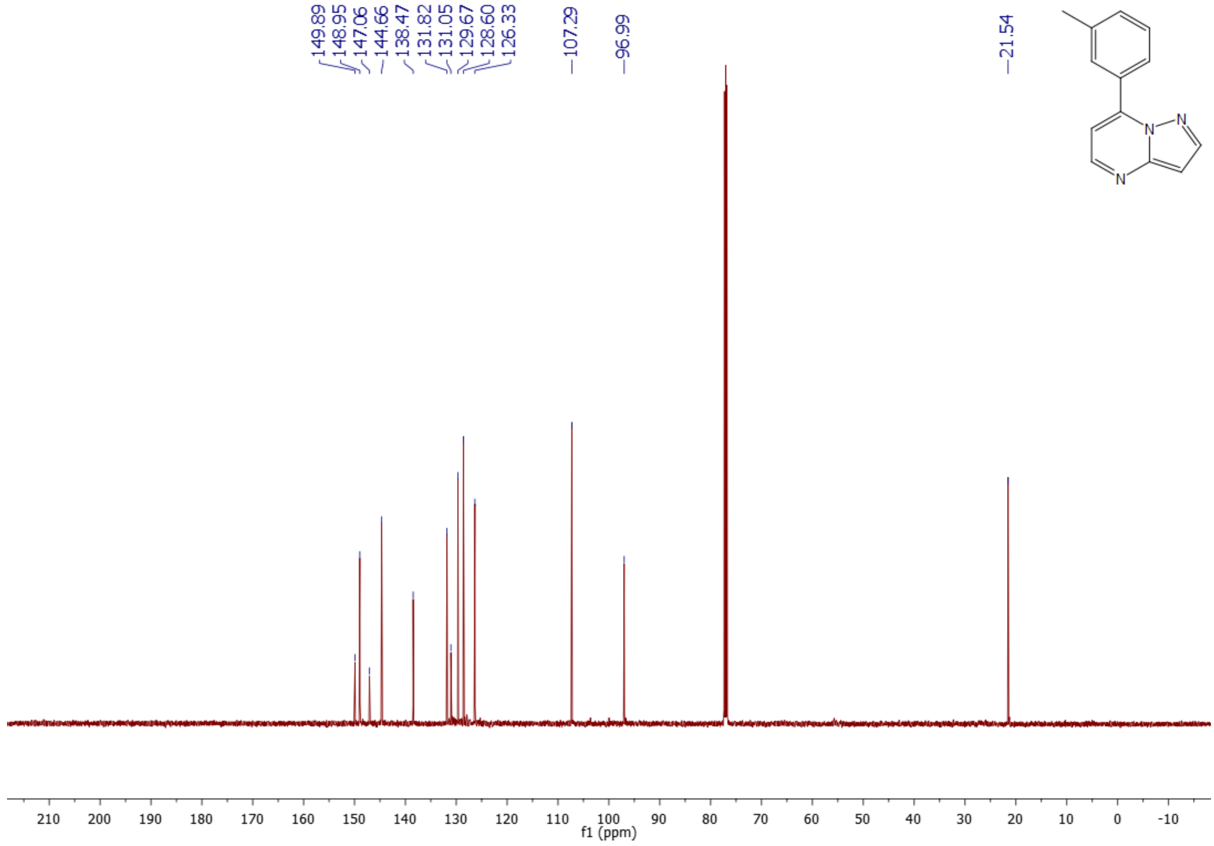

7-(2,6-dimethylphenyl)pyrazolo[1,5-a]pyrimidine, 2k

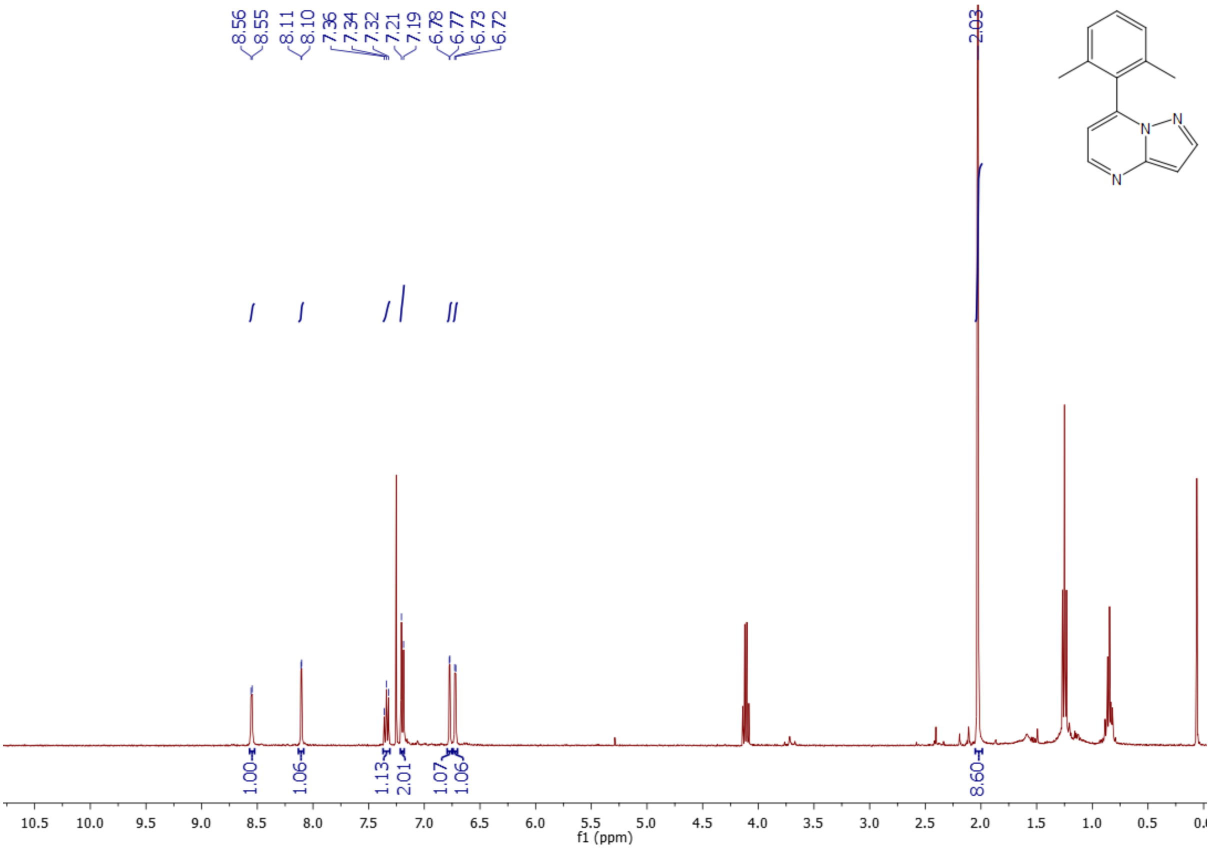

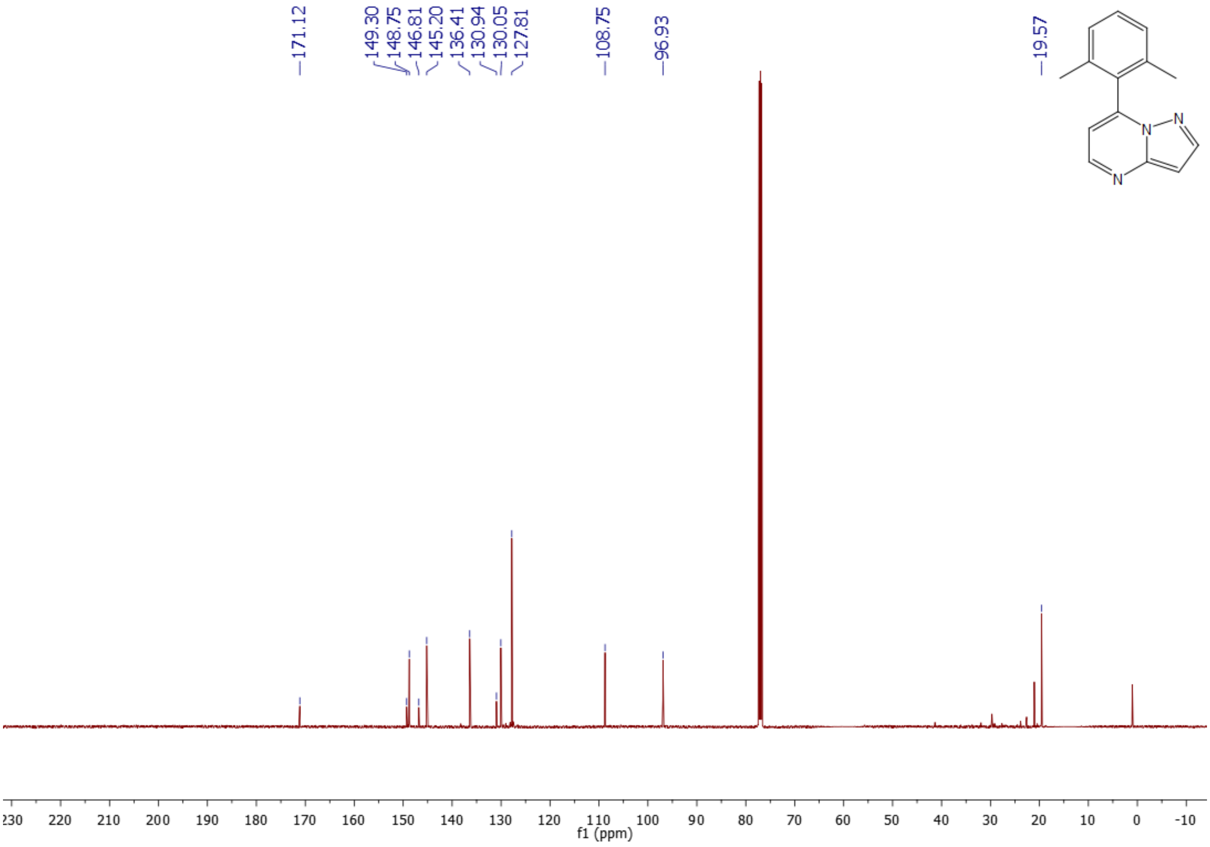

7-(naphthalen-2-yl)pyrazolo[1,5-a]pyrimidine, 2l

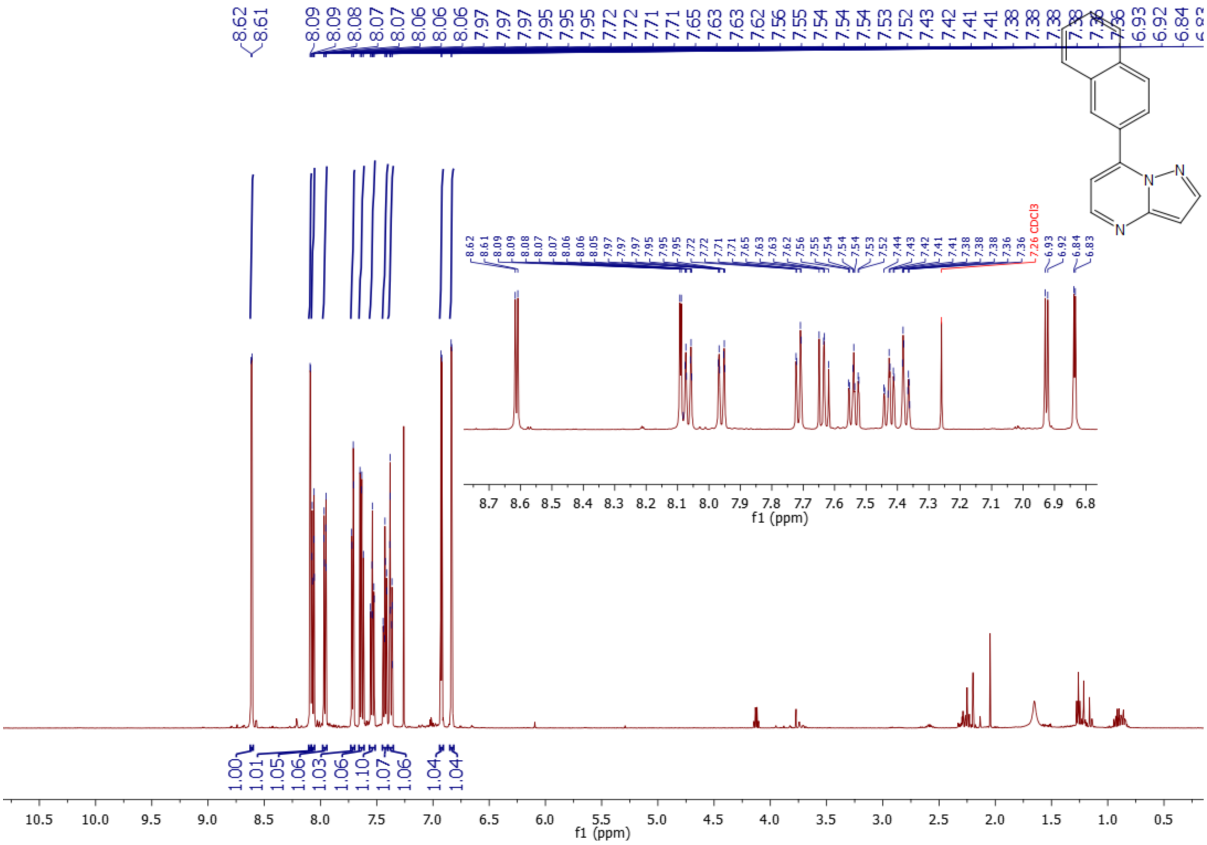

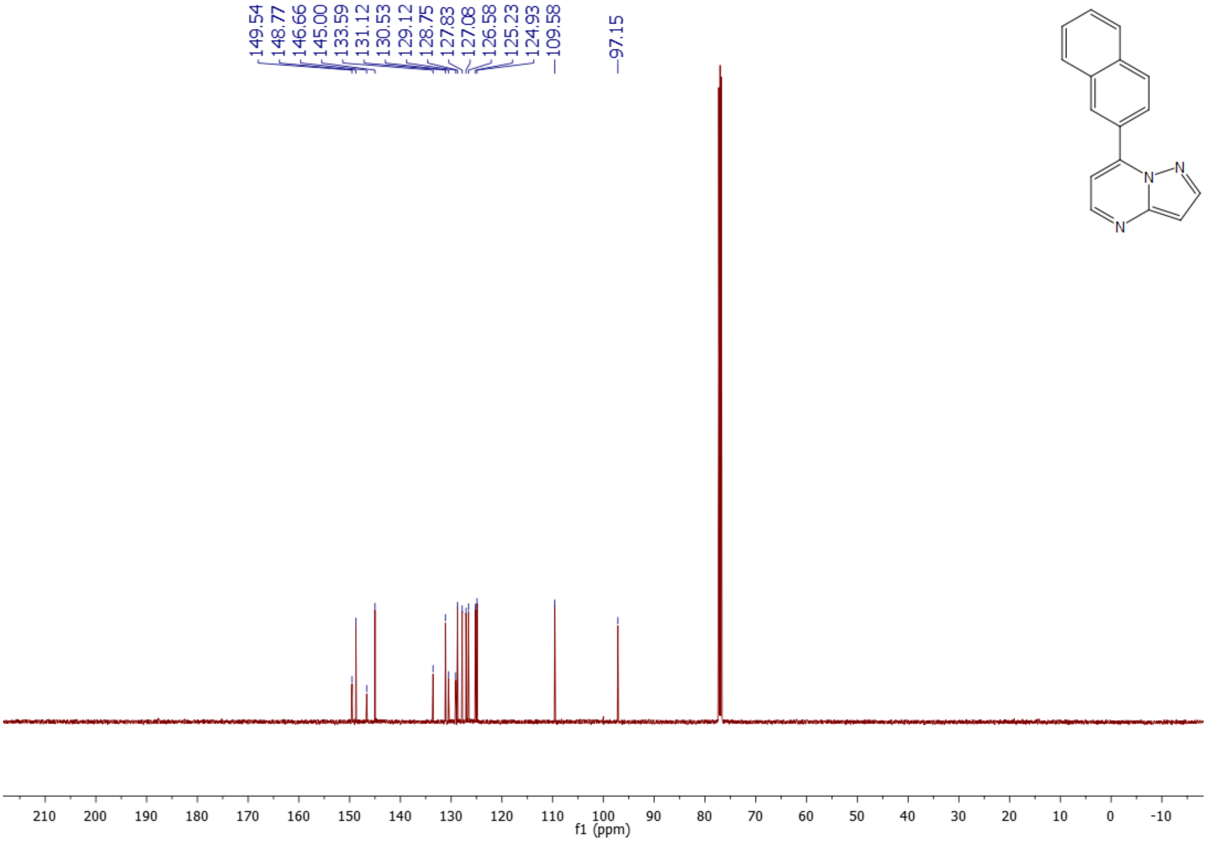

7-(2-methylpyridin-4-yl)pyrazolo[1,5-a]pyrimidine, 2m

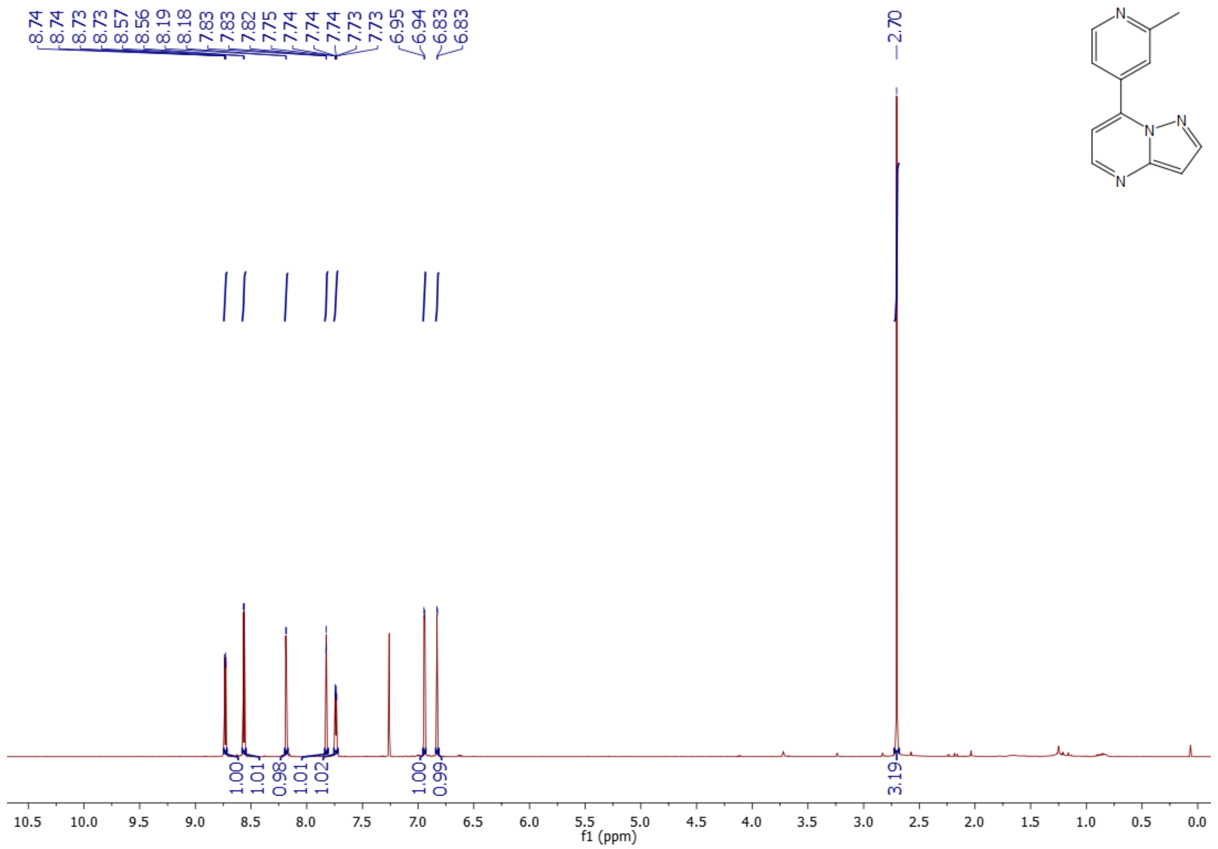

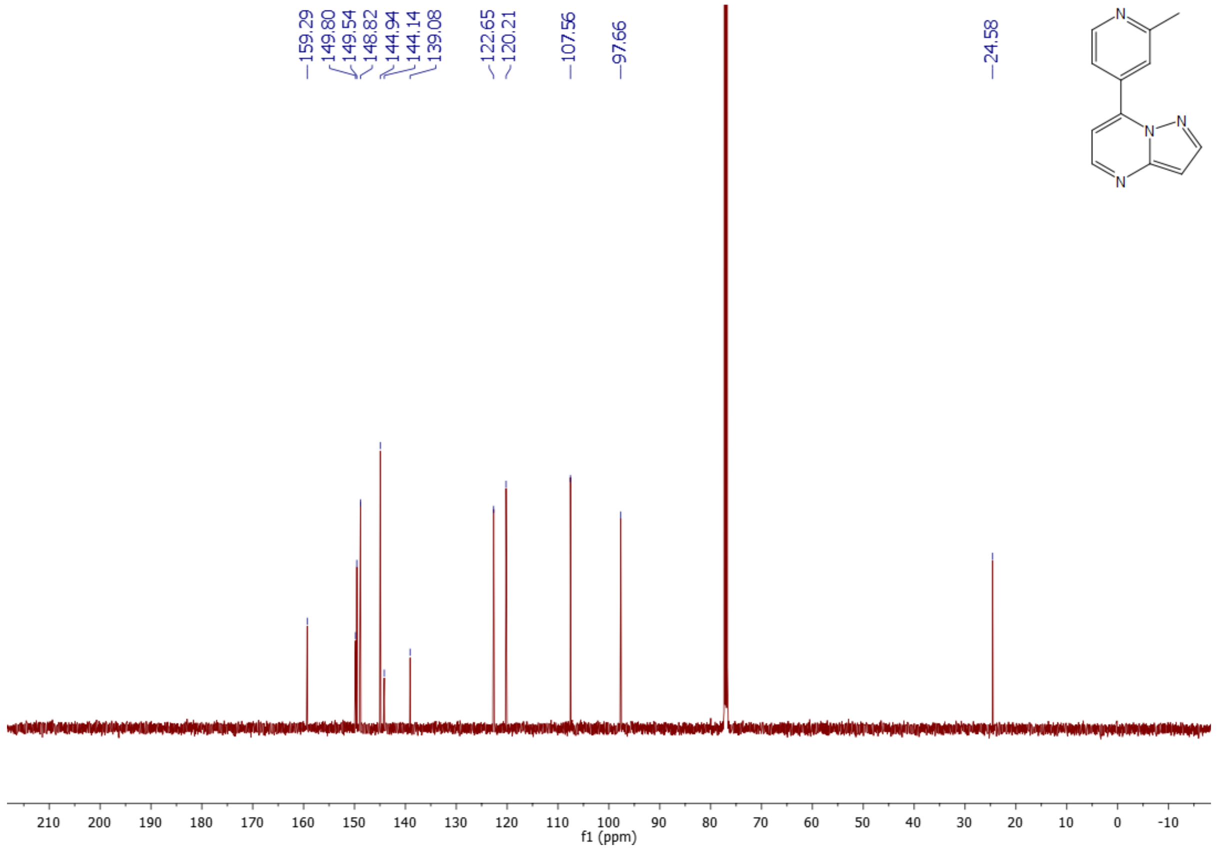

7-(2-fluoropyridin-4-yl)pyrazolo[1,5-a]pyrimidine, 2n

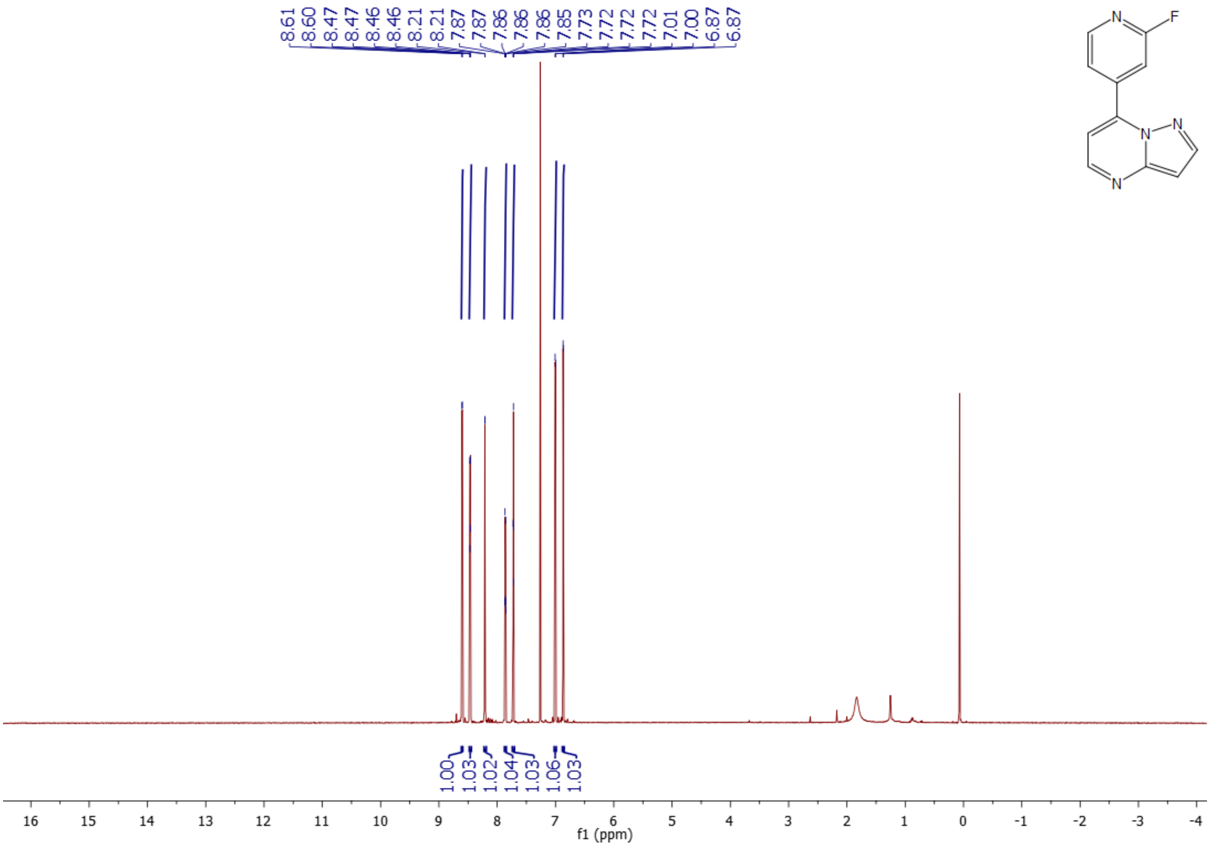

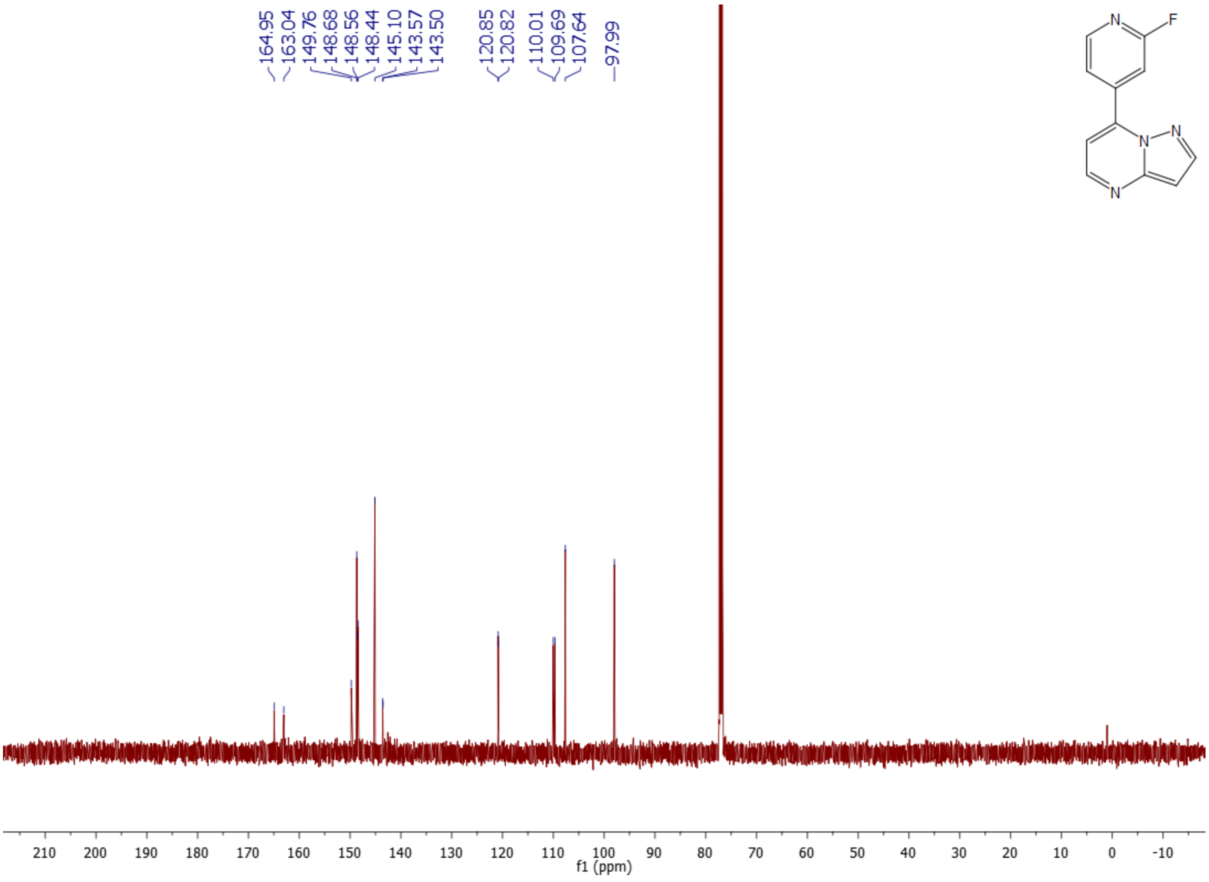

7-(pyridin-3-yl)pyrazolo[1,5-a]pyrimidine, 2o

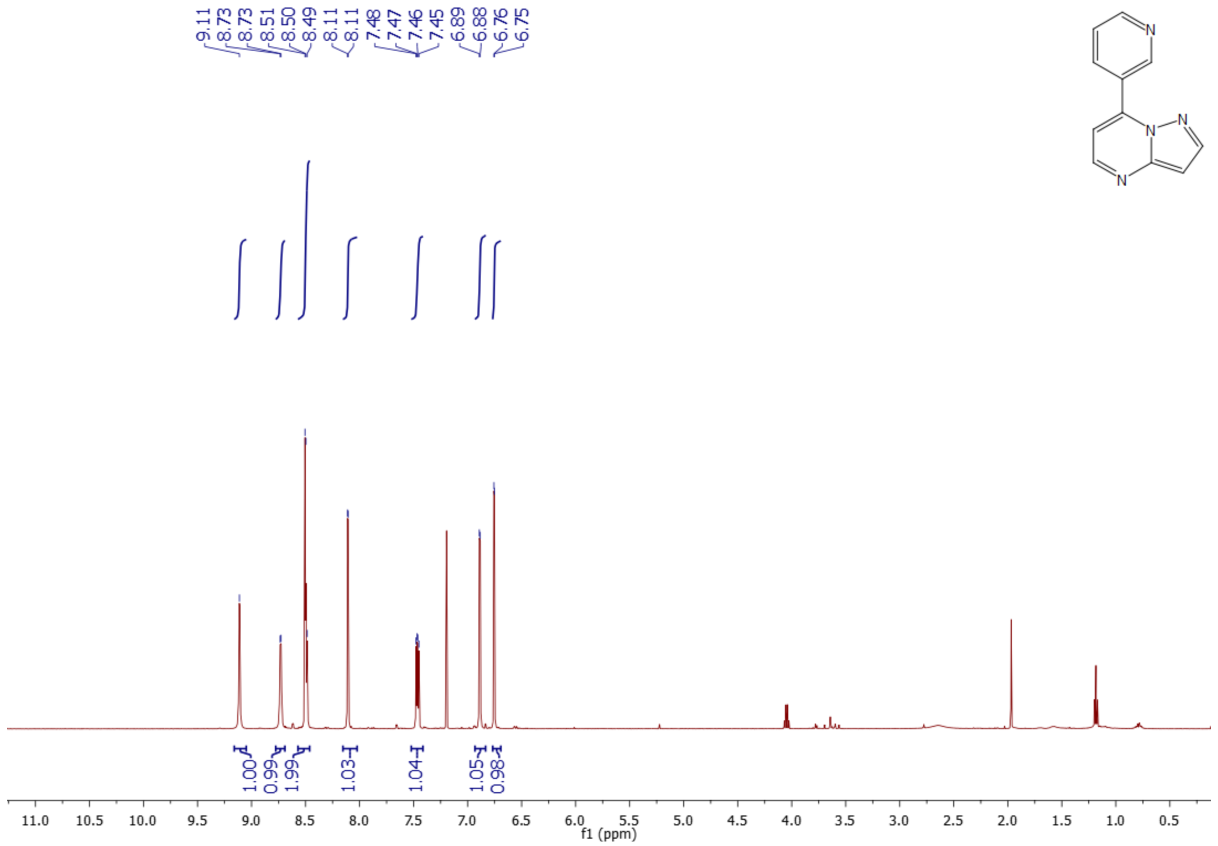

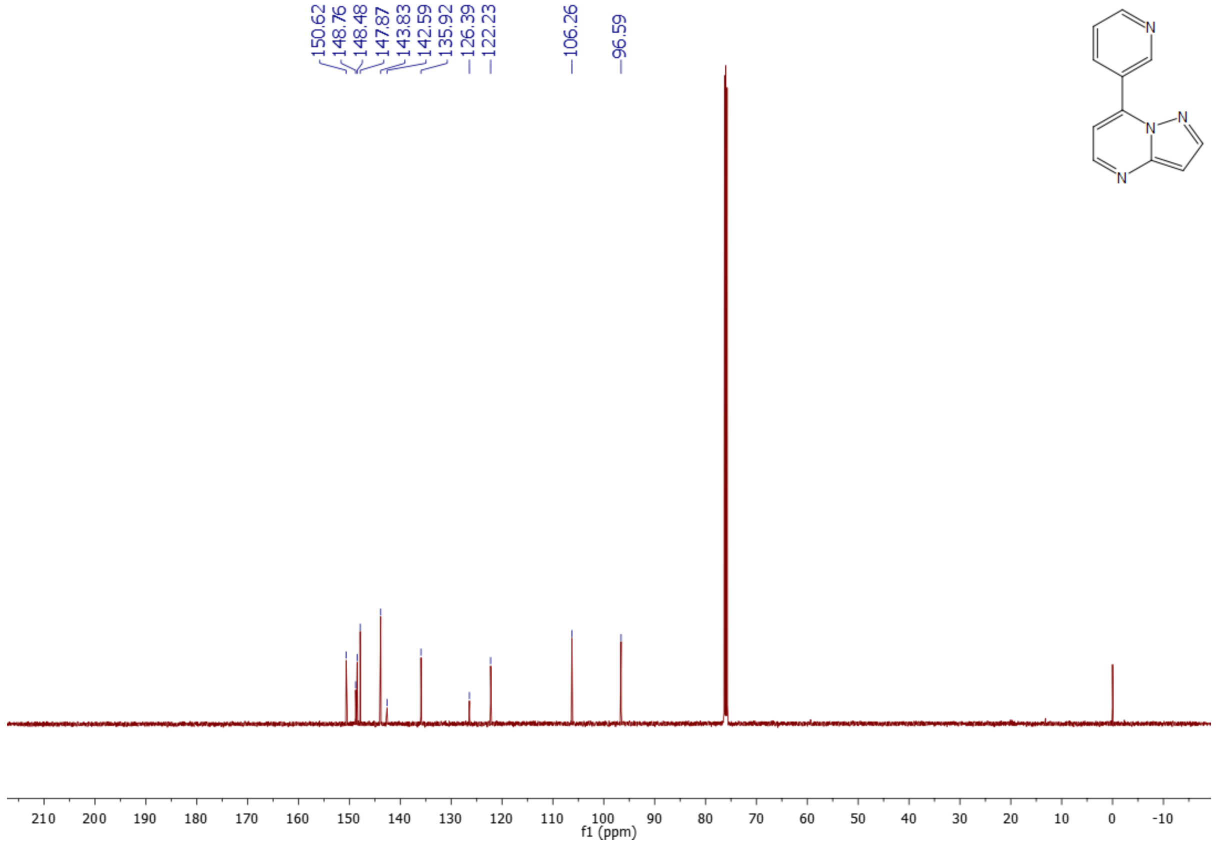

7-(pyrimidin-5-yl)pyrazolo[1,5-a]pyrimidine, 2p

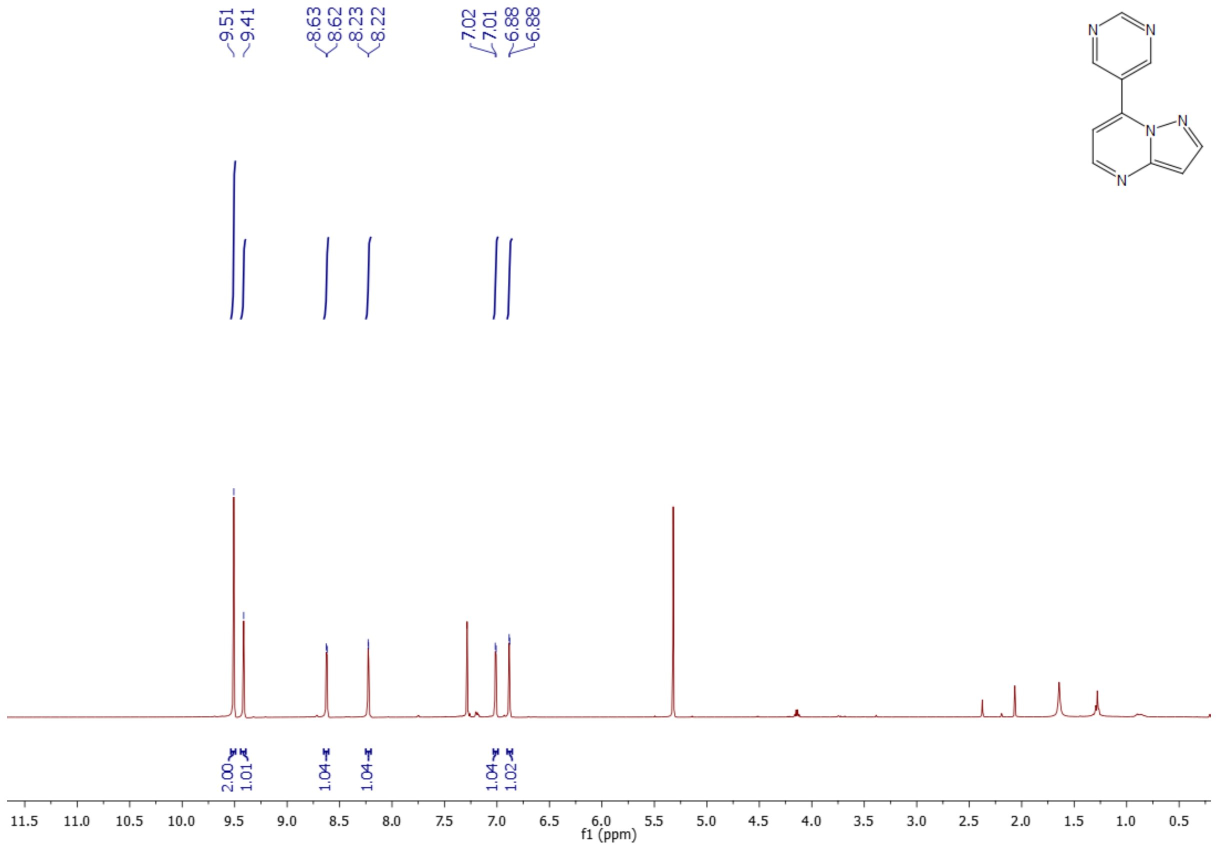

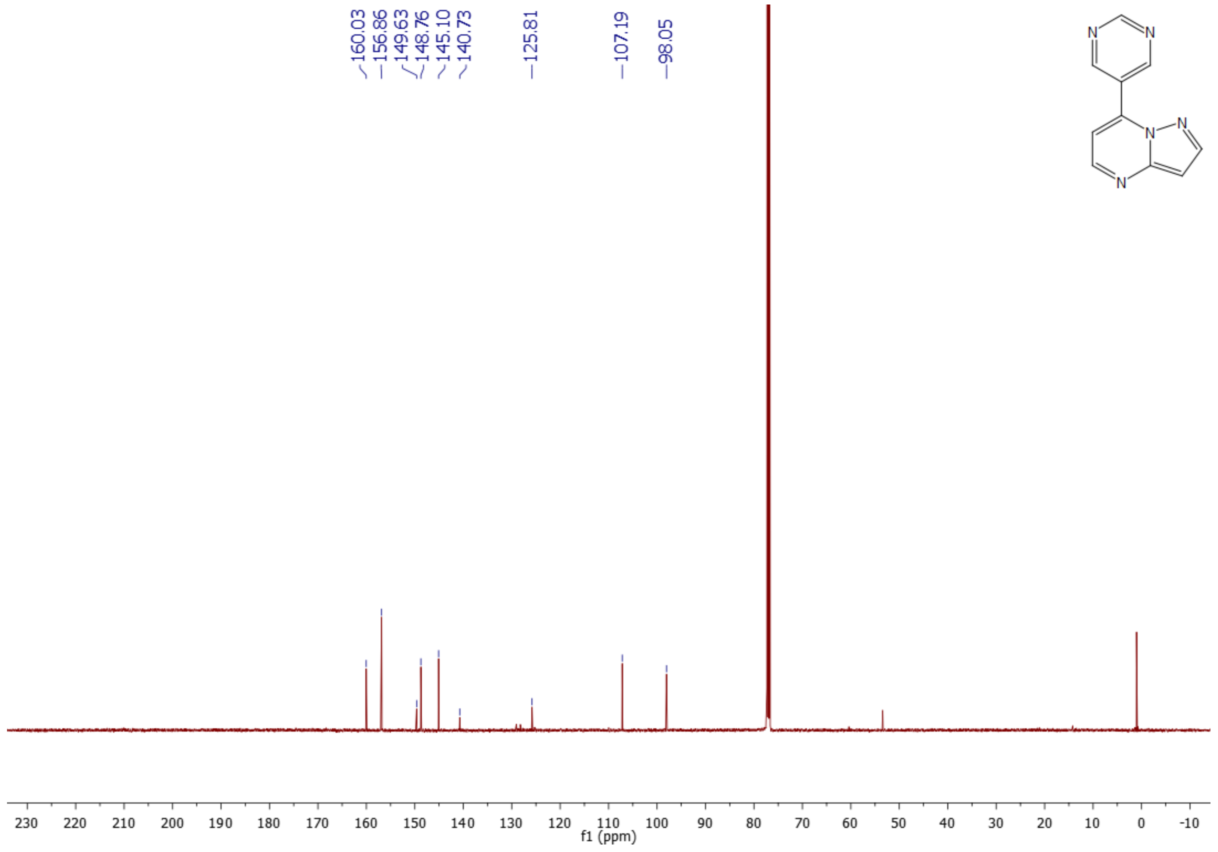

3-phenylpyrazolo[1,5-a]pyrimidine, 3a

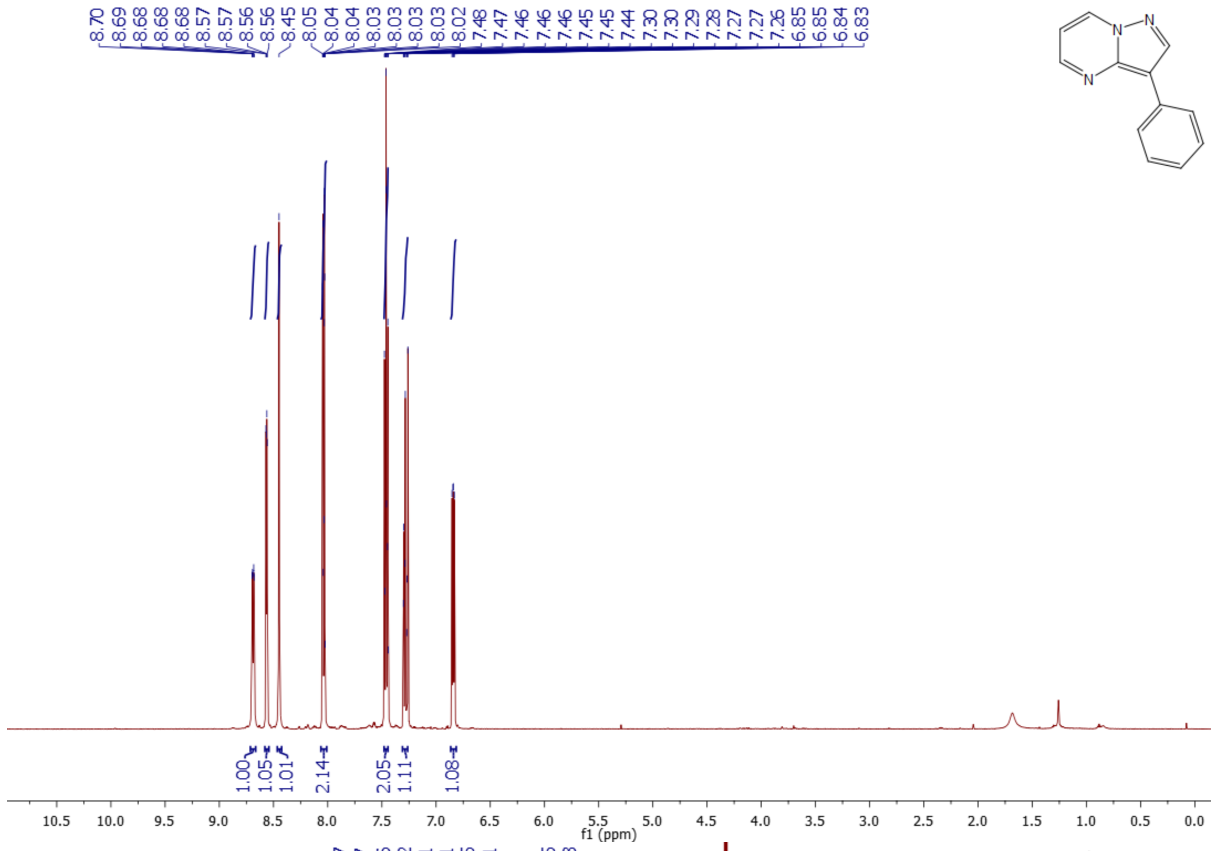

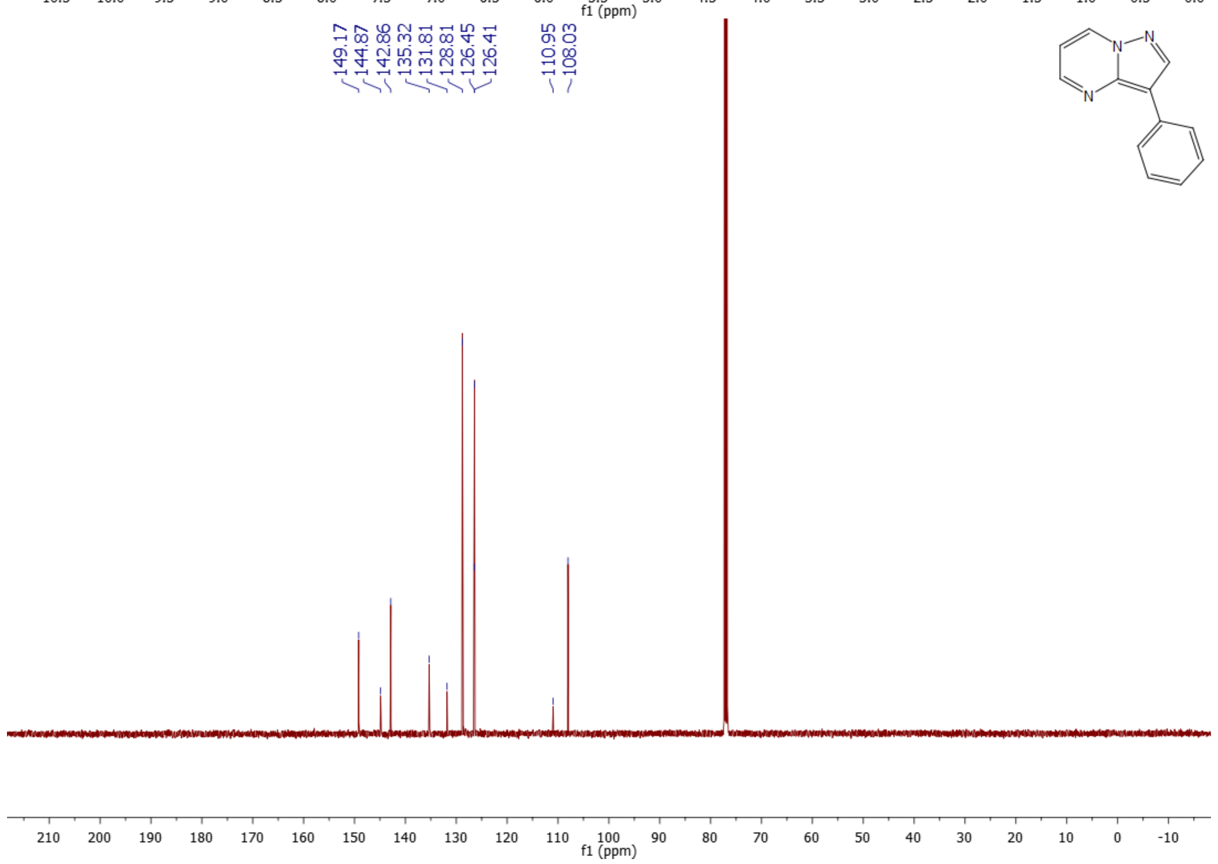

3-(p-tolyl)pyrazolo[1,5-a]pyrimidine, 3b

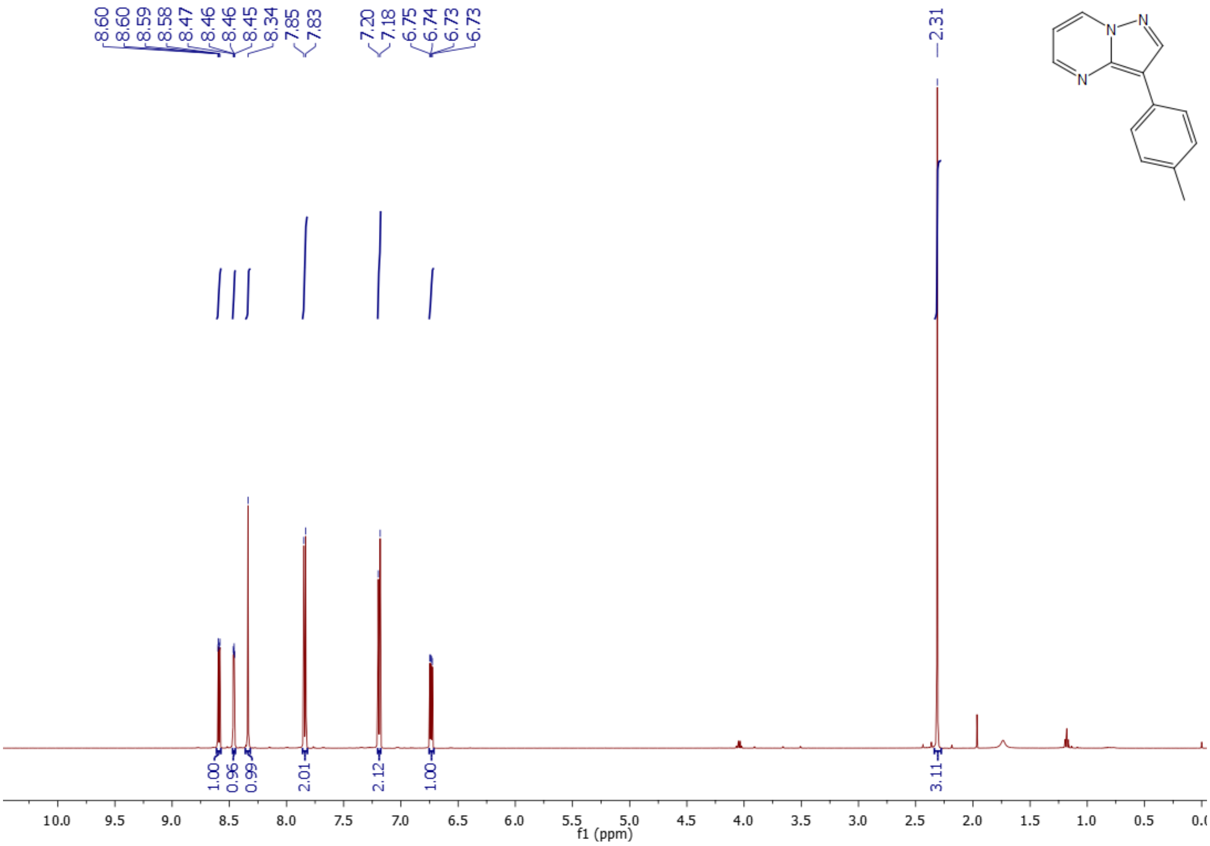

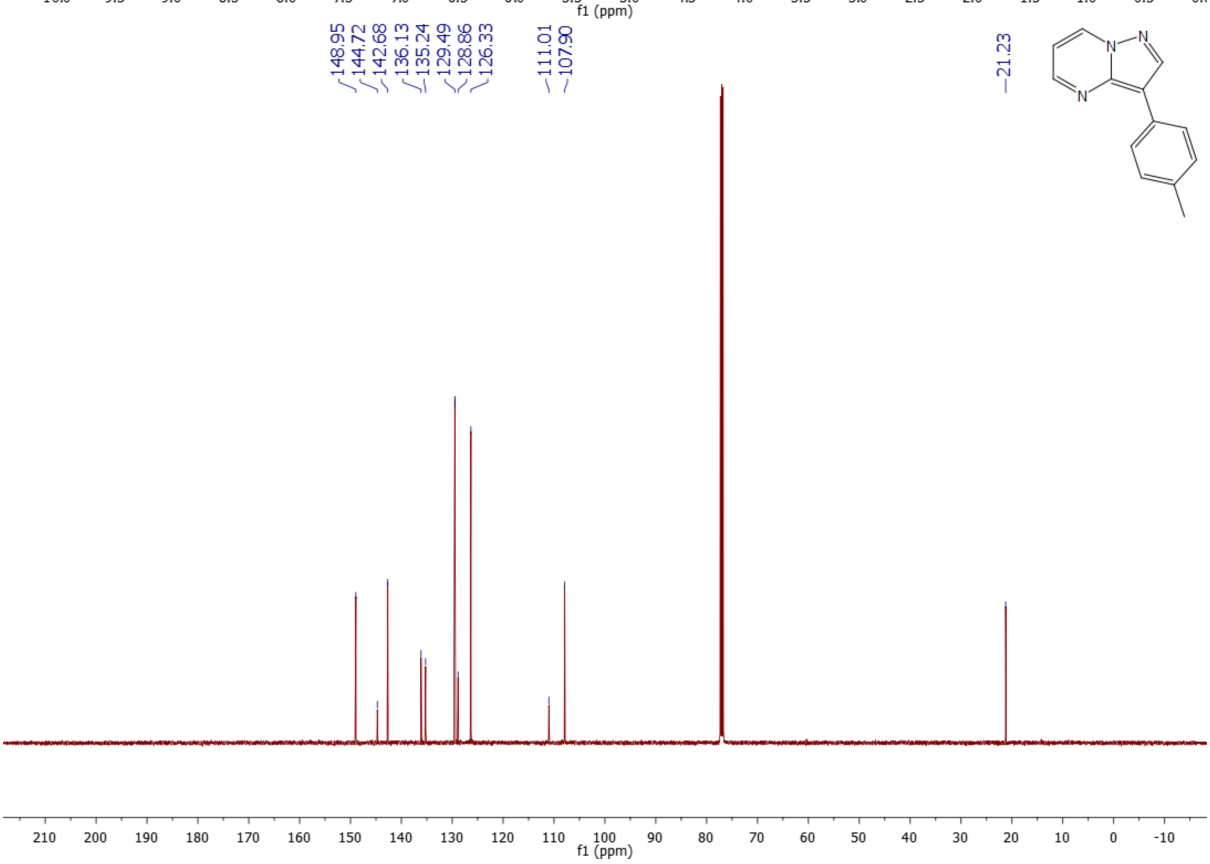

3-(4-methoxyphenyl)pyrazolo[1,5-a]pyrimidine, 3c

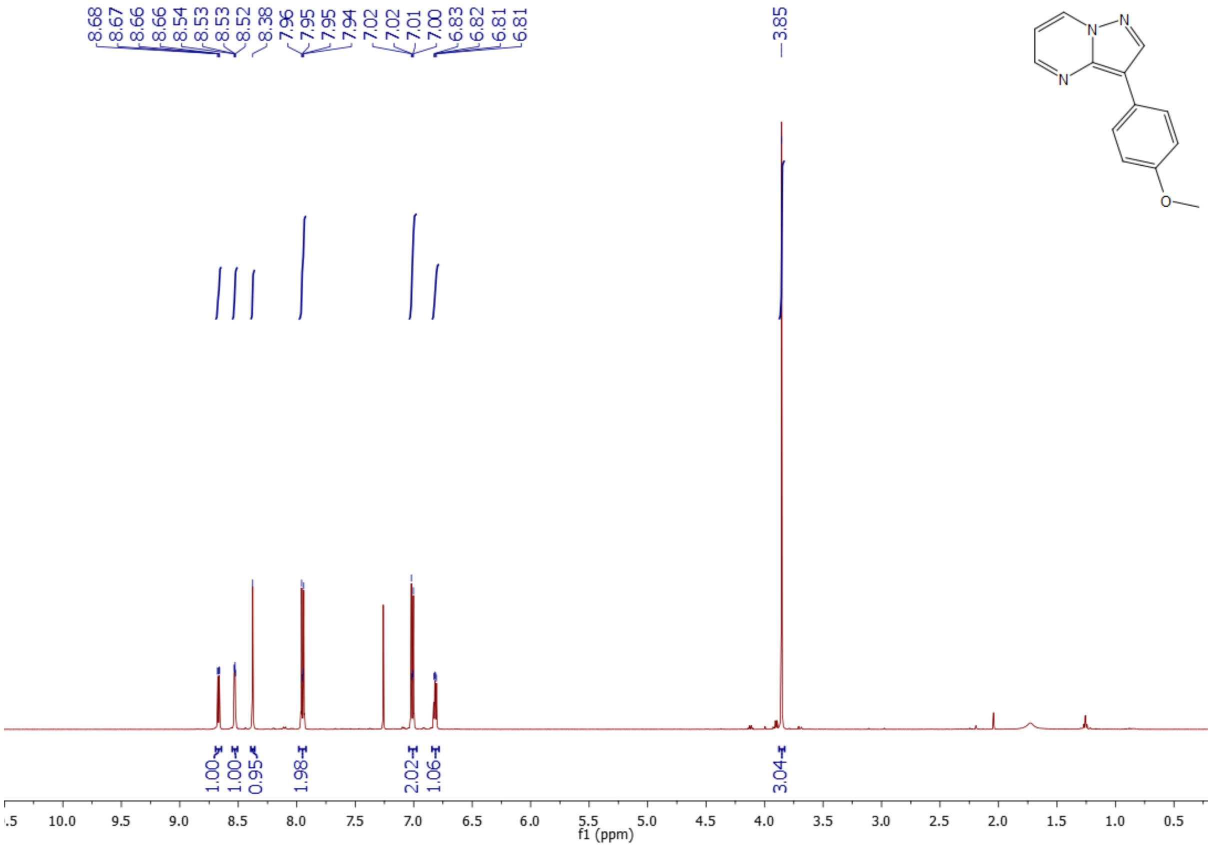

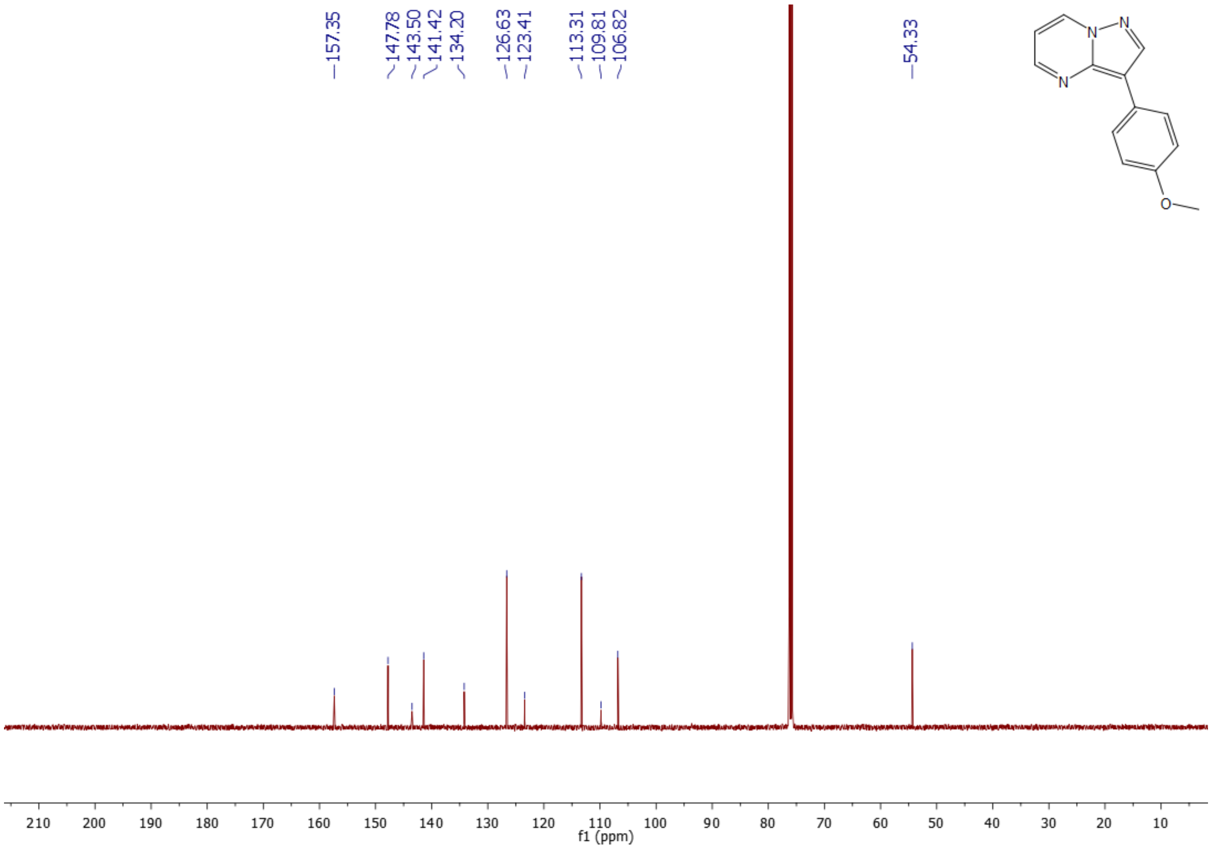

3-(4-fluorophenyl)pyrazolo[1,5-a]pyrimidine, 3d

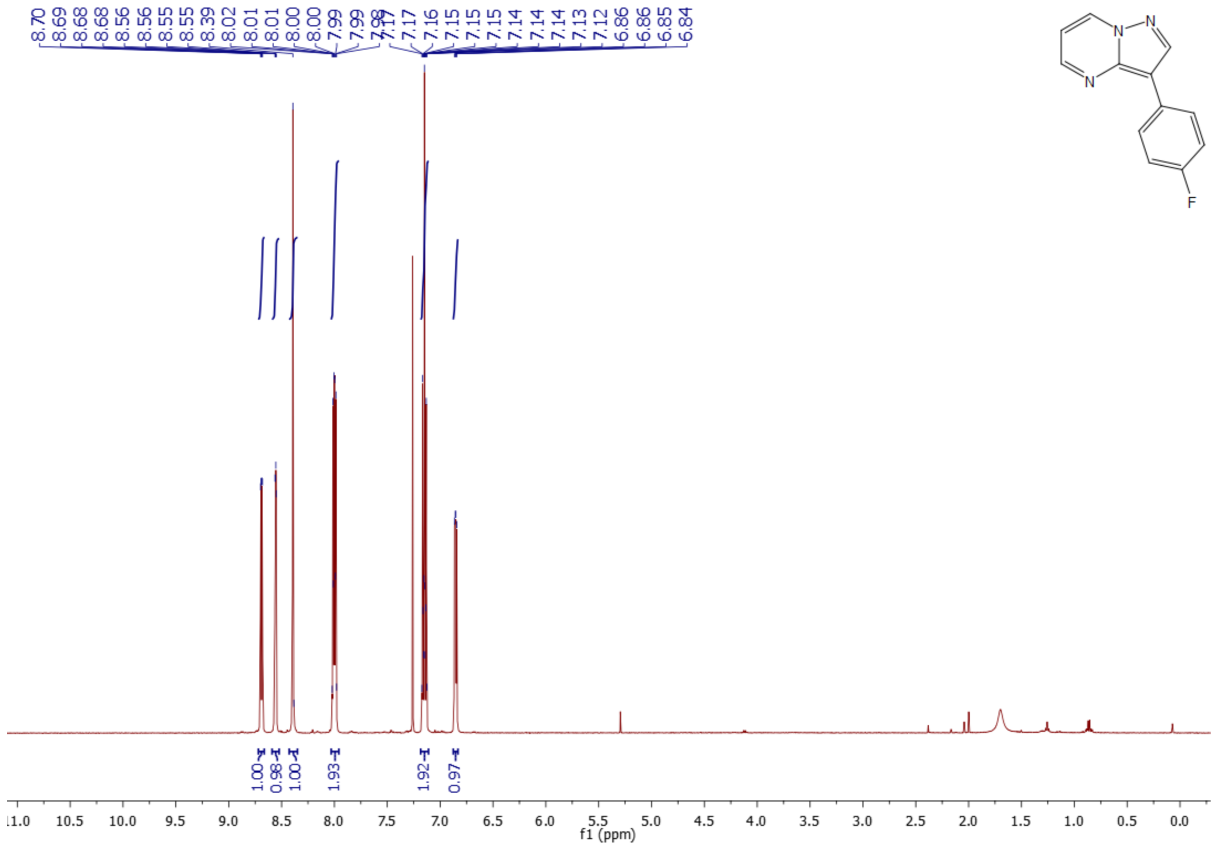

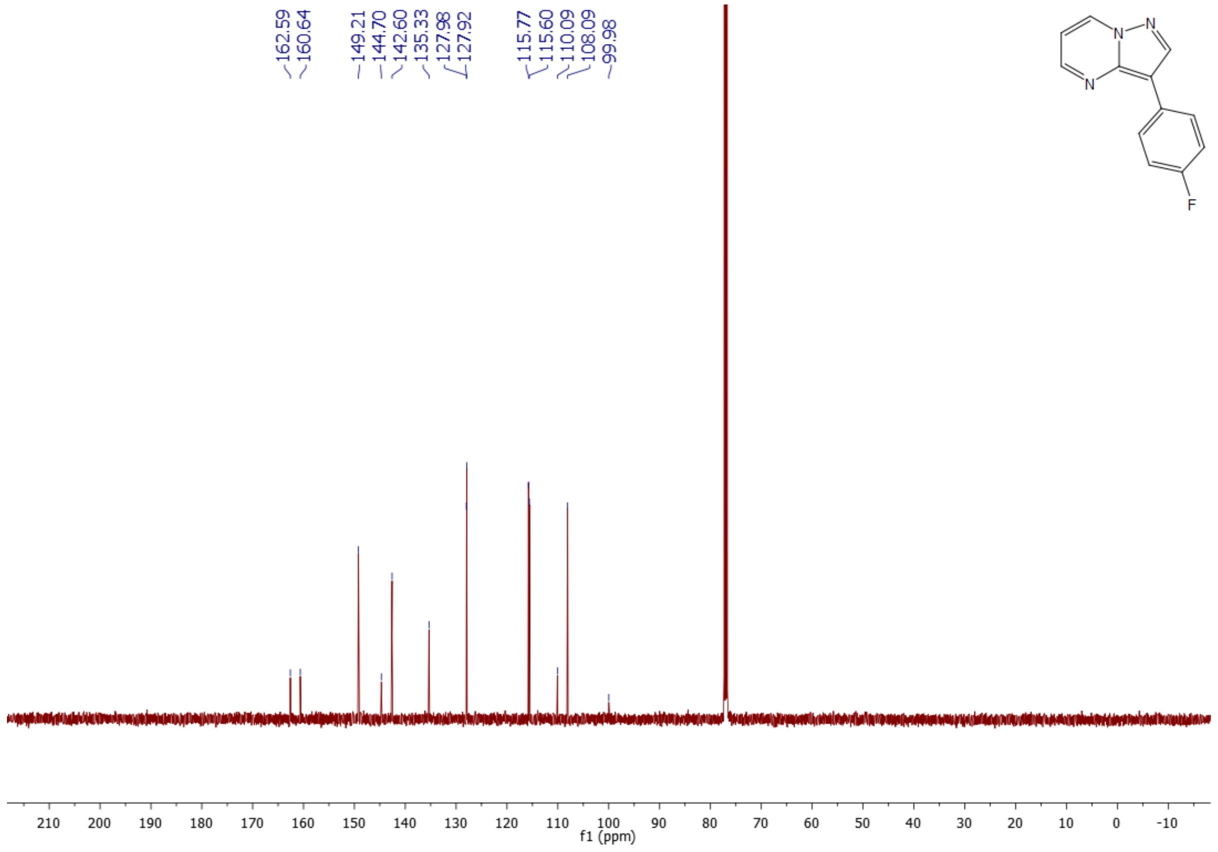

3-(4-(trifluoromethyl)phenyl)pyrazolo[1,5-a]pyrimidine, 3e

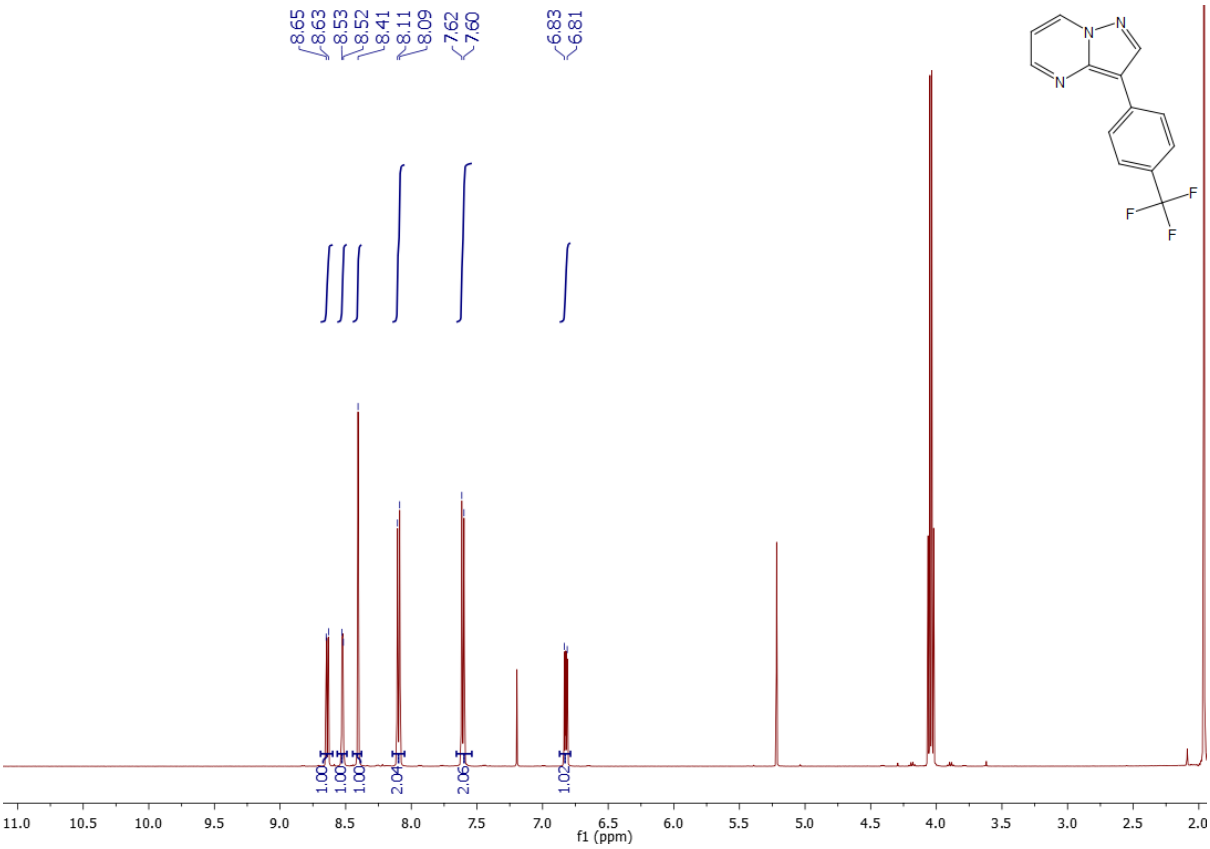

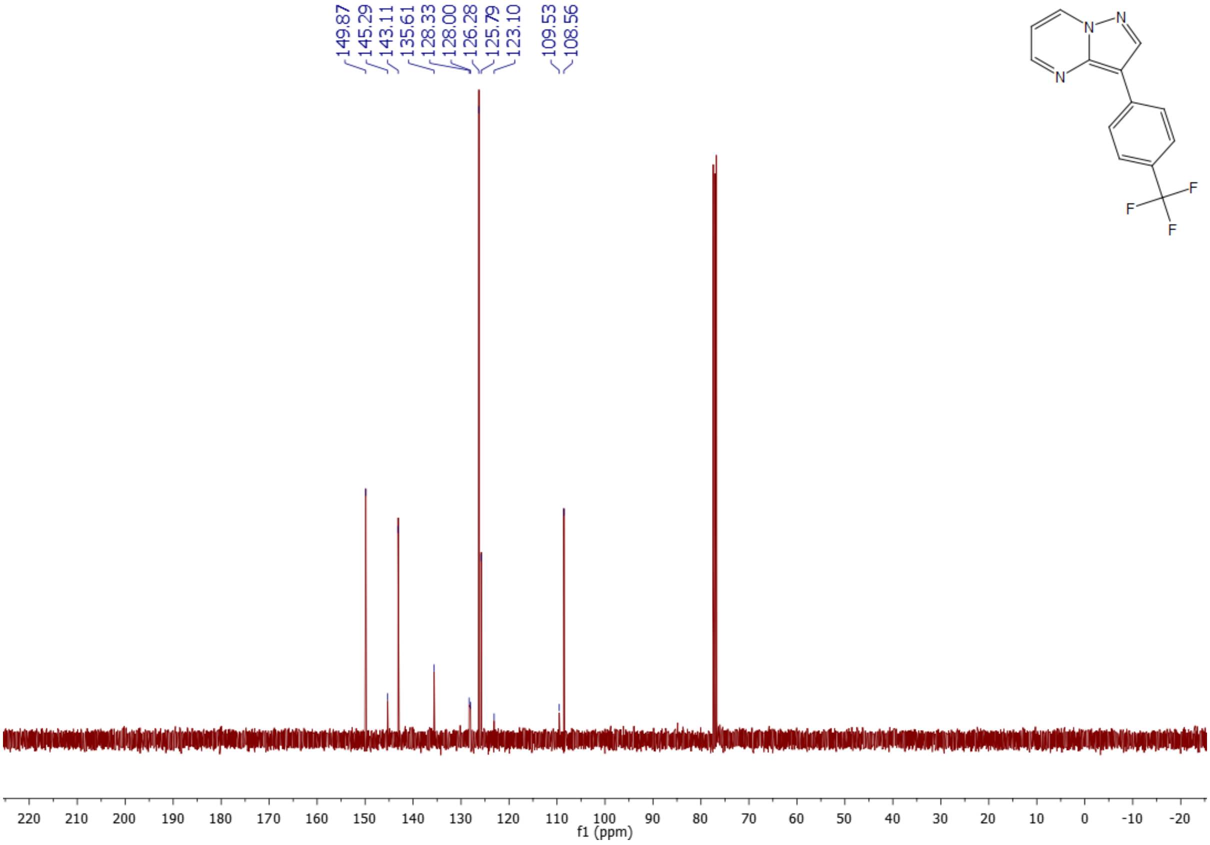

Methyl 4-(pyrazolo[1,5-a]pyrimidin-3-yl)benzoate, 3f

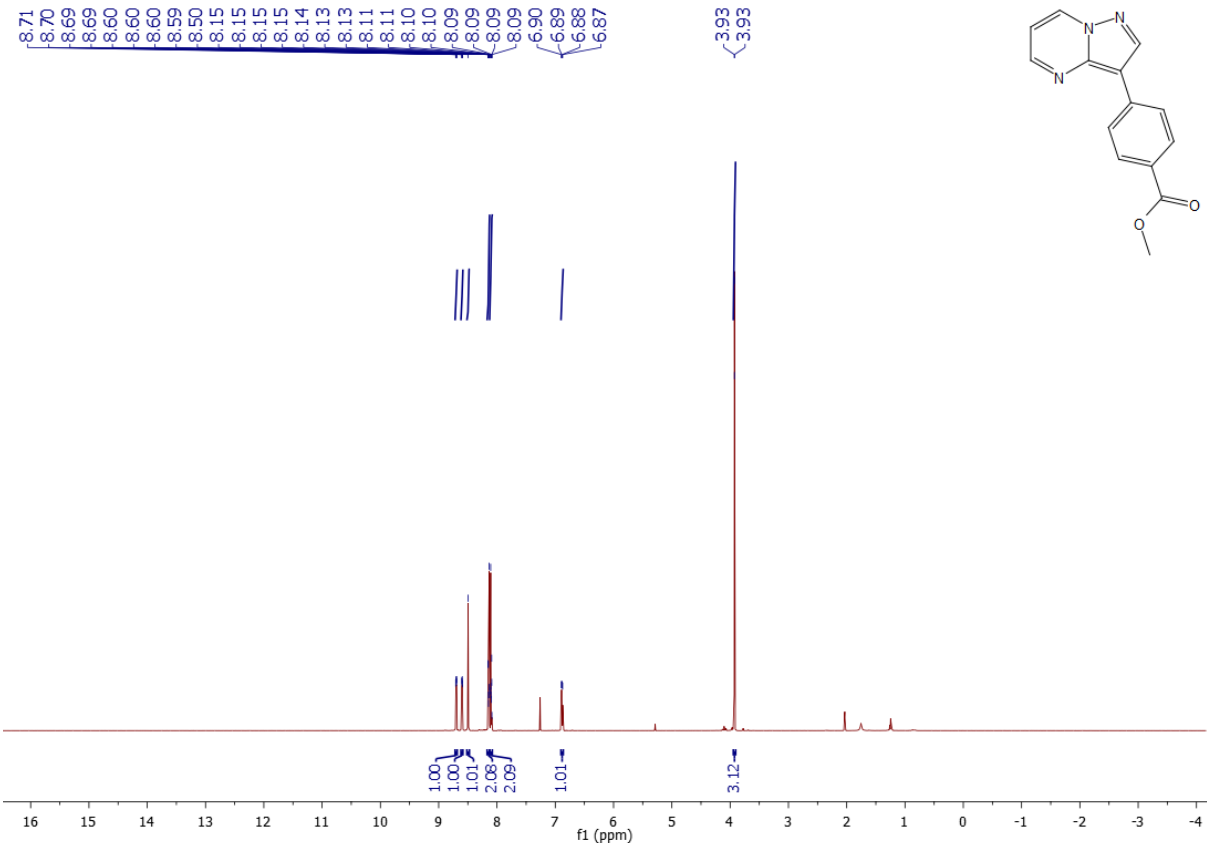

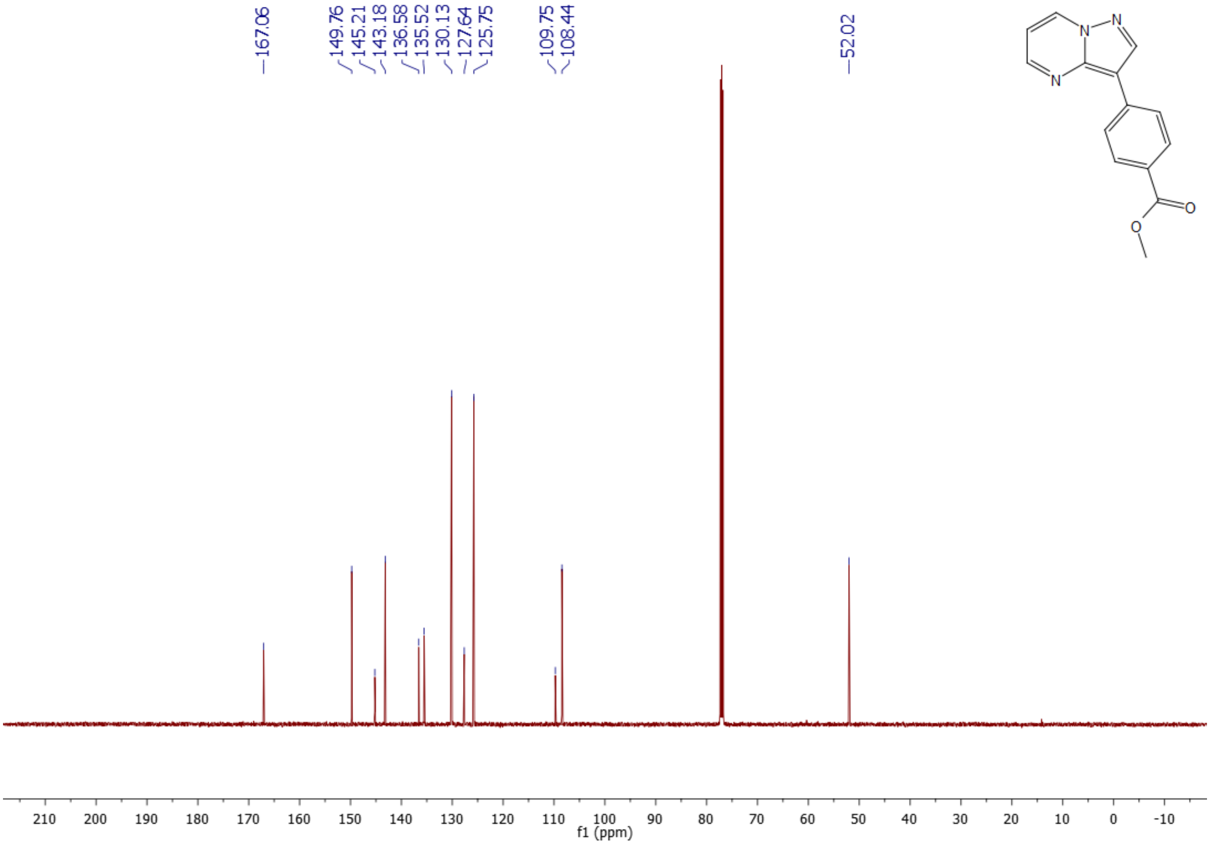

3-(4-chlorophenyl)pyrazolo[1,5-a]pyrimidine, 3g

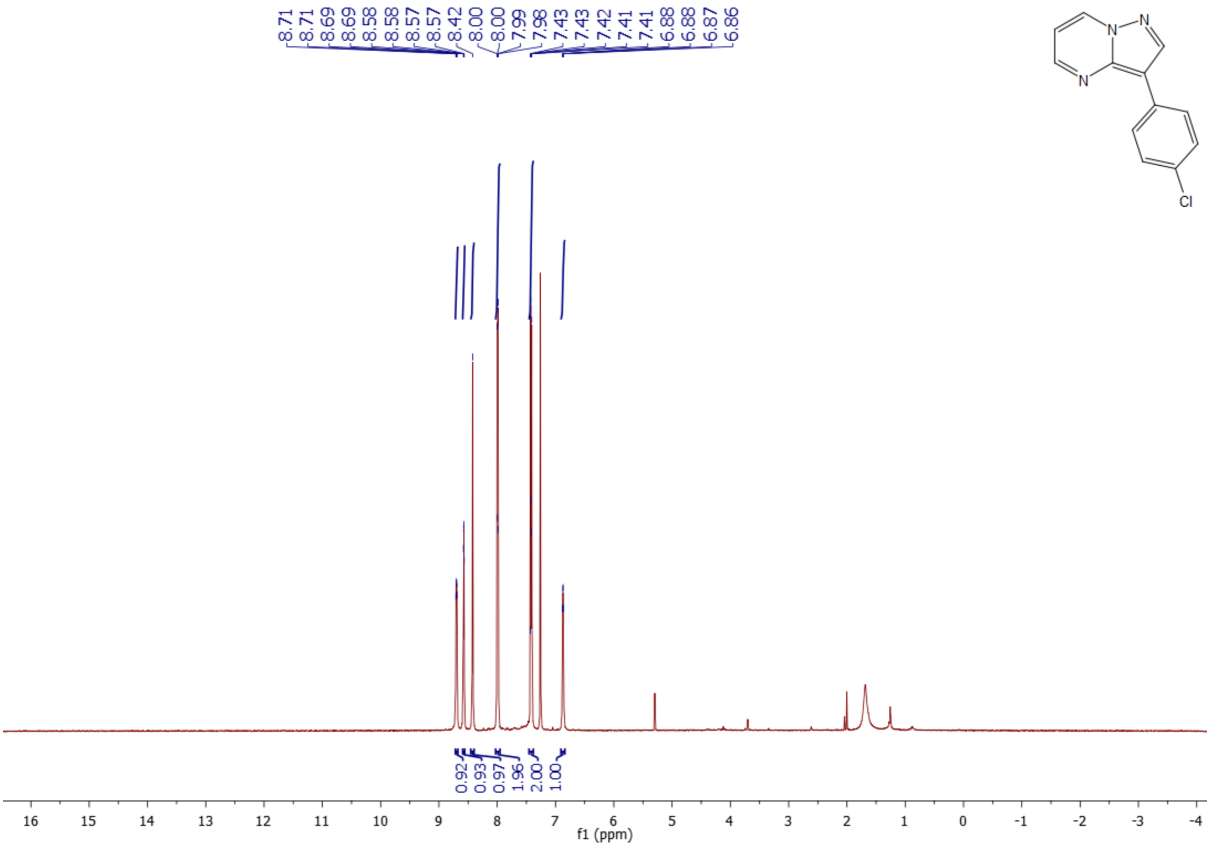

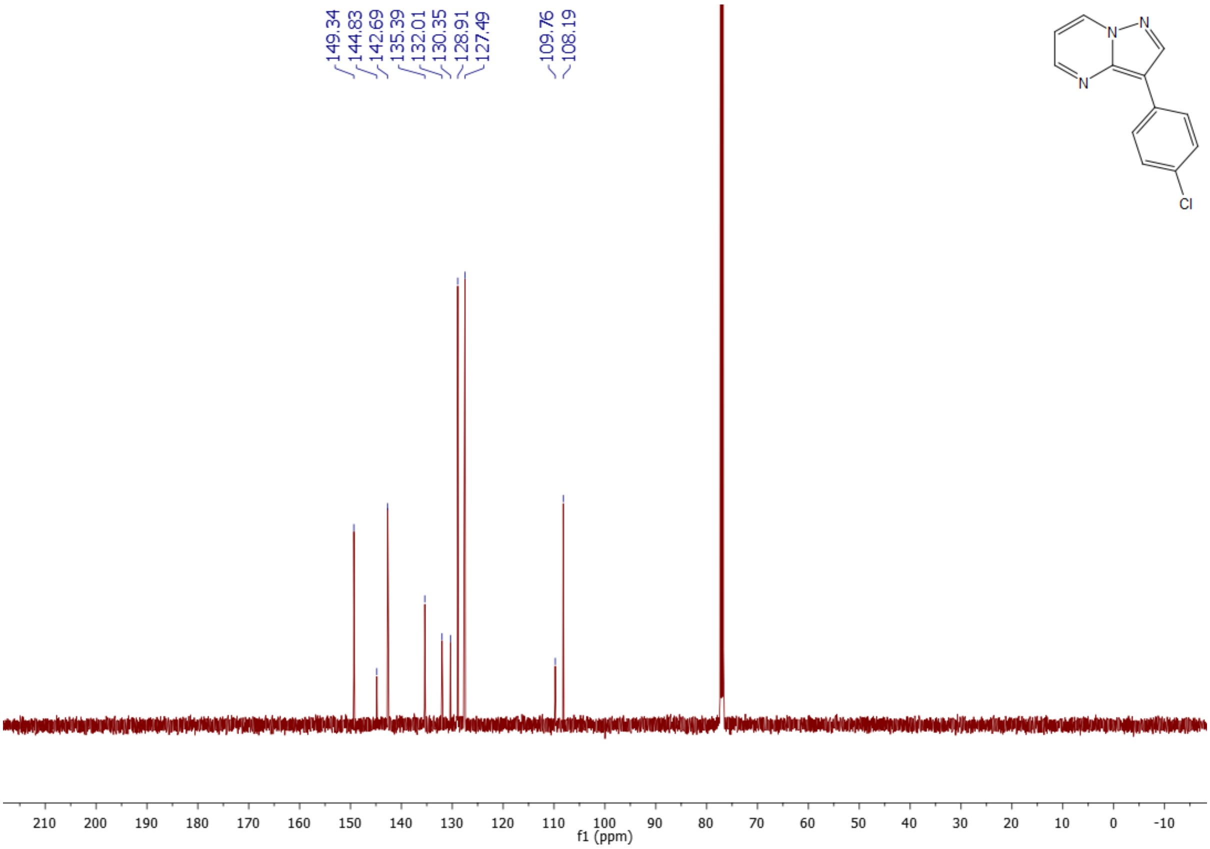

3-(o-tolyl)pyrazolo[1,5-a]pyrimidine, 3h

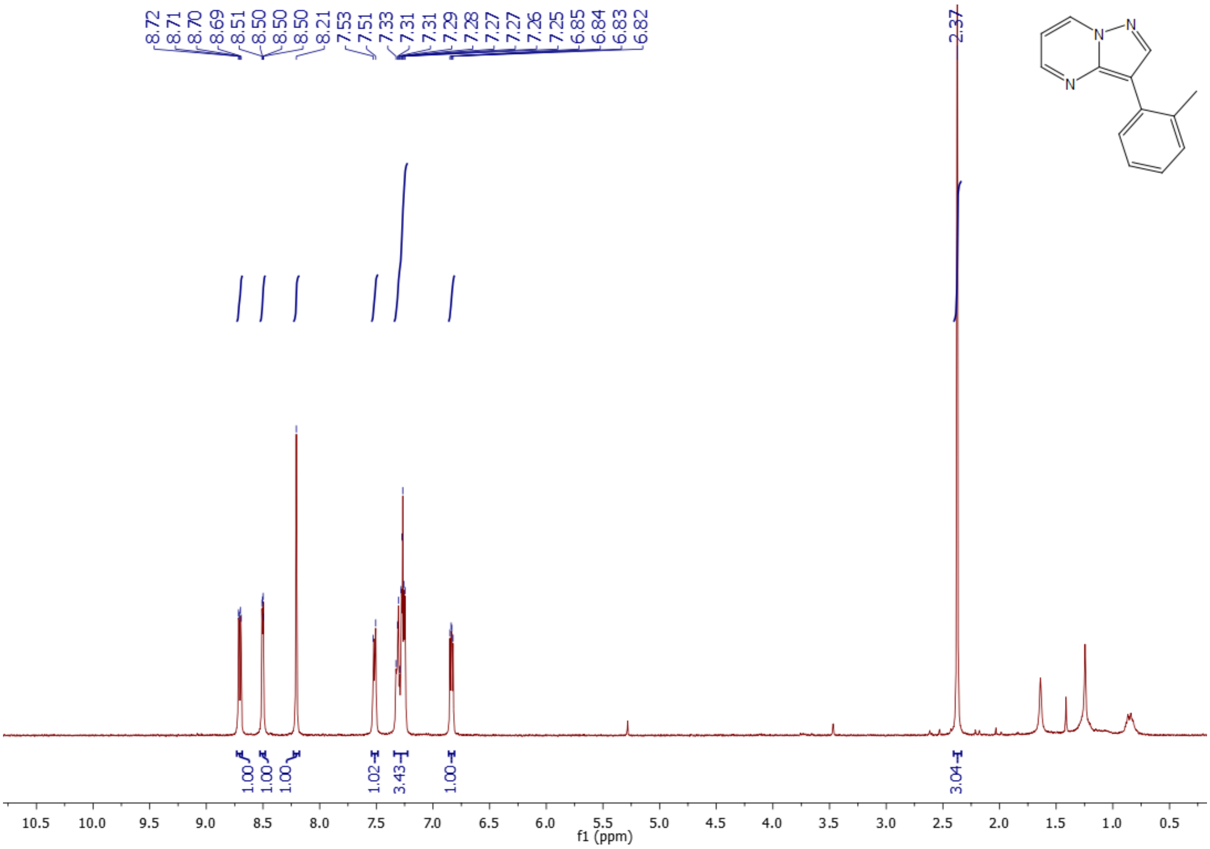

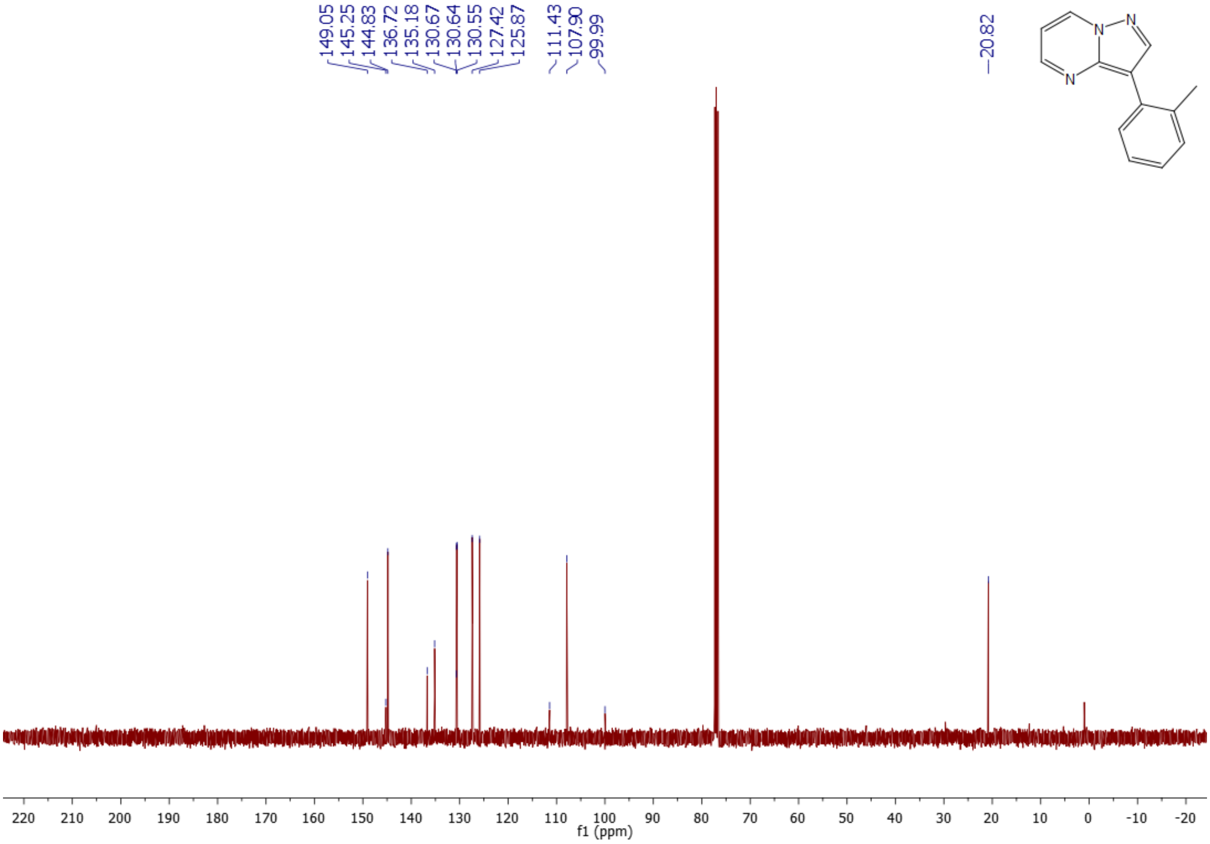

3-(2-nitrophenyl)pyrazolo[1,5-a]pyrimidine, 3i

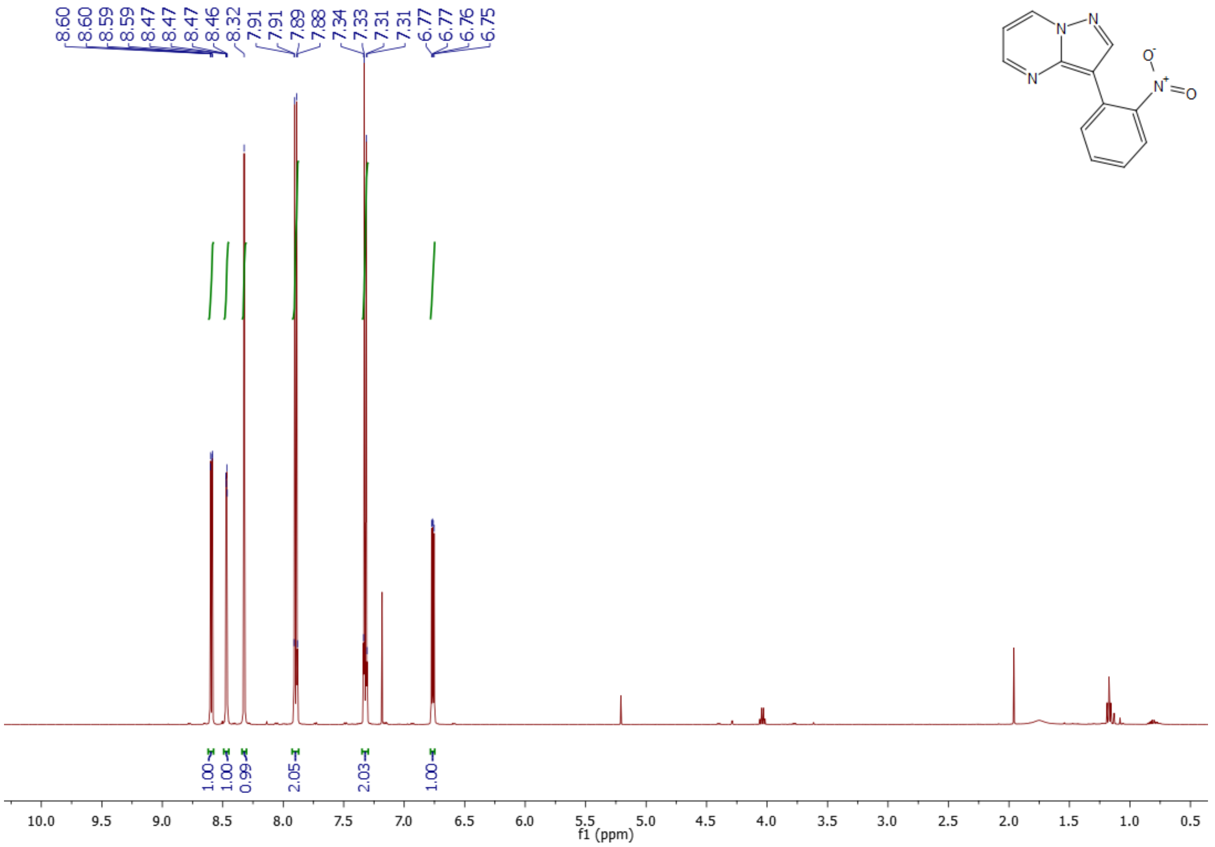

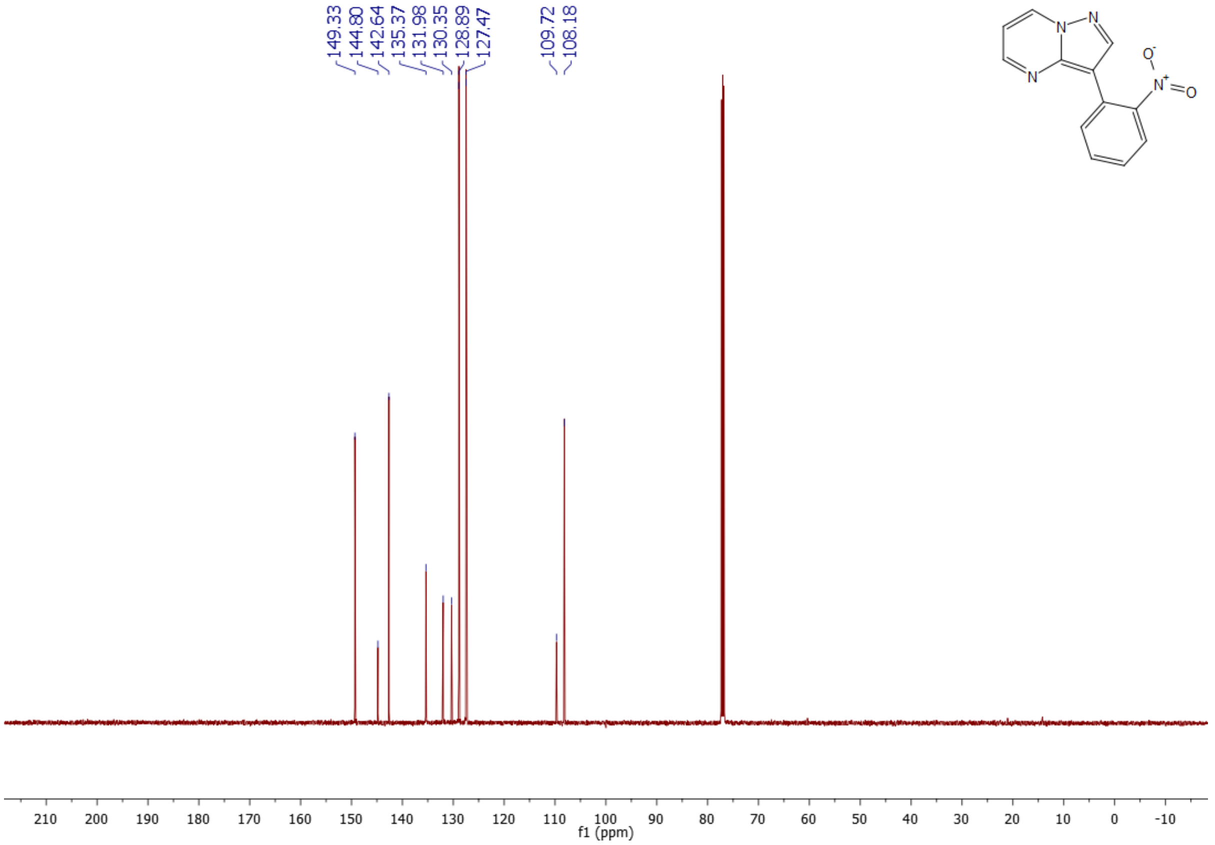

3-(m-tolyl)pyrazolo[1,5-a]pyrimidine, 3j

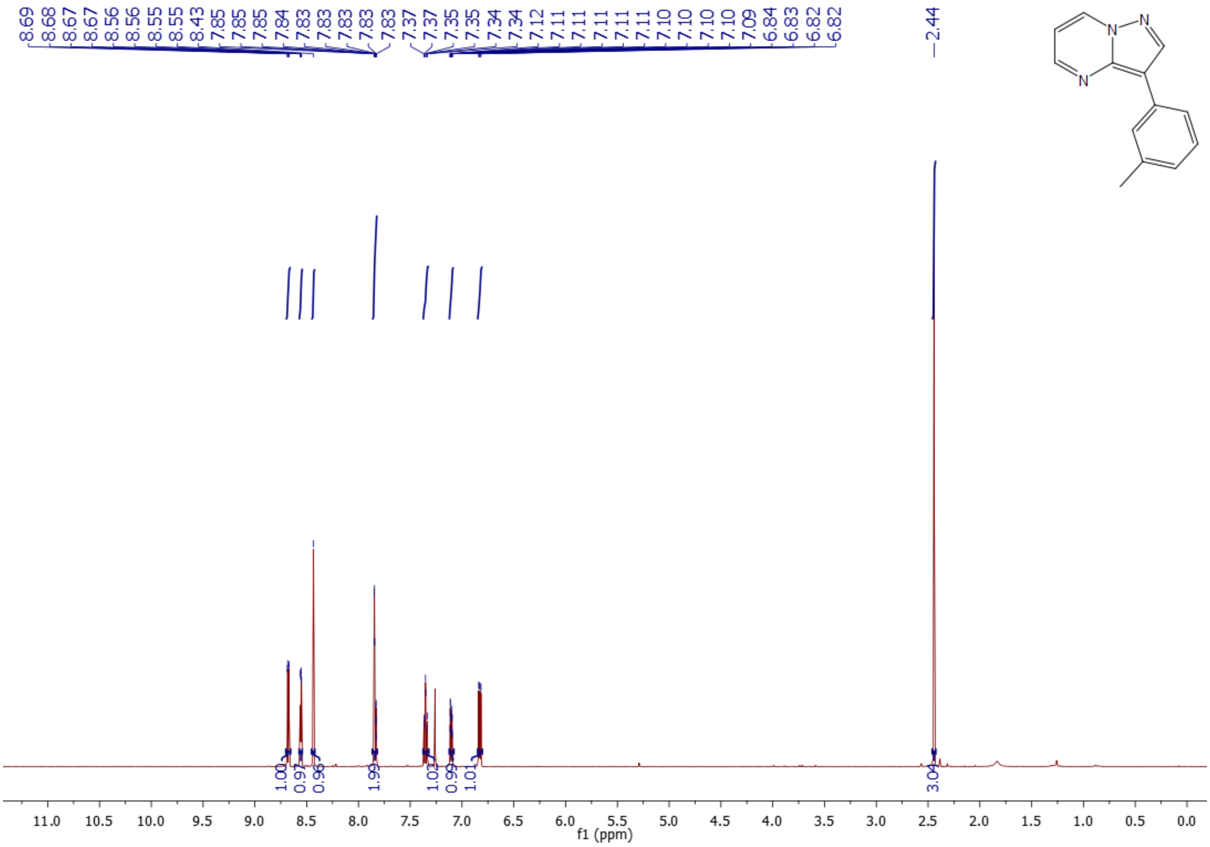

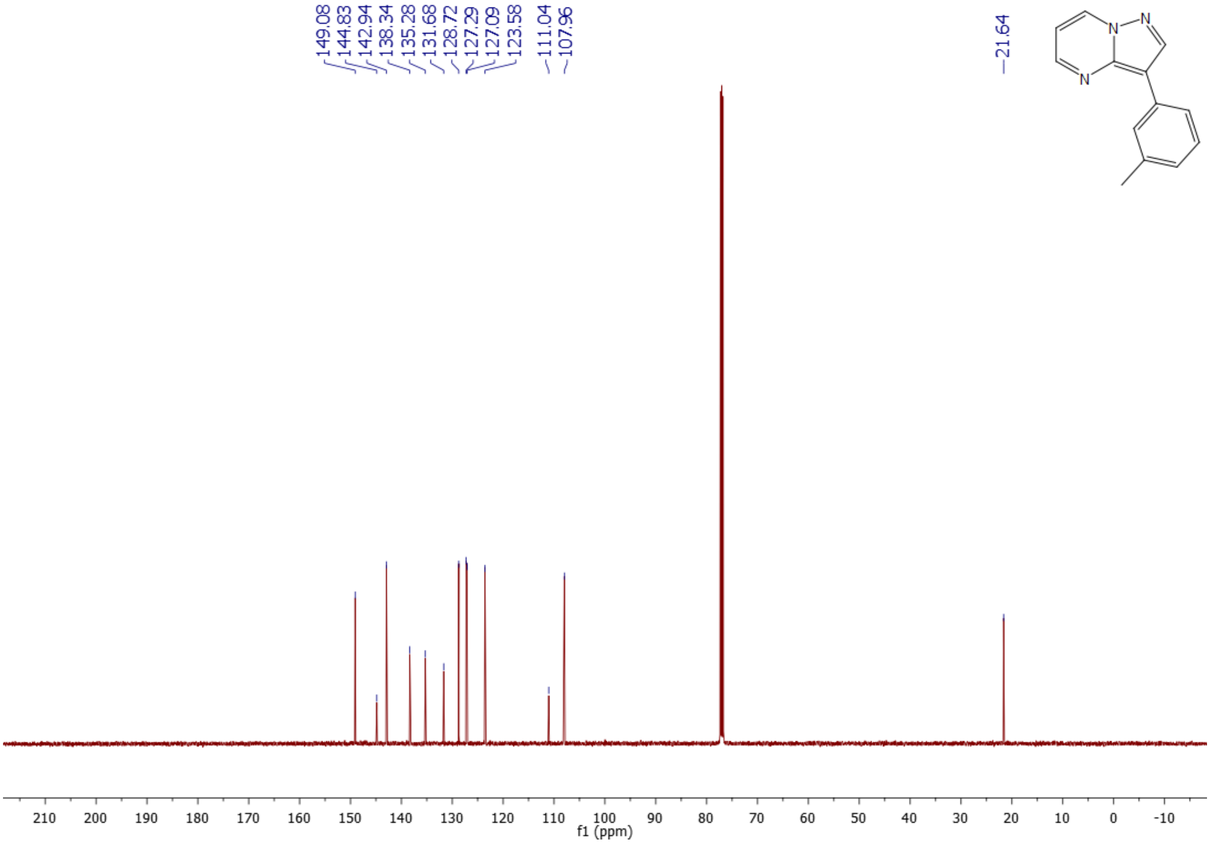

3-(pyrazolo[1,5-a]pyrimidin-3-yl)benzonitrile, 3k

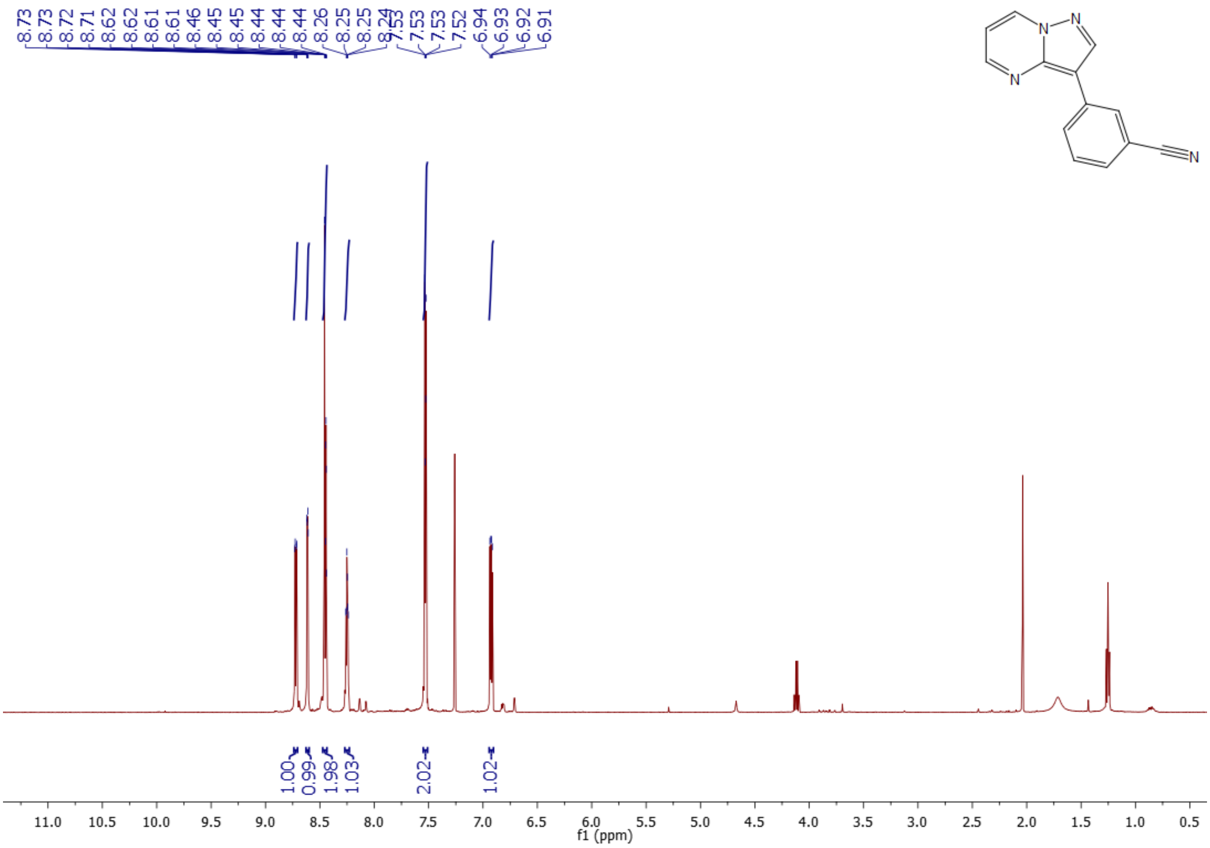
8.73
8.73
8.72
8.71
8.62
8.62
8.61
8.61
8.46
8.45
8.45
8.44
8.44
8.44
8.26
8.25
8.25
8.24
7.53
7.53
7.53
7.52
6.94
6.93
6.92
6.91
N
N
N
N
1.00
0.99
1.98
1.03
2.02
1.02
11.0 10.5 10.0 9.5 9.0 8.5 8.0 7.5 7.0 6.5 6.0 5.5 5.0 4.5 4.0 3.5 3.0 2.5 2.0 1.5 1.0 0.5
f1 (ppm)

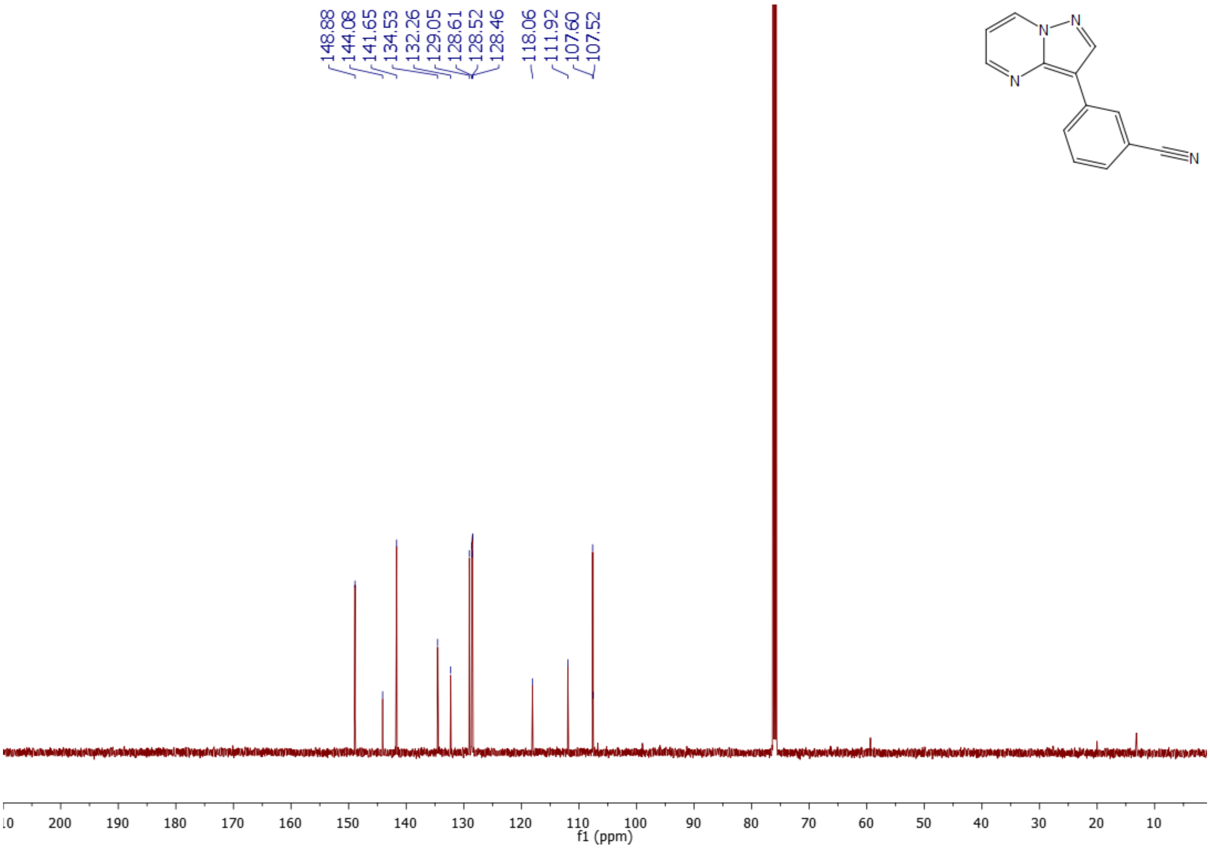
148.88
144.08
141.65
134.53
132.26
129.05
128.61
128.52
128.46
118.06
111.92
107.60
107.52
N
N
N
N
200 190 180 170 160 150 140 130 120 110 100 90 80 70 60 50 40 30 20 10
f1 (ppm)

3-(2,6-dimethylphenyl)pyrazolo[1,5-a]pyrimidine, 3l

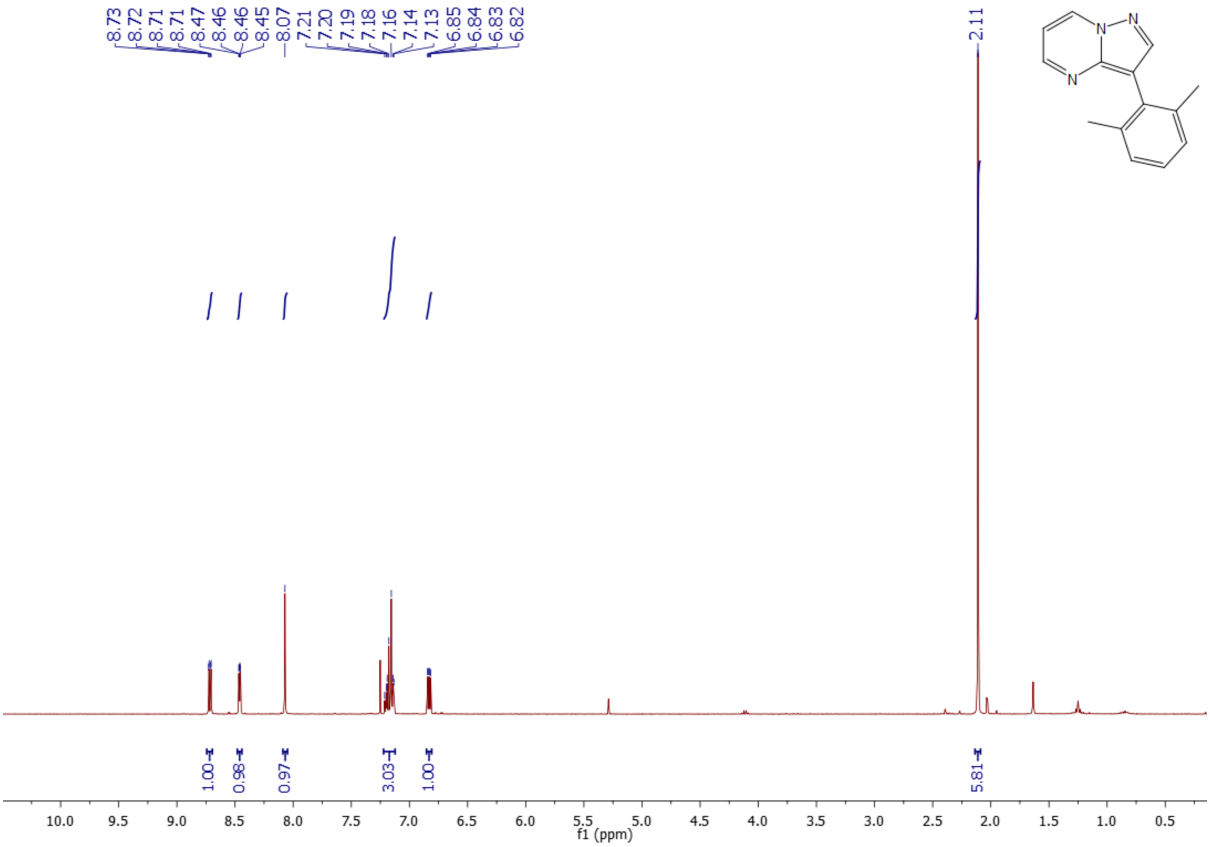

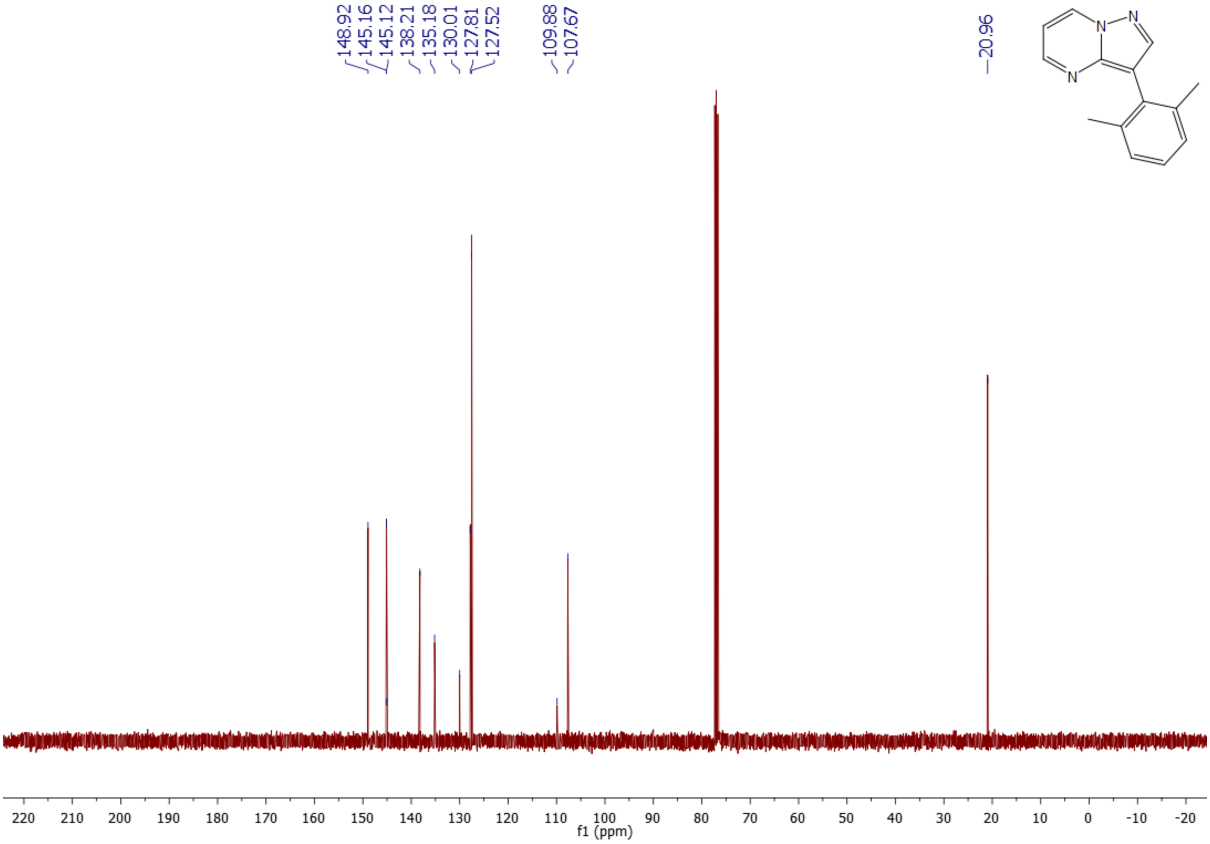

3-(naphthalen-2-yl)pyrazolo[1,5-a]pyrimidine, 3m

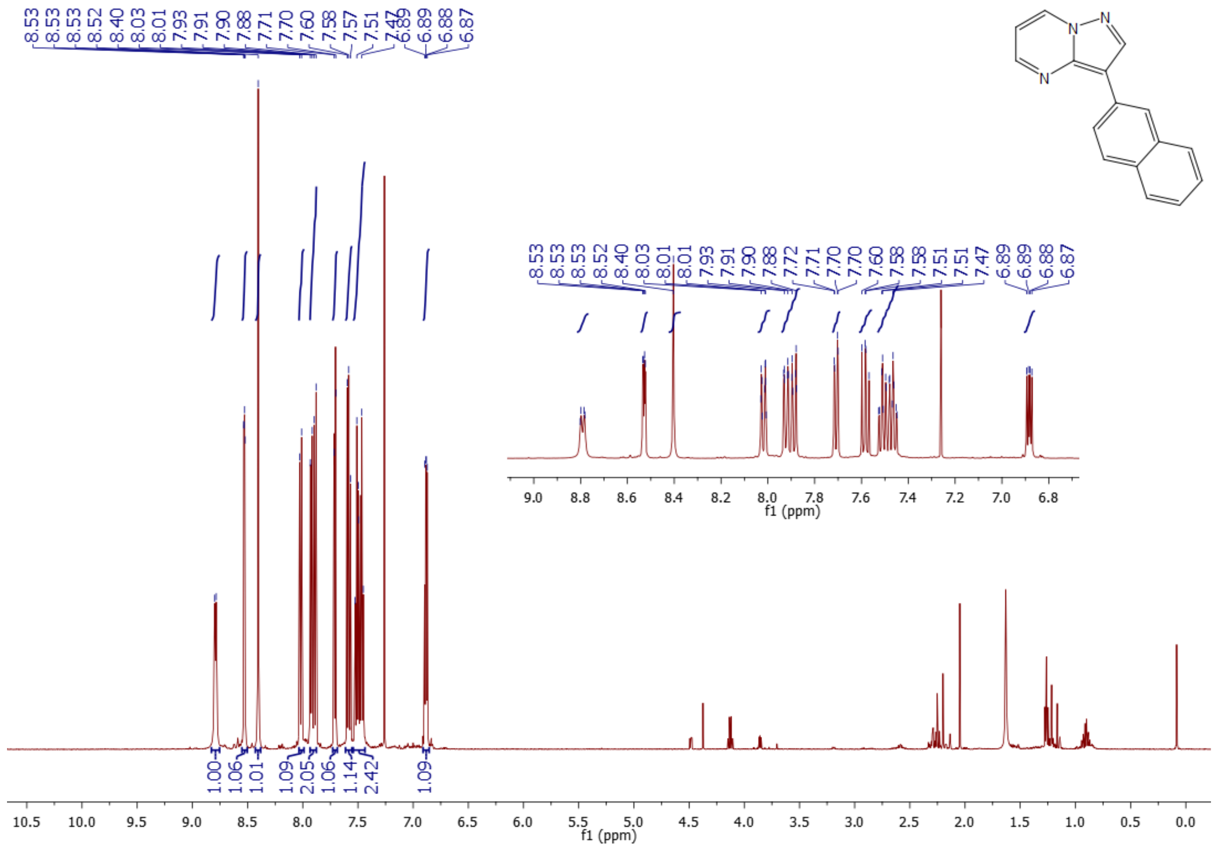

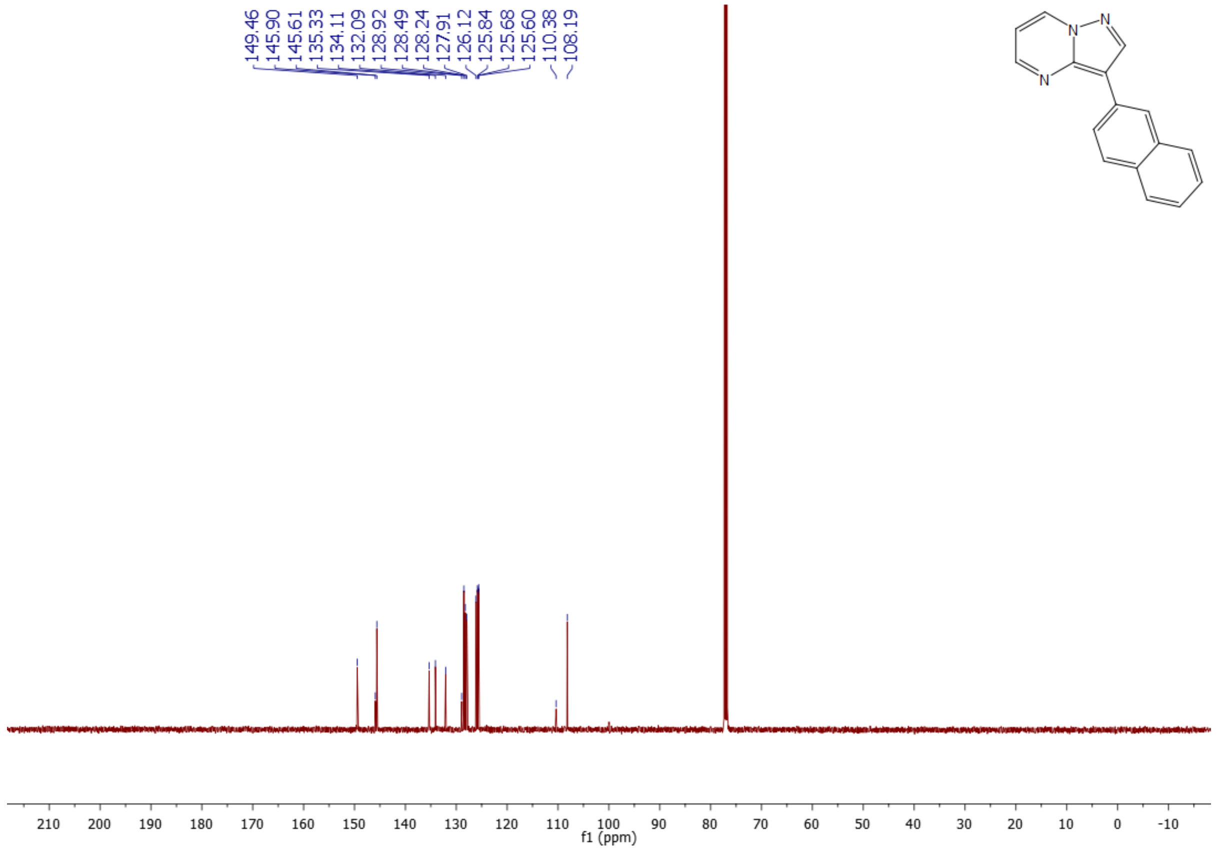

3-(2-methylpyridin-4-yl)pyrazolo[1,5-a]pyrimidine, 3n

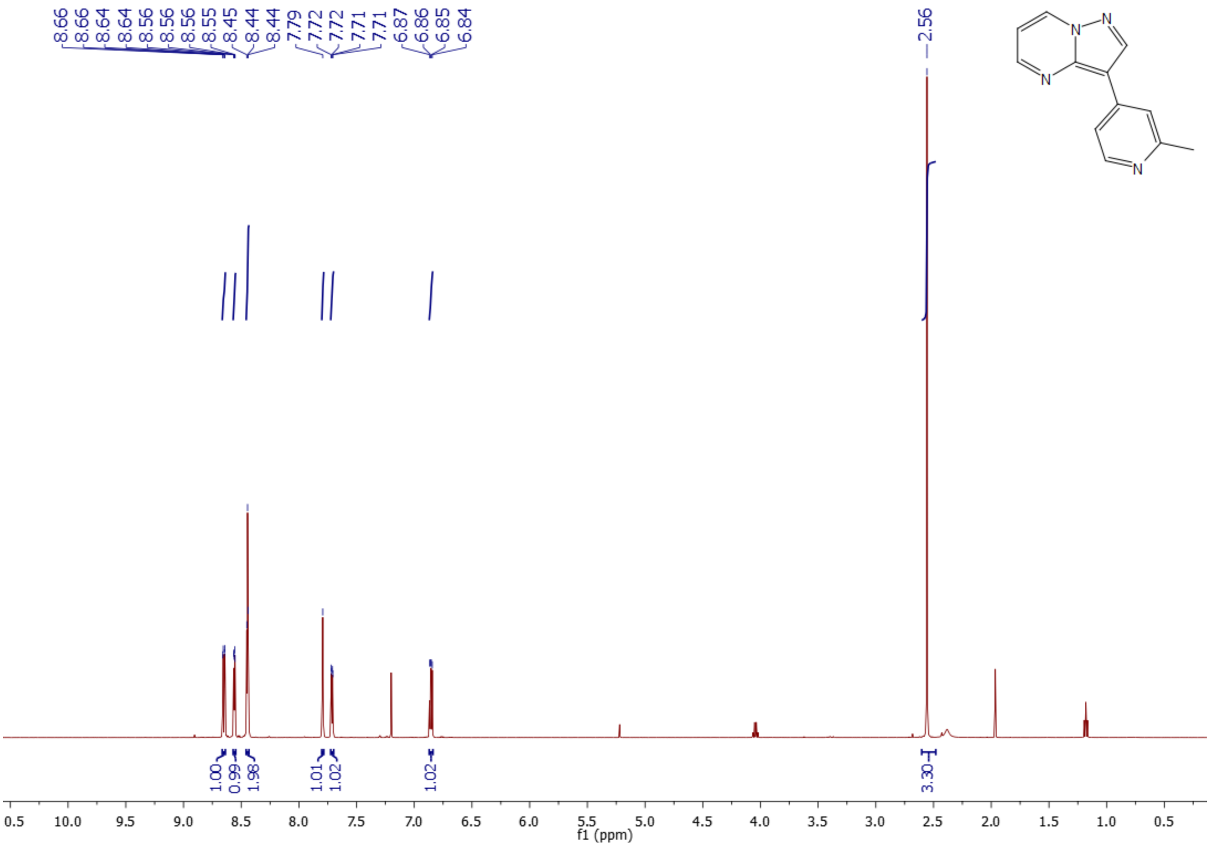

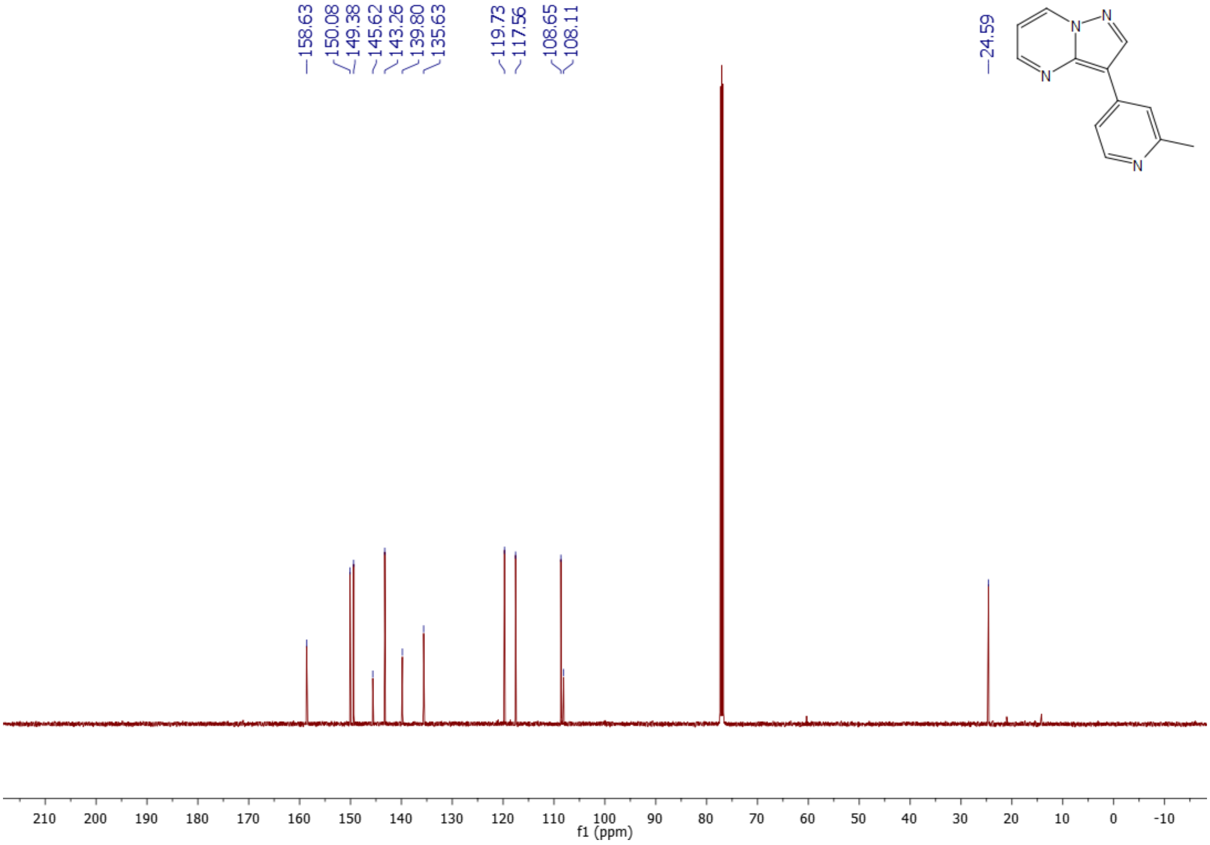

3-(2-fluoropyridin-4-yl)pyrazolo[1,5-a]pyrimidine, 3o

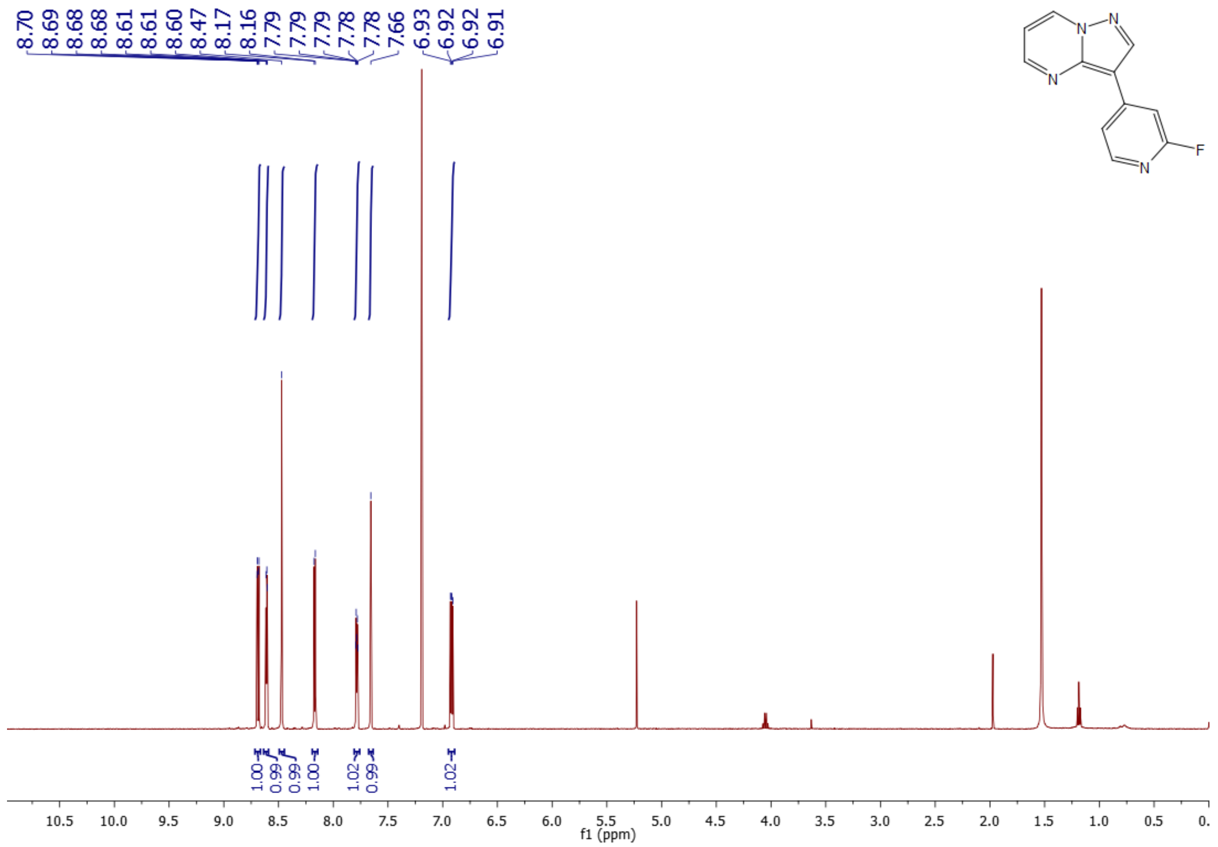

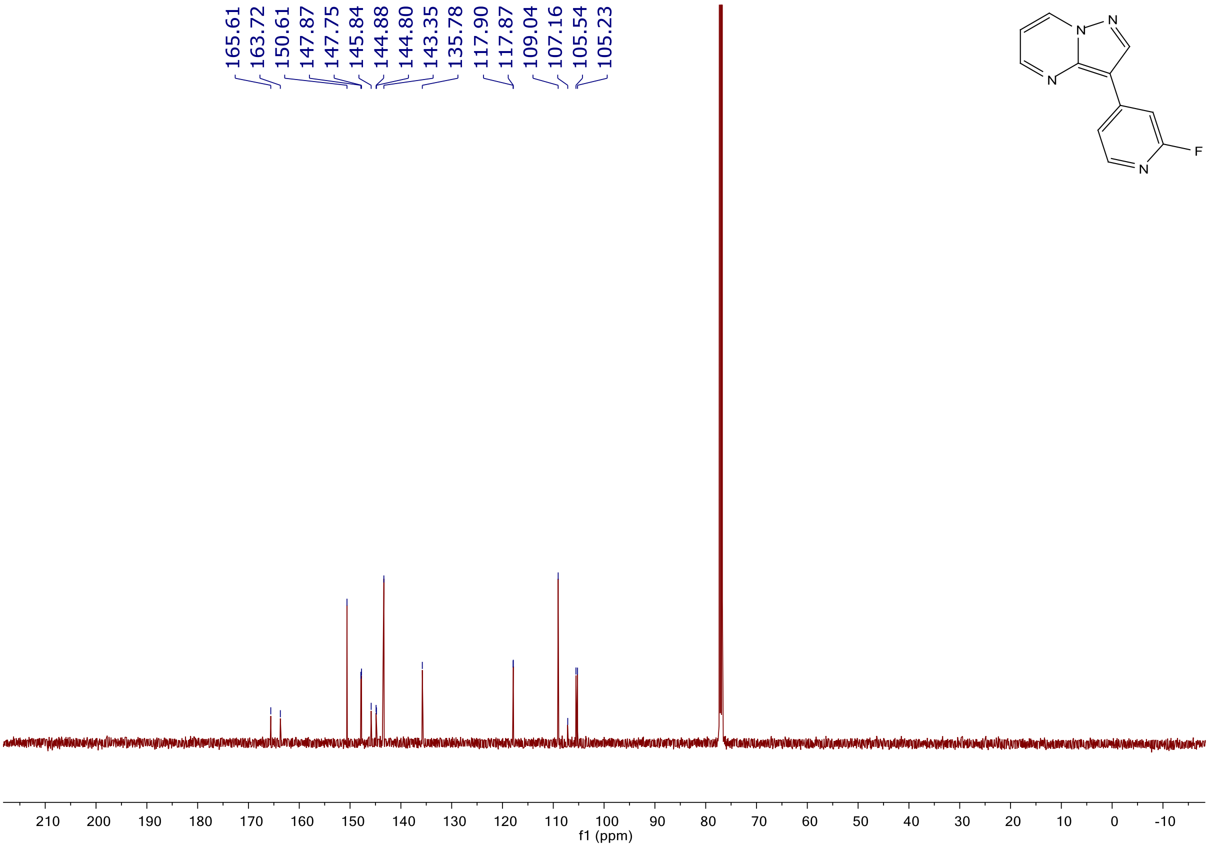

3-(pyridin-3-yl)pyrazolo[1,5-a]pyrimidine, 3p

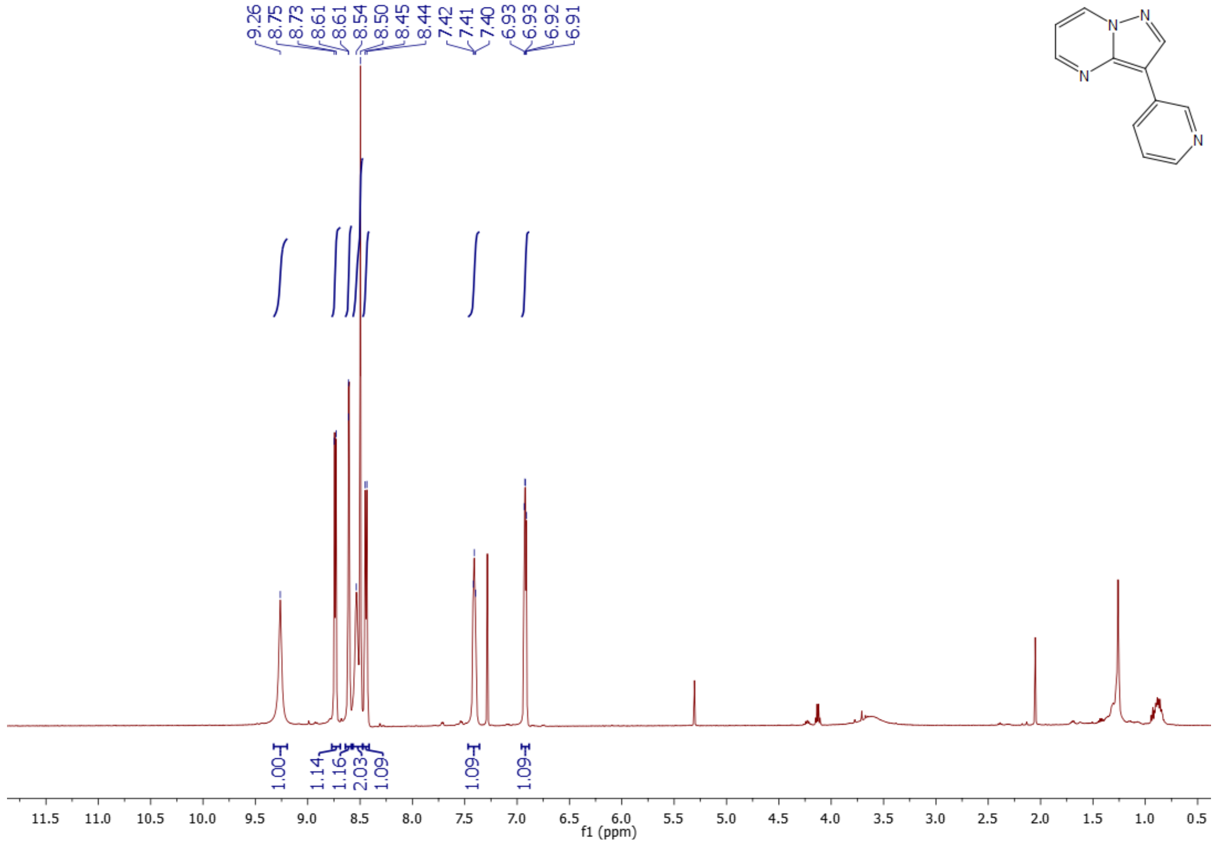

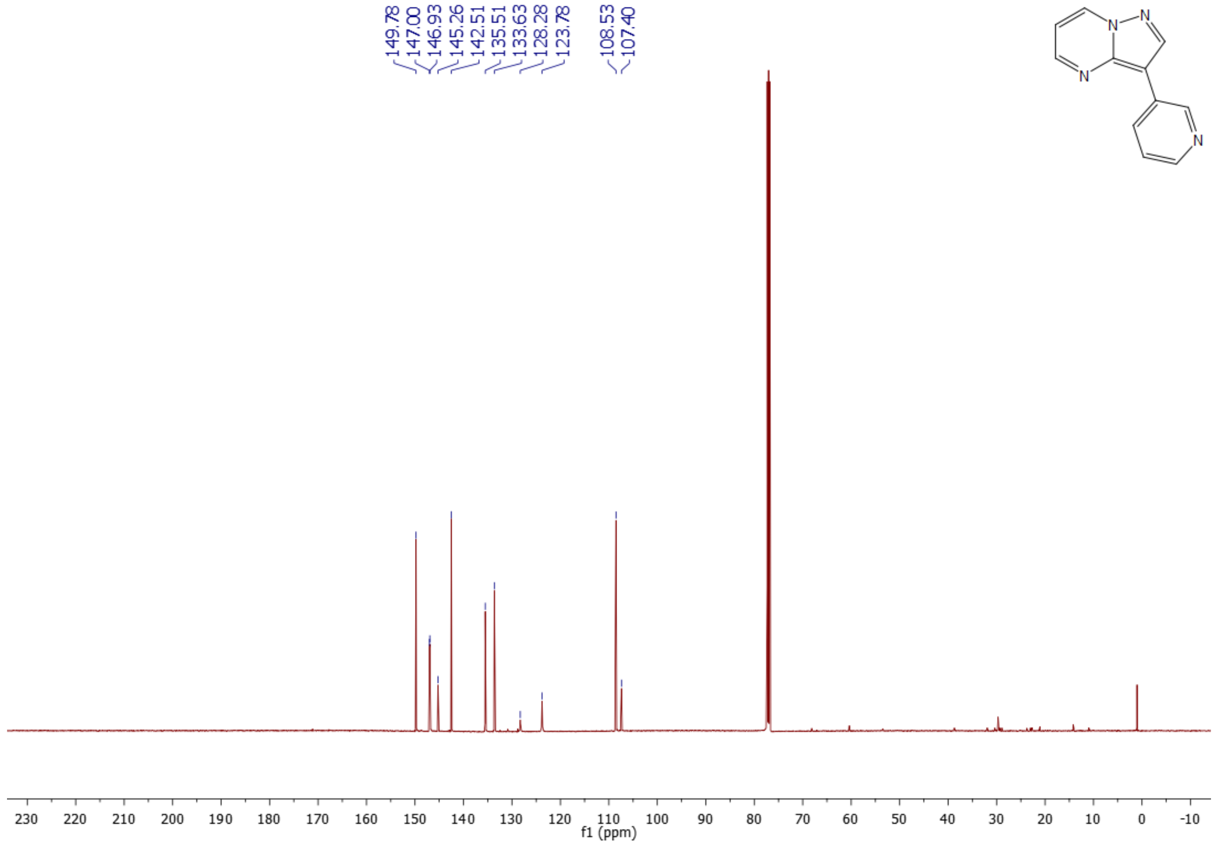

3-(pyrimidin-5-yl)pyrazolo[1,5-a]pyrimidine, 3q

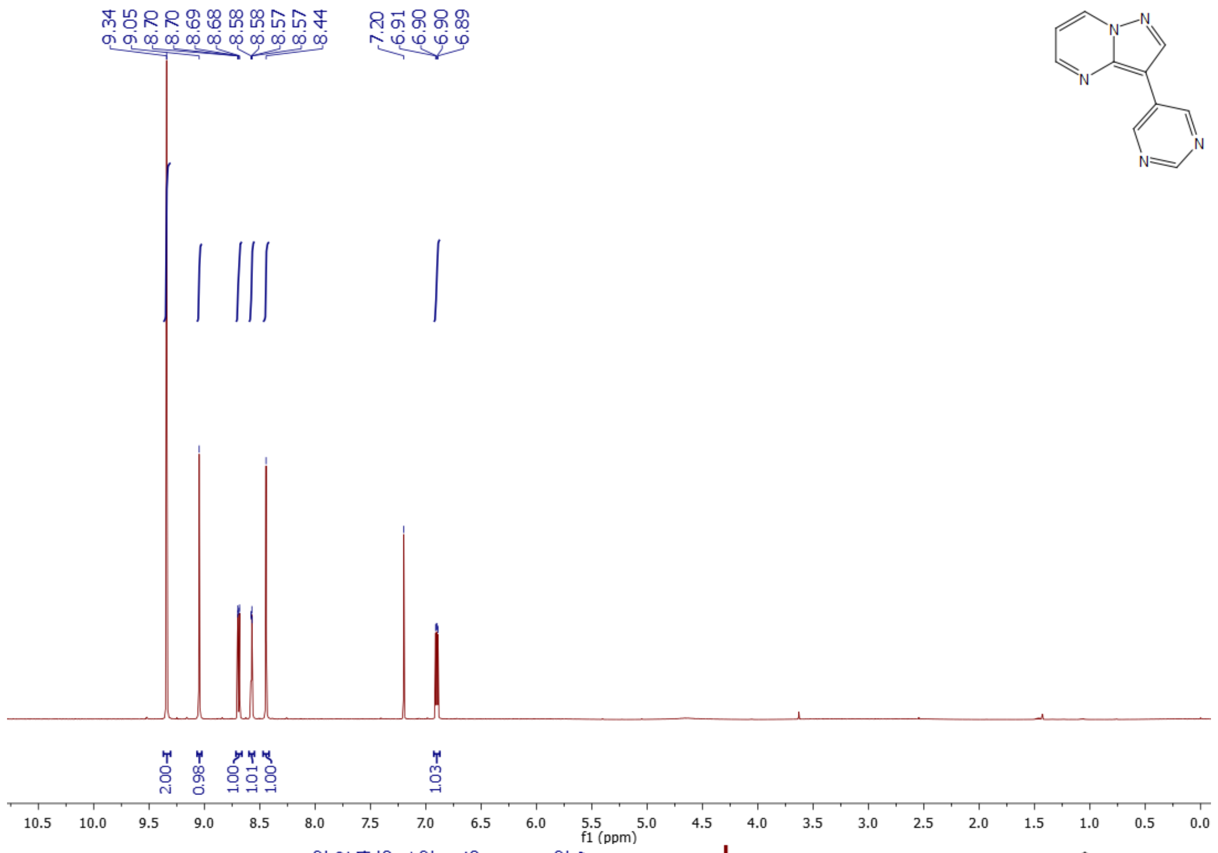

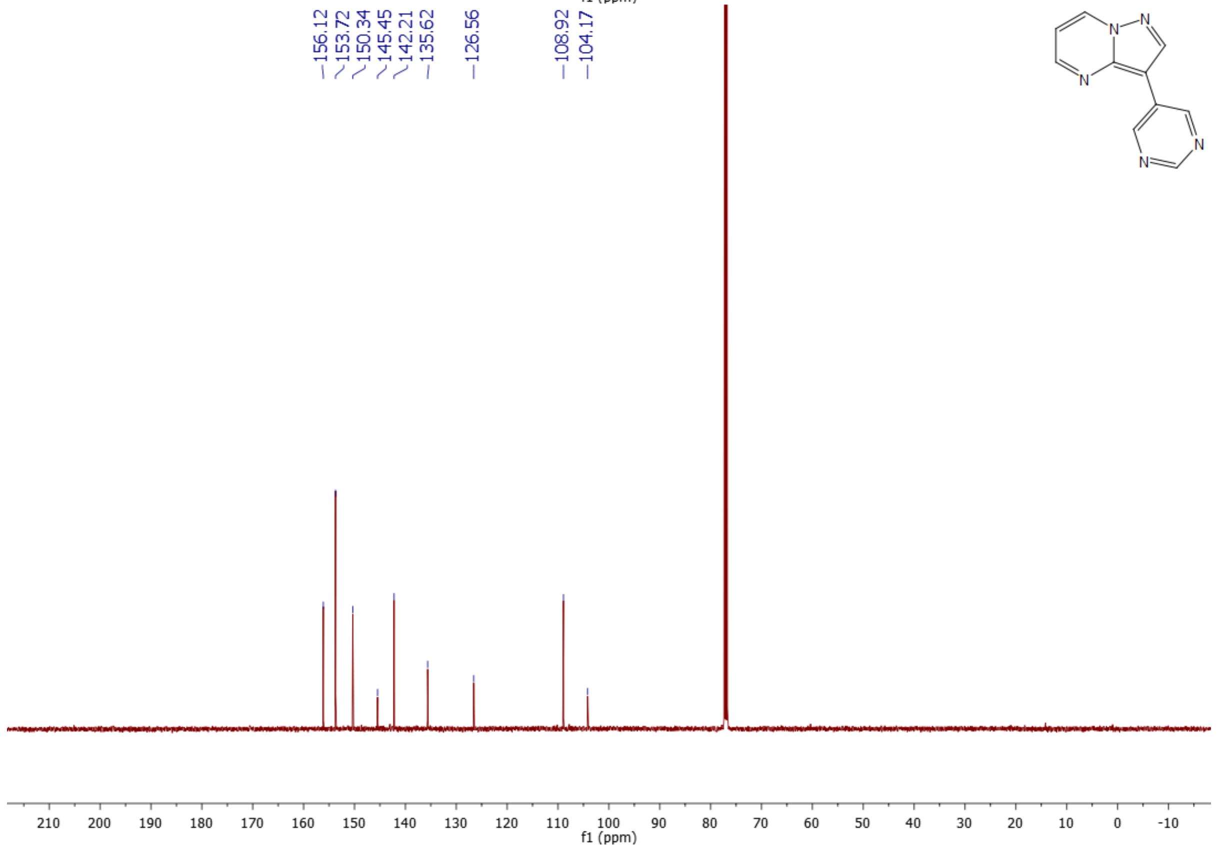

3,7-diphenylpyrazolo[1,5-a]pyrimidine, 4a

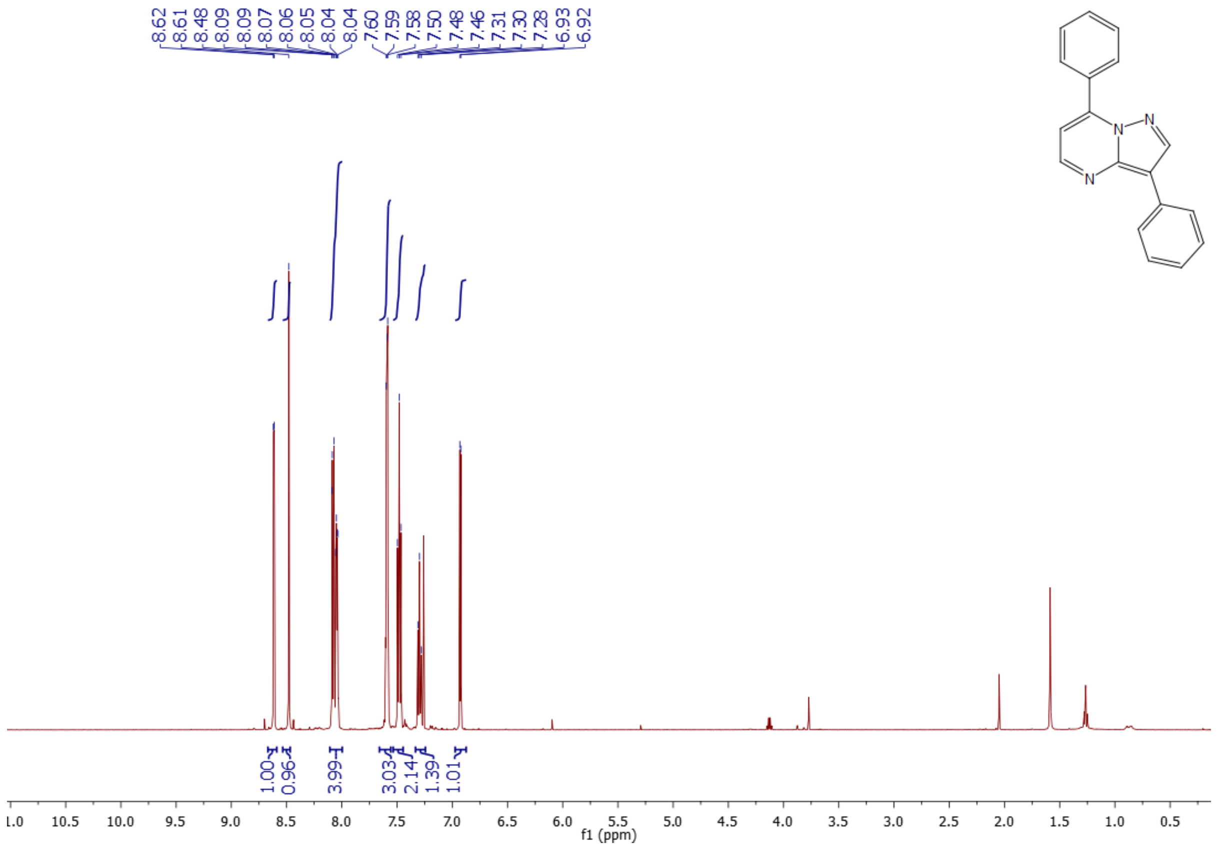

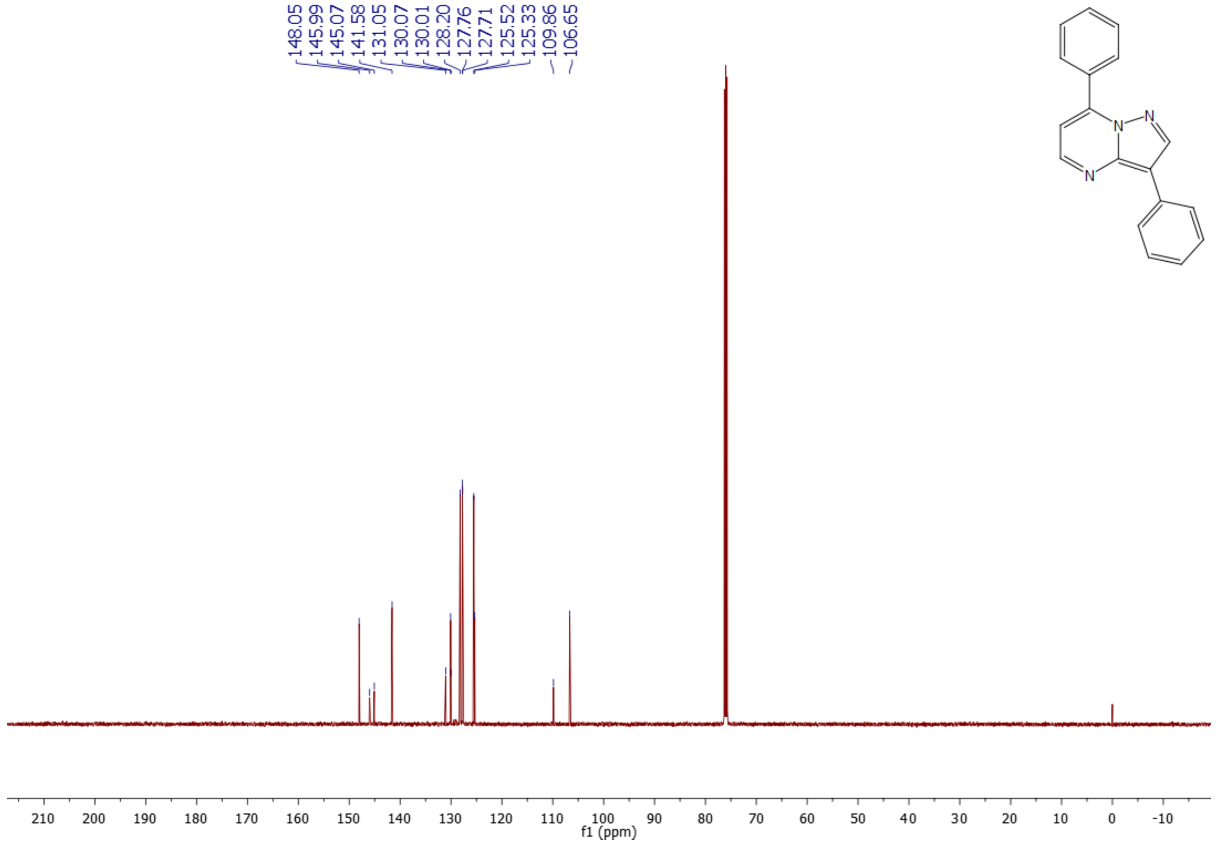

3-phenyl-7-(4-(trifluoromethyl)phenyl)pyrazolo[1,5-a]pyrimidine, 4b

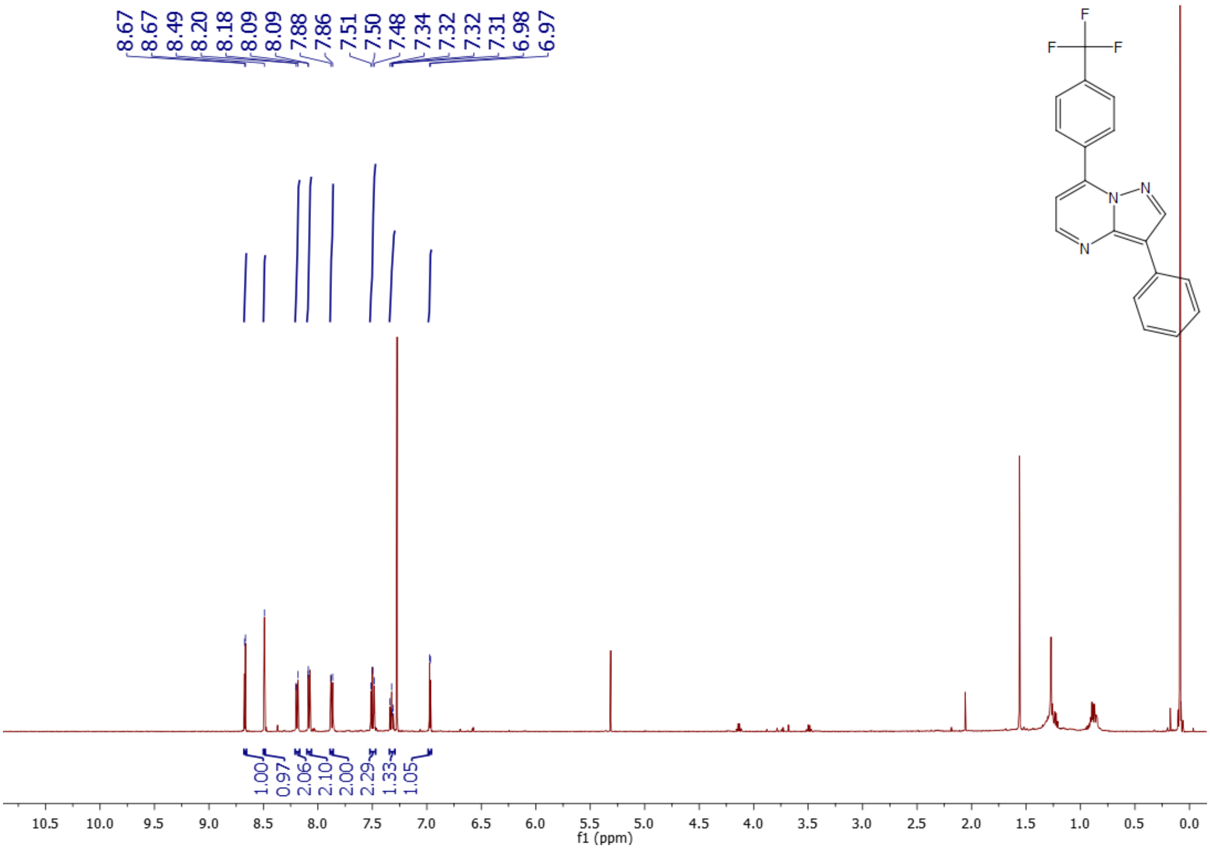
8.67
8.67
8.49
8.20
8.18
8.09
8.09
7.88
7.86
7.51
7.50
7.48
7.34
7.32
7.32
7.31
6.98
6.97
1.00
0.97
2.06
2.10
2.00
2.29
1.33
1.05
f1 (ppm)

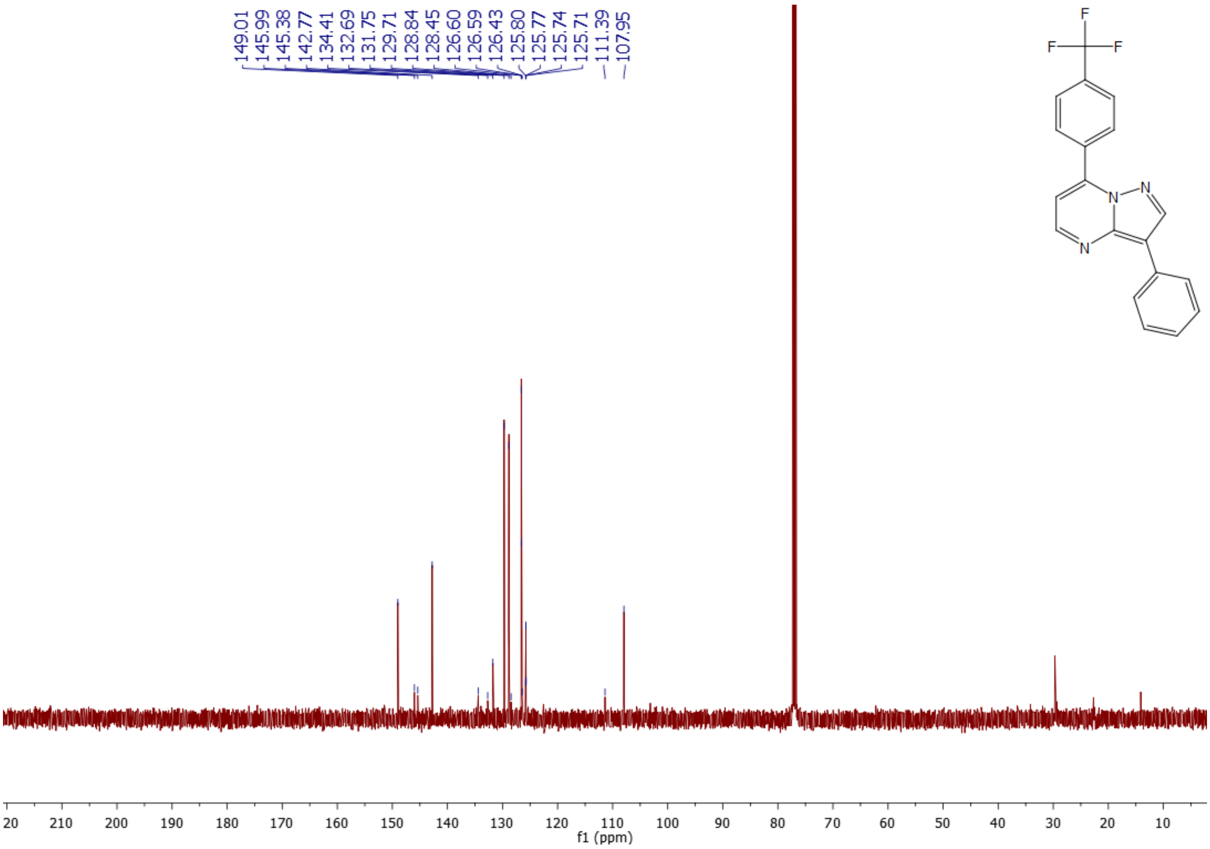
149.01
145.99
145.38
142.77
134.41
132.69
131.75
129.71
128.84
128.45
126.60
126.59
126.43
125.80
125.77
125.74
125.71
111.39
107.95
f1 (ppm)

9. References

[S1] (a) R. G. Parr, W. Yang, Density-Functional Theory of Atoms and Molecules; Oxford University Press, Oxford, **1989**.

[S2] Gaussian 09, Revision D.01, M. J. Frisch, G. W. Trucks, H. B. Schlegel, G. E. Scuseria, M. A. Robb, J. R. Cheeseman, G. Scalmani, V. Barone, B. Mennucci, G. A. Petersson, H. Nakatsuji, M. Caricato, X. Li, H. P. Hratchian, A. F. Izmaylov, J. Bloino, G. Zheng, J. L. Sonnenberg, M. Hada, M. Ehara, K. Toyota, R. Fukuda, J. Hasegawa, M. Ishida, T. Nakajima, Y. Honda, O. Kitao, H. Nakai, T. Vreven, J. A. Montgomery Jr, J. E. Peralta, F. Ogliaro, M. Bearpark, J. J. Heyd, E. Brothers, K. N. Kudin, V. N. Staroverov, T. Keith, R. Kobayashi, J. Normand, K. Raghavachari, A. Rendell, J. C. Burant, S. S. Iyengar, J. Tomasi, M. Cossi, N. Rega, J. M. Millam, M. Klene, J. E. Knox, J. B. Cross, V. Bakken, C. Adamo, J. Jaramillo, R. Gomperts, R. E. Stratmann, O. Yazyev, A. J. Austin, R. Cammi, C. Pomelli, J. W. Ochterski, R. L. Martin, K. Morokuma, V. G. Zakrzewski, G. A. Voth, P. Salvador, J. J. Dannenberg, S. Dapprich, A. D. Daniels, O. Farkas, J. B. Foresman, J. V. Ortiz, J. Cioslowski, D. J. Fox, Gaussian, Inc., Wallingford CT, **2013**.

[S3]. B3LYP functional: (a) A. D. Becke, *J. Chem. Phys.,* **1993**, *98*, 5648; (b) C. Lee, W. Yang, R. G. Parr, *Phys. Rev. B.,* **1988**, *37*, 785; (c) S. H. Vosko, L. Wilk, M. Nusair, *Can. J. Phys.,* **1980**, *58*, 1200; (d) P. J. Stephens, F. J. Devlin, C. F. Chabalowski, M. J. Frisch, *J. Phys. Chem.,* **1994**, *98*, 11623.

[S4]. Grimme's D2 dispersion correction: (a) S. J. Grimme, *Comput. Chem.,* **2004**, *25*, 1463; (b) S. J. Grimme, *J. Comput. Chem*., **2006**, *27*, 1787.

[S5] R. Krishnan, J. S. Binkley, R. Seeger, J. A. Pople, *J. Chem. Phys.,* **1980**, *72*, 650.

[S6]. (a) S. Miertus, E. Scrocco, J. Tomasi, *Chem. Phys.,* **1981**, *55*, 117; (b) G. Scalmani, M. J. Frisch, B. Mennucci, J. Tomasi, R. Cammi, V. J. Barone, *Chem. Phys.,* **2006**, *124*, 094107.

[S7]. This methodology has been shown to work well previously in calculating pKa's of heterocycles, see: K. Shen, Y. Fu, J. N. Li, L. Liu and Q. X. Guo, *Tetrahedron*, **2007**, *63*, 1568.

[S8] (a) G. Koleva, B. Galabov, J. I. Wu, H. F. Schaefer III, P. von R. Schleyer, *J. Am. Chem. Soc.* **2009**, *131*, 14722; (b) B. Galabov, G. Koleva, H. F. Schaefer III, P. v. R. Schleyer, *J. Org. Chem.* **2010**, *75*, 2813.

[S9] (a) L. A. Curtiss, M. P. McGrath, J. P. Blaudeau, N. E. Davis, R. C. Binning Jr., L. Radom, *J. Chem. Phys.* **1995**, *103*, 6104; (b) T. Clark, J. Chandrasekar, G. W. Spitznagel, P. v. R. Schleyer, *J. Comput. Chem.* **1983**, *4*, 294.
